# Polynuclear Complexes of Nd and Dy with N_2_O_3_ Donor Ligands: Solution Speciation and Selective Precipitation Studies

**DOI:** 10.1021/acs.inorgchem.5c03477

**Published:** 2025-10-24

**Authors:** Alex Falco, Alessia Panizzi, Matteo Melegari, Fabio Fornari, Monica Maffini, Matteo Tegoni, Angela Serpe, Nicola Demitri, Luciano Marchiò

**Affiliations:** † Department of Chemistry, Life Sciences and Environmental Sustainability, 9370 University of Parma, Parco Area Delle Scienze 17/A, Parma 43124, Italy; ‡ Department of Civil and Environmental Engineering and Architecture (DICAAR), and Research Unit of INSTM, 3111 University of Cagliari, Via Marengo 2, Cagliari 09123, Italy; § Environmental Geology and Geoengineering Institute of the National Research Council (IGAG-CNR), Piazza d’Armi, Cagliari 09123, Italy; ∥ Elettra − Synchrotron Light Source, S.S. 14 Km 163.5 in Area Science Park, Basovizza, Trieste 34149, Italy

## Abstract

This study explores the formation and selective separation of polynuclear neodymium and dysprosium complexes with N_2_O_3_ donor ligands derived from *N*,*N*-bis­(salicylidene)-1,3-diamino-2-propanol (H_3_L^H^). The research focuses on the structural characterization and solution speciation of Nd^3+^ and Dy^3+^ complexes by using ligands with different peripheral substituents: H_3_L^H^, H_3_L^p‑OMe^, and H_3_L^o‑tBu^. These substituents significantly influence the nuclearity of the resulting complexes. For Dy^3+^, single-crystal X-ray diffraction (SC-XRD) revealed a range of molecular architectures, from dinuclear to hexanuclear species, each with distinct solubility profiles, whereas for Nd^3+^ an heptanuclear molecular structure with H_3_L^o‑tBu^ was obtained. Separation experiments with Nd:Dy ratios of 1:1 and 4:1 demonstrated the ability of the ligands to give a partial selective precipitation of Nd^3+^ and Dy^3+^ complexes, depending on both metal identity and ligand structure. In particular, H_3_L^H^ achieved a separation factor (*S*_Nd/Dy_) of 12.0 (±2.0), concentrating Nd^3+^ in the solid phase. In contrast, H_3_L^o‑tBu^ favored Dy precipitation, yielding a separation factor of 20.0 (±4) after just 10 min. In various instances, the low separation factor values were ascribed to the formation of mixed-metal polynuclear species, which was confirmed through Electrospray Ionization Mass Spectrometry (ESI-MS) and by the structural characterization of a heteronuclear complex with H_3_L^o‑tBu^.

## Graphical abstract

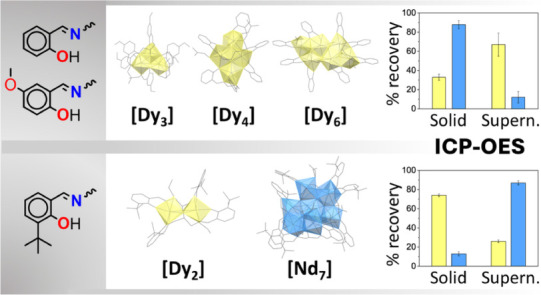

## 1 Introduction

Rare earth elements (REEs) play a central role in the advancement of telecommunication,^1,2^ lighting, and display technologies, such as lanthanide phosphor lamps and LEDs,^2–4^ and medical applications, including MRI bioimaging, where elements such as Nd, Yb, and Er are involved.^5,6^ Additionally, lanthanum- and cerium-based catalysts are widely used within the petroleum and automotive industries,^7–9^ while lanthanum and lutetium oxides are extensively employed in the glass industry as polishing agents. A few REEs have also been utilized for the production of permanent magnets (PM). Among other possible formulations, neodymium–iron-boron (NdFeB)-based permanent magnets are crucial for the establishment of energy production systems and energy utilization within the green transition.^10–13^ Indeed, these magnets are present in wind turbine rotors and are key components in the latest generation of electric and hybrid motors, just to mention a few. PMs are manufactured from a neodymium–iron-boron alloy, which consists of approximately 30 wt % neodymium and 0–10 wt % of other elements, such as dysprosium and praseodymium.^14^ The additional elements are incorporated to enhance the coercivity and thermal resistance of the magnet.^15,16^ However, the supply chain of REEs, from ore extraction to recovery from end-of-life (EoL) products, give rise to environmental and economic sustainability concerns.^17,18^ REEs are present in minerals including bastnaesite (REECO_3_F), monazite ((Th, REE)­PO_4_), and eudialyte (REEPO_4_), where they occur in the form of phosphates, carbonates, and silicates.^19,20^

China is responsible for 69% of the world’s REE production with a 9% increase from 43 million tons in 2022 to 47 million tons in 2023.^21,22^ A European Union report predicts that the demand for REEs will reach levels three to seven times higher than the current ones by 2040. Moreover, considering the production volumes and the estimated lifetime of REE-containing items, which range from 10 to 25 years,^23,24^ it is assumed that a sufficient amount of depleted items will be available for recycling, making them a valuable secondary source of REEs. Despite the significant advances in the field, a cost-competitive, environmentally friendly, and scalable approach for REE recycling has yet to be achieved.^25^ Currently, the majority of REE separation technologies are limited to laboratory and pilot-scale operations.^26^ Several methodologies have been proposed for the concentration of REEs, aiming at their recycling and recovery. The methods include hydro-(solvo-)­metallurgical and/or pyrometallurgical routes,^14^ the use of sorbents/membranes^27^ and ionic liquids,^28^ chromatography techniques,^29^ chemical coagulation, ion flotation, and more.^30–32^ Among these methods, leaching and solvent extraction are currently the two most widely used and established chemical separation techniques for REEs.^33–35^ In a recent study by Wang and coworkers,^36^ solvent extraction was employed to separate heavy rare earths from lighter ones. The researchers demonstrated that phenanthrolines in an octanol/water mixture can be used as an extracting agent. Through UV–vis spectroscopy, it was shown that these ligands had higher affinities for HREEs, which were dependent on the pH and contact time. Furthermore, the ligands were found to extract HREEs more effectively in the organic phase than did LREEs.

The previously mentioned technologies rely on the selective chemical discrimination of one rare earth element cation from another within the series, possible through metal complexation with appropriate ligands. Due to the different cation sizes, REEs can be separated as a result of the preferential formation of complexes with different nuclearity (monomers, dimers, or oligomers), hydration number, affinity to supramolecular entities, and even crystal packing, resulting in a different solubility of the complexes.^37–39^

*N*,*N*-bis­(salicylidene)-1,3-diamino-2-propanol-based compounds are well known in the literature and have been utilized for their antimicrobial activity,^40–42^ as well as their catalytic,^43–45^ magnetic, and luminescent properties.^46–49^ In these compounds, *N*,*N*-bis­(salicylidene)-1,3-diamino-2-propanol typically behaves as a pentadentate N_2_O′O_2_ or tetradentate N_2_O_2_ ligand, able to complex transition metals as well as lanthanides and uranium.^43,50–52^ In fact, Schiff bases have already been investigated for the extraction of base metals,^53^ as well as the separation of actinides from lanthanides^54,55^ and the separation of lanthanides from each other.^56,57^

In lanthanide (Ln) coordination, Ln^3+^ ions typically exhibit coordination numbers of seven or higher.^58^ As a result, a single *N*,*N*-bis­(salicylidene)-1,3-diamino-2-propanol molecule is generally unable to fully satisfy the coordination environment of these metals, often leading to the formation of complex polynuclear structures (metal-to-ligand ratios of 2:1, 4:4, 6:4, 9:4, 9:5, 16:4).^59–64^

Here, we explored the formation of various molecular entities through the reaction of Dy^3+^ and Nd^3+^ with three ligands belonging to the H_3_L^R^ class (Figure 1), with the aim of investigating a useful methodology for the separation of rare earth mixtures.

**Figure 1:**
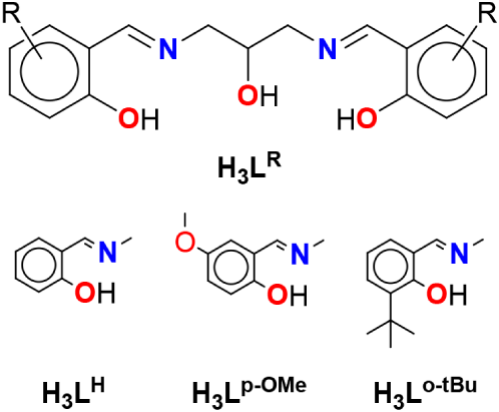
Molecular structures of the H_3_L^R^ ligands.

The small ligand array comprises the simplest of the series, which has unfunctionalized aromatic rings (H_3_L^H^), and the compound H_3_L^o‑tBu^ with bulky *t*-butyl groups close to the hydroxyl moiety of the peripheral aromatic ring. In H_3_L^p‑OMe^, the substituent is in the para position with respect to the phenolic oxygen atom, and it should not significantly alter the steric hindrance of the ligand during the metal coordination. On the other hand, the substituents may have a role in the formation of polynuclear structures where different ligands bridge multiple metal centers and the ligands can be intertwined. The structural characterization was obtained by single-crystal X-ray diffraction, which provided the molecular structures of six Dy, one Nd, and one mixed NdDy complexes (**1**–**8** in Table 1). Furthermore, an Nd and Dy separation was attempted based on the contrasting solubility behavior exhibited by their respective metal complexes. The different solubility of Nd and Dy complexes in various solvents could arise from the changes in the presence of a coordinated solvent molecules, the neutral or ionic nature of the complexes, or their mononuclear or polynuclear structures.^39,65,66^ In this study, reactions were carried out in ethanol by mixing the ligands (H_3_L^H^, H_3_L^p‑OMe^, or H_3_L^o‑tBu^) with lanthanide nitrate salts at 55 °C in the presence of triethylamine (TEA) as a base. Lanthanide nitrates in ethanol were considered as ideal feed solutions, mimicking the real cases of Ln_2_O_3_ mixtures dissolved by HNO_3_ before undergoing concentration and separation treatments.^67,68^ Two stoichiometric ratios of H_3_L^R^:Base:Nd:Dy were investigated, 1.05:3.15:0.5:0.5 and 1.05:3.15:0.8:0.2, with reaction times varied systematically. Notably, the Nd complexes of the ligands H_3_L^H^ and H_3_L^p‑OMe^ were sparingly soluble in the reaction environment, leading to their predominant distribution in the solid phase. In contrast, the Dy complex with ligand H_3_L^o‑tBu^ exhibited a reversed solubility, with an enrichment of Dy in the solid phase.^69^

**Table 1:** Summary of the Complexes with H_3_L^R^ Ligands

Formula	Compound
[Dy_3_(HL^p‑OMe^)_2_(L^p‑OMe^)(OH)(DMF)_2_]NO_3_·(DMF)_2_	**1**
[Dy_4_(HL^H^)(L^H^)_2_(OH)_2_(HCOO)(H_2_O)_2_(DMF)] [Dy_4_(HL^H^)(L^H^)_2_(OH)_2_(HCOO)(H_2_O)(DMF)_2_](NO_3_)_2_·(H_2_O)_0.2_·(DMF)_2.75_	**2**
[Dy_6_(L^H^)_4_(OH)_4_(NO_3_)_2_(H_2_O)_2_]·(CH_3_COCH_3_)_1.66_	**3**
[Dy_6_(L^p‑OMe^)_4_(OH)_4_(NO_3_)_2_(H_2_O)_2_]·(H_2_O)_0.5_·(CH_3_COCH_3_)_2.33_	**4**
[Dy_2_(L^o‑tBu^)_2_(EtOH)_2_]·EtOH	**5**
[Dy_2_(L^o‑tBu^)_2_(THF)_2_]·THF	**6**
[NdDy(L^o‑tBu^)_2_(EtOH)_2.69_]·EtOH·(H_2_O)_0.2_	**7**
Et_3_NH[Nd_7_(L^o‑tBu^)_4_(OH)_6_(NO_3_)_4_(EtOH)_4_]·(EtOH)_8_	**8**

## 2 Results and Discussion

The H_3_L^R^ ligands were obtained as previously reported, through a one-pot one-step synthesis between 1,3-diaminopropan-2-ol and differently functionalized salicylaldehydes.^45,70^ The ligands were typically isolated in good yields (80–95%). The presence of the imino function has implications both in the chemical reactivity and the coordination properties of the ligands. The ligands were resulted to be stable in 96% ethanol (see Figure S25), absolute ethanol, and other organic solvents such as acetone, methanol, acetonitrile, tetrahydrofuran, and *N*,*N*-dimethylformamide. However, they rapidly degrade in various aqueous environments: distilled water, oxalic acid (50 mM), and Hepes buffer (50 mM) (Figure S26). From a structural point of view, the imino functions confer a significant rigidity to the ligand, since the only conformationally flexible moiety is represented by the central aliphatic fragment bearing the hydroxyl group. The H_3_L^R^ molecule can be deprotonated on the hydroxyl functions (phenolic, p*K*_a_ range 9–10 in water as a function of the substituents; aliphatic, p*K*_a_ > 16 in water),^71^ giving rise to a −2 (HL^R^) or −3 (L^R^) charged ligand that could provide a N_2_O_3_ donor set to the metal center.

Ligands H_3_L^R^ were investigated as complexing agents for Nd^3+^ and Dy^3+^ to identify structural differences in the resulting molecular entities. The reactions were conducted using a 1:1 M:H_3_L^R^ stoichiometric ratio, regardless of the stoichiometry of the final species, and in the presence of three equivalents of triethylamine (p*K*_b_ of 3.4 at 298 K in water) to facilitate the ligand deprotonation. The synergistic effect of metal complexation and base addition was usually sufficient to promote the deprotonation of the phenolic groups, leading to the formation of the complexes.

### 2.1 Description of the Crystal Structures

The characterization of the structural properties of the complexes represented an important aspect in the interpretation of the diverse characteristics exhibited by the compounds. The reported polynuclear structures feature several small molecular entities completing the coordination of the metal centers, such as solvent molecules or nitrates. Nevertheless, the structures provide useful insight into the possible speciation mechanisms that occur in solution. The full molecular formula of the complexes as obtained by the X-ray diffraction is reported in Table 1 along with a simplified naming scheme (compounds **1**–**8**). Here we provide a detailed description of the eight compounds, while the crystallographic data and some selected geometric parameters are reported in Tables S1–S6 and S10–S13. The coordination environments of the Dy^3+^ and Nd^3+^ ions were determined by analysis of the continuous shape measurement parameters of the metal centers using the SHAPE 2.1 software (Tables S7–S9).^72^

The structures of some of the possible complexes observed in solution were attained experimentally: dinuclear (compounds **5**, **6**, and **7**), trinuclear (compound **1**), tetranuclear (compound **2**), hexanuclear (compounds **3** and **4**), and heptanuclear (compound **8**). Even though all the polynuclear species comprised solvent molecules of coordination, nitrates, and in some cases the ligands were not fully deprotonated (compounds **1** and **2**), a close inspection of the different systems showed the presence of significant underlying similarities in the complexes. In particular, a common [M_3_(μ_3_-OR)­(μ_3_-OH)­(μ_2_-O)_3_] substructure can be observed in the structures of all of the complexes, except for the L^o‑tBu^ structures (compounds **5**–**8**) probably due to the steric hindrance of the *tert*-butyl groups. Interestingly, the tetranuclear compound **2**, and the hexanuclear compounds **3** and **4**, can be envisioned as an extension of the [M_3_(μ_3_-OR)­(μ_3_-OH)­(μ_2_-O)_3_] unit by the addition of one (in the case of the tetrametallic structure) or three (in the case of the hexametallic structures) metal centers, plus a variable number of bridging ligands, hydroxide ions, and nitrates (Figure 2). Additionally, the coordination of the ligand, which presents a flexible central region, and two rigid aromatic systems can be described as two, more or less planar and tridentate, chelating moieties with the central alcoholic oxygen shared between the two. The angle between the two regions, the number of bridged metal ions, the degree of deprotonation, and the overall spatial disposition of the ligand can be grouped by the description of four main conformations (Figure 2g).

**Figure 2:**
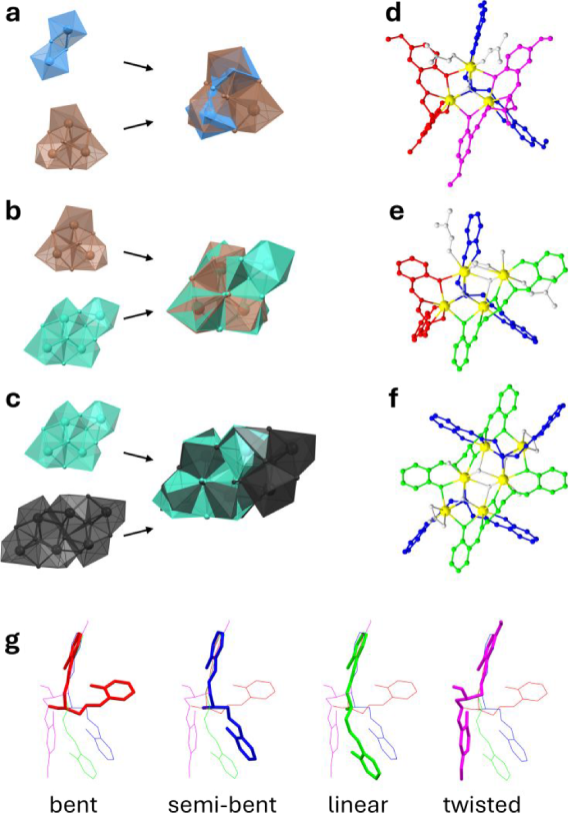
Overlay of the crystal structure highlighting the metal coordination of a) complexes **5** (dinuclear, in light blue) and **1** (trinuclear, in brown), b) complexes **1** (in brown) and **2** (tetranuclear, in emerald), and c) complexes **2** (in emerald) and **3** (hexanuclear, in black). Oxygen atoms bridging multiple metal centers are reported as balls and sticks. Structures of complexes d) **1**, e) **2**, and f) **3**, highlighting the different conformation of the ligands as in g): bent conformation (in red), semibent conformation (in blue), linear conformation (in green), and twisted conformation (in magenta). The ligand conformations were taken from the structures comprising the ligand L^H^ in complex **3** (bent, semibent, and elongated) and the ligand HL^p‑OMe^ in complex **2** (twisted).

In the bent conformation, the ligand is not deprotonated on the central alcoholic oxygen and the two tridentate regions are wrapped around a single metal ion with one of the phenolic oxygens bridging a second metal ion.

In the semibent conformation, the ligand is fully deprotonated. The two tridentate regions chelate two different metal ions with the central alkoxy oxygen exhibiting a μ_3_-coordination between the two metal ions and an additional one. This central alkoxy oxygen is the μ_3_-OR forming the trinuclear subunit found in the complexes. All ligands in the structures of the dinuclear *tert*-butyl complexes (compounds **5**, **6**, and **8**) have a semibent spatial disposition, however the central alkoxy oxygen atom bridges between only two metals.

In the linear conformation, the ligand is fully deprotonated. The two tridentate regions chelate two distinct metal ions and the central alkoxy oxygen is bridged between the two. Additionally, at least one of the phenoxy oxygen atoms coordinates an additional metal, making this ligand μ_3_ if only one of them is bridging (compound **2**) or μ_4_ if both are bridging (compounds **3** and **4**).

Lastly, in the twisted conformation, found only in compound **1**, the ligand is not deprotonated on the alcoholic oxygen, and it does not coordinate any metal. The two tridentate regions are chelating the same metal center and both phenolic oxygen atoms are bridging two metals each.

It is reasonable to hypothesize the presence of a pathway that, starting from a common substructure, leads to the formation of species with higher nuclearities in a stepwise mechanism. This can be inferred by the close matching between the metal positions and ligand conformations found starting from the trinuclear complexes up to the hexanuclear complexes (Figure 2). The growth of the polynuclear entities is possible according to the flexibility of the central moiety of the ligand and the ability of the phenolate and the alkoxy functions to bridge between multiple metal centers. Increasing the nuclearity of the system thus leads to an increase in the number of bridged metals for each ligand (μ value, Table S6), coupled with the insertion of additional μ_3_-OH ions, except in the case of the structures of L^o‑tBu^.

A scheme of the coordination mode of the three ligands in the eight complexes and the overall representations of the conformations found in previously characterized Ln^3+^ complexes with H_3_L^R^ ligands are shown in Figures S7 and S8.

#### 2.1.1 Trinuclear Complex

Compound **1** crystallizes in the monoclinic crystal system and space group *P*2_1_/*c*. The trinuclear complex consists of three Dy^3+^ ions coordinated by one L^p‑OMe^ and two HL^p‑OMe^ ligands, one μ_3_-OH^–^ ion, and two DMF molecules to complete the metal coordination. The dysprosium atoms are octa-coordinated, with Dy1 and Dy3 exhibiting a triangular dodecahedral geometry (N_3_O_5_ and N_2_O_6_ coordination, respectively), while Dy2 displays a bicapped trigonal prismatic geometry (N_1_O_7_ coordination) (Figure 3) where the capping positions are represented by the central alcoholic and alkoxy oxygen atoms of two different ligands, nondeprotonated O1 and deprotonated O2, respectively. The hydroxide ion (μ_3_-O1H) resides over the plane formed by the three metal centers with average bond distances of 2.39(5) Å (displacement 1.21 Å from the metal mean plane). The μ_3_-O alkoxide of the ligand is situated on the other side of the metal plane with average bond distances of 2.38(3) Å (displacement −1.20 Å). The Dy–Dy distances in the [M_3_(μ_3_-OR)­(μ_3_-OH)­(μ_2_-O)_3_] subunit are close to each other (3.56(1) Å), and the three metals form an isosceles triangle (angles 59.9–60.3°). The μ_2_-O atoms in the subunit are on the same side of the metals’ mean plane as the μ_3_-OR alkoxide (displacement −0.29(4) Å), with average distances of 2.35(4) Å. The three ligands, represented in shades of gray in Figure 3, are differently distributed in the metal coordination sphere as they exhibit three distinct conformations. The bent ligand is doubly deprotonated (HL^p‑OMe 2–^) (dark gray in Figure 3, red in Figure 2) and is predominantly folded around Dy3, however, one phenolate oxygen atom is bridging between Dy3 (2.312(3) Å) and Dy2 (2.41(1) Å). The semibent ligand (gray in Figure 3, blue in Figure 2) is fully deprotonated (Figure S9) and in this case the central alkoxy oxygen atom (O1) is bridging all three metal centers with bond distances in the range [2.355(1)–2.411(9) Å]. One tridentate moiety chelates Dy1 (2.509(4) and 2.22(1) Å for Dy–N and Dy–O distances, respectively), while the other coordinates Dy2 (2.550(8) and 2.200(6) Å for Dy–N and Dy–O distances, respectively). The twisted ligand (light gray in Figure 3, purple in Figure 2), having C3 as the central carbon atom, is doubly deprotonated (HL^p‑OMe 2–^). For this ligand, two bidentate moieties chelate Dy1 (average Dy–N distance of 2.53(1) Å, and average Dy–O distance of 2.33(1) Å). The two phenolate oxygen atoms bridge between Dy1 and another metal ion each (Dy2 and Dy3, respectively). The central nondeprotonated hydroxyl group does not coordinate any metal and was found disordered over two positions that were refined with site occupancy factors of 0.56 and 0.44, respectively. Given the fact that two ligands are doubly deprotonated and one is triply deprotonated, an additional nitrate anion and one central hydroxide ion are present to balance the overall charge of the complex. The nitrate anion is present in the second coordination sphere and was found disordered over two positions (0.82 and 0.18 site occupancy factors, respectively), forming hydrogen bonds with the central hydroxyl oxygen of the bent ligand and the hydroxide ion (O–O_nitr_ distances of 2.750(3) and 2.920(3) Å, respectively) (Figure S9). Finally, the coordination environment of Dy2 is completed by two DMF molecules and a total of two noncoordinated DMF molecules (disordered over 3 positions with site occupancy factors of 1, 0.5, and 0.5, respectively) were found in the structure.

**Figure 3:**
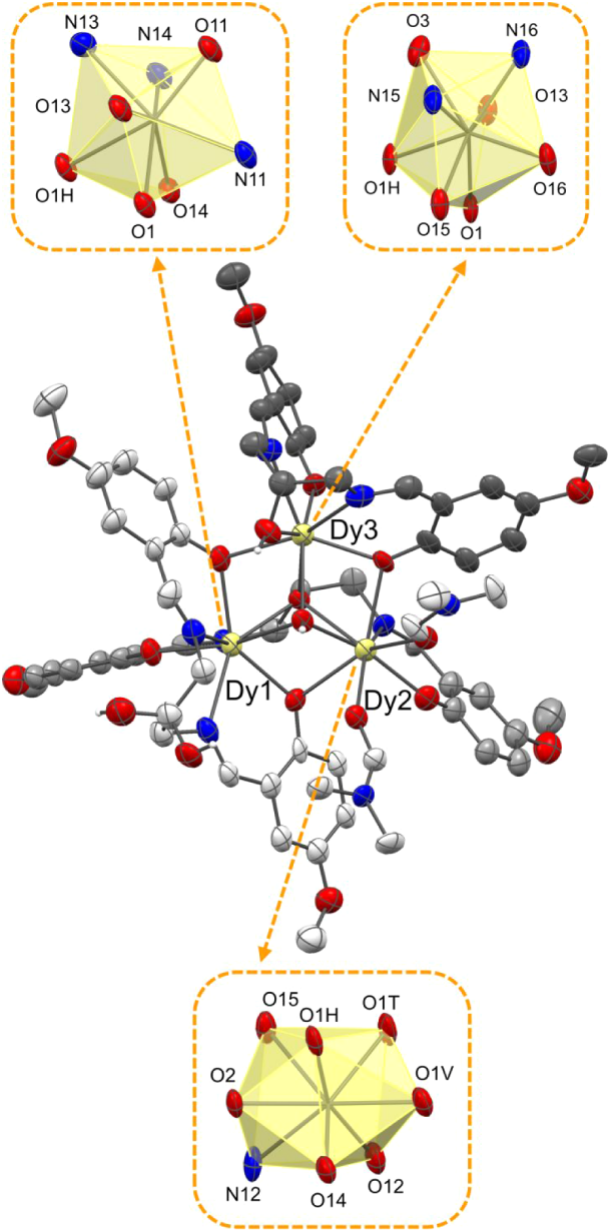
Molecular structure of the trinuclear complex [Dy_3_(HL^p‑OMe^)_2_(L^p‑OMe^)­(OH)­(DMF)_2_]­NO_3_(DMF)_2_ (**1**). Solvent of crystallization and the hydrogen atoms (except for the hydroxylic groups O3, O2/O2A, and the hydroxide ion O1H) were removed for clarity. Thermal ellipsoids were depicted at the 30% probability level. The triangular dodecahedral geometries of Dy1 and Dy3 and the bicapped trigonal prismatic geometry of Dy2 are highlighted. Dy (pale yellow), N (blue), O (red), C (dark gray, gray, and light gray), H (white).

#### 2.1.2 Tetranuclear Complexes

Complex **2** crystallizes in the triclinic crystal system and space group *P*-1. The asymmetric unit consists of two independent tetranuclear complexes, each having similar metal coordination environments and ligand dispositions. Moreover, the molecular entities are held together by two hydrogen bonds between a coordinated water molecule and phenolic oxygen atoms (Figure S13). Due to the high structural similarity between the two complex entities, they will be described together, as the only noteworthy difference is the presence of two coordinated water molecules in one entity as opposed to one DMF and one water molecule in the other (Figure S10). The complex (Figure 4) can be envisioned as being obtained by the insertion of a Dy^3+^ ion (Dy41) on the trinuclear complex described earlier, this time with H_3_L^H^ as the ligand. The overall spatial disposition of the ligands remains the same with the difference that the twisted ligand in complex **3**, which was not protonated, is now in a linear conformation with the central alkoxy oxygen atom deprotonated and bridging between two metal centers. The two planar tridentate moieties chelate two different metals (Dy11 and Dy41) with one of the phenolates bridging an additional metal (Dy31). One additional hydroxide ion is present in the structure, bridging between the newly inserted metal ions, Dy11 and Dy21. Except for Dy11, which presents a bicapped trigonal prismatic geometry (N_2_O_6_ coordination with the capping positions occupied by the central alkoxy oxygen atom of two different ligands), all Dy atoms are octa-coordinated, exhibiting a triangular dodecahedral geometry (N_1_O_7_, N_2_O_6_, and N_1_O_7_ coordination for Dy21, Dy31, and Dy41, respectively). The coordination environment of Dy41 is completed by two water molecules and a bidentate formate ion (probably derived from DMF decomposition) bridging to Dy21, which also presents a coordinated DMF molecule. The complex has an overall positive charge, which is balanced by the presence of a nitrate anion in the second coordination sphere, disordered over two positions modeled with occupancy site factors of 0.70 and 0.30, respectively. The nitrate ion is hydrogen bonded to the nondeprotonated alcoholic moiety of the bent ligand and one of the central hydroxide ions of the trinuclear subunit (average distances of 2.8(2) Å) (Figure S11). The hydroxide ion of the primary subunit ([M_3_(μ_3_-OR)­(μ_3_-OH)­(μ_2_-O)_3_]) resides over the plane formed by the three metal centers with average bond distances 2.37(3) Å (displacement 1.12(1) Å from the metals’ mean plane), while the μ_3_-O alkoxide atoms of the ligands are situated on the other side of the metals’ plane (average bond distances of 2.39(2) Å, displacement −1.24(1) Å). For the secondary subunit ([M_3_(μ_3_-OH)­(μ_2_-O)_3_]), the Dy–Dy distances are slightly longer than on the primary one (3.8(2) and 3.56(1) Å, respectively), and the triangular shape is slightly distorted (angles 53.5–67.3° and 59.1–61.0° in the primary and secondary subunits, respectively). The hydroxide ion of the secondary subunit (μ_3_-O2H) resides under the plane formed by the three metal centers with average bond distances 2.39(3) Å (displacement −0.92(2) Å from the metals’ mean plane). The μ_2_-O atoms are on opposite sides of the metals’ mean plane from the μ_3_-OH, with no significant distance differences in the two subunits, but with different displacements (−0.2(2) Å) and 1.2(3) Å for the primary and secondary subunits, respectively) (Figure S12). Overall, the four Dy ions are not coplanar, and the angle between the planes formed by the two [Dy_3_] subunits is 32.1°.

**Figure 4:**
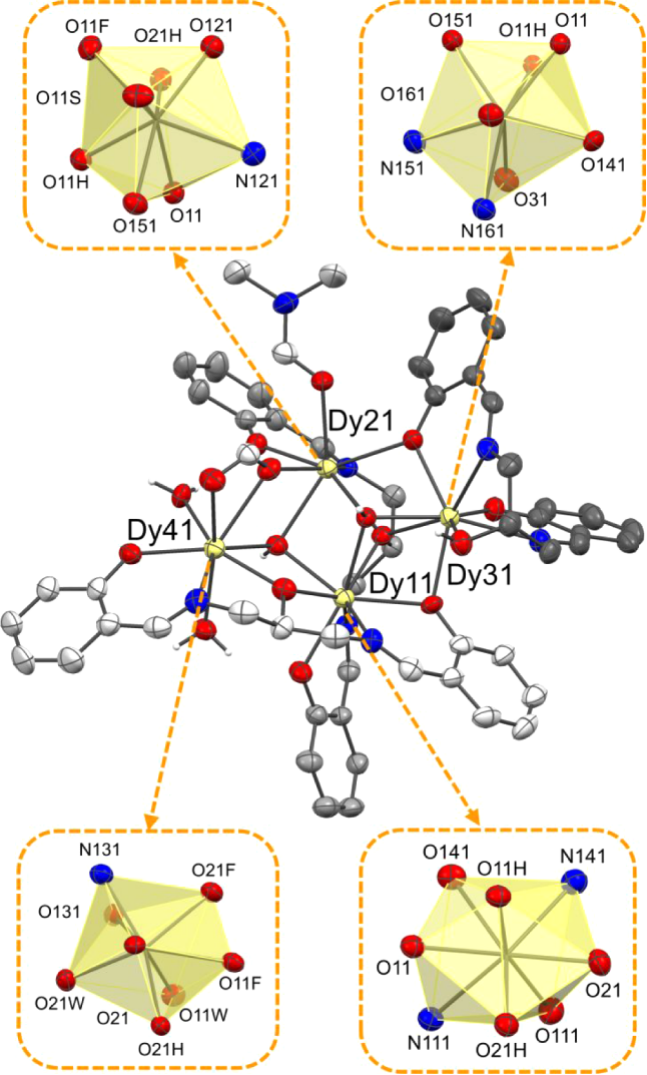
Molecular structure of complex [Dy_4_(HL^H^)­(L^H^)_2_(OH)_2_(HCOO)­(H_2_O)_2_(DMF)] [Dy_4_(HL^H^)­(L^H^)_2_(OH)_2_(HCOO)­(H_2_O)­(DMF)_2_]­(NO_3_)_2_·(H_2_O)_0.2_(DMF)_2.75_ (**2**). Solvent of crystallization and the hydrogen atoms (except for the hydroxide ions O11H, O21H, the water molecules O11W, O21W, and the hydroxyl group O31) were removed for clarity. The bicapped trigonal prismatic geometry of Dy11 and the triangular dodecahedral geometries of Dy21, Dy31, and Dy41 are highlighted. Thermal ellipsoids were depicted at the 30% probability level. Dy (pale yellow), N (blue), O (red), C (dark gray, gray, and light gray), H (white).

#### 2.1.3 Hexanuclear Complexes

Compounds **3** and **4** crystallize in the orthorhombic *Pbca* and monoclinic *P*2_1_/*n* space groups, respectively. The structure of complex **4** (Figures S15 and S16) is essentially equivalent to that of complex **3** (an overlay of the two structures is reported in Figure S17), for this reason only the structure of complex **3** will be described (Figure 5). The complex consists of six Dy^3+^ ions and can be envisioned as being obtained by the insertion of two additional Dy^3+^ ions (Dy1′ and Dy3′ symmetry code′: (1 – *x*; 1 – *y*; 1 – *z*)) on the tetranuclear complex described earlier or by the merging of two trinuclear complexes. Overall, four triply deprotonated L^H^ ligands and four μ_3_-OH hydroxide anions are present in the complex. Three metals are present in the asymmetric unit, and the rest of the complex is generated by symmetry (inversion center). The spatial disposition of the ligands is similar to that of the tetranuclear complex, with the difference that the bent ligand is not present. Additionally, the linear ligand is now bridging between a total of four metals instead of three (both phenolates present a μ_2_-coordination, Dy1 and Dy3 for the first one and Dy2′ and Dy3′ of the second one) connecting the symmetry-related halves of the hexanuclear complex. Four subunits can be identified in the structure: two primary and two secondary ones as in the case of the tetranuclear complex. All Dy atoms are octa-coordinated, exhibiting bicapped trigonal prismatic geometries (N_2_O_6_, N_1_O_7_, and N_1_O_7_ coordination for Dy1, Dy2, and Dy3, respectively), with the capping positions represented by the O1 and O2, O1, and O15, O14 and O2H for Dy1, Dy3, and Dy2, respectively (Figures 5 and S14). The charge balance is provided by the presence of two k^2^-nitrate ions (in the first coordination sphere of Dy3 and Dy3′) and the coordination environment of Dy2 is completed by one water molecule. As in the tetranuclear structure (complex **1**), the hydroxide ion of the primary subunit (μ_3_-O1H) resides over the plane formed by the three metal centers (average bond distances 2.37(4) Å and displacement of 1.18 Å from the metals’ mean plane), while the μ_3_-OR alkoxide of the ligand is situated on the other side of the metals’ plane (average bond distances 2.41(3) Å, displacement −1.28 Å), along with the μ_2_-O atoms (average bond distances 2.37(5) Å, displacement −0.30(4) Å). The Dy–Dy distances in the secondary subunit are slightly longer than in the primary one (3.8(2) and 3.55(3) Å, respectively), and the triangular shape is slightly distorted (angles 54.1–65.6° and 59.3–61.0° in the secondary and primary subunits, respectively) in a similar manner to the tetranuclear complex. The hydroxide ion of the secondary subunit (μ_3_-O2H) resides under the plane formed by the three metal centers (average bond distances 2.40(3) Å, displacement −1.00 Å from the metals’ mean plane), while the μ_2_-O atoms are on the opposite side (average bond distances 2.33(7) Å, displacement 1.2 Å). Overall, the six Dy ions are not coplanar, but the angle between the planes formed by the primary and secondary [Dy_3_] subunits is 25.0°, while the two symmetry-related secondary subunits are coplanar.

**Figure 5:**
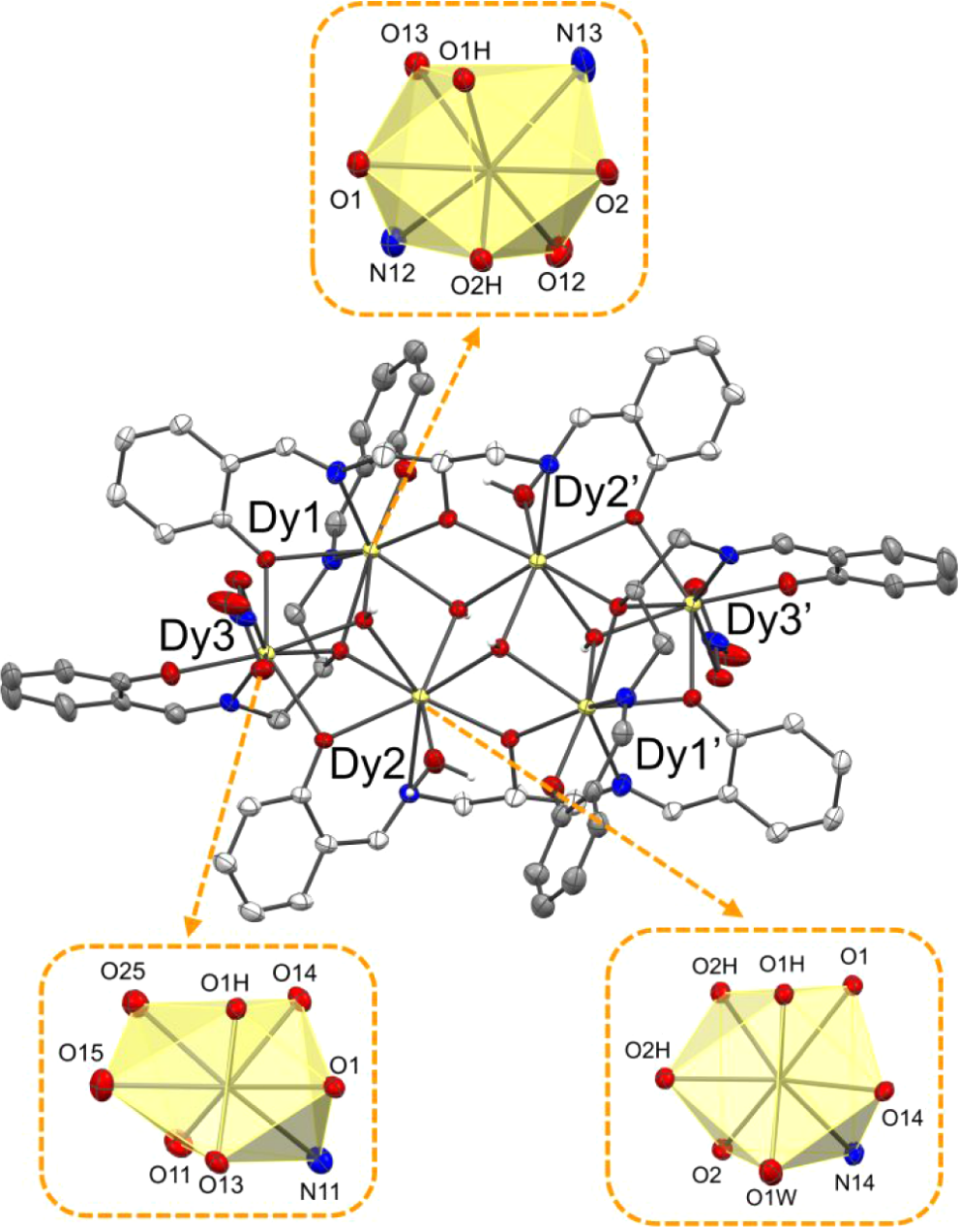
Molecular structure of the hexanuclear complex [Dy_6_(L^H^)_4_(OH)_4_(NO_3_)_2_(H_2_O)_2_]·(CH_3_COCH_3_)_1.66_ (**3**). Solvent of crystallization and the hydrogen atoms (except for the hydroxide groups O1H, O2H, and the water molecules O1W) were removed for clarity. Thermal ellipsoids were depicted at the 30% probability level. The distorted bicapped trigonal prismatic geometry of Dy1, Dy2, and Dy3 is highlighted. Symmetry code′: (1 – *x*, 1 – *y*, 1 – *z*). Dy (pale yellow), N (blue), O (red), C (gray and light gray), H (white).

#### 2.1.4 Structures of L^o‑tBu^ Complexes

As reported above, the structures of the complexes of H_3_L^o‑tBu^ differ from those obtained by the other ligands due to the high steric hindrance of the *t*-Bu group of the ligand. This makes this ligand more prone to forming structures with a lower nuclearity. The higher steric hindrance prevents the ligand from adopting some conformation that would lead to crowding on the metal complex (i.e., bent conformation), which is also reflected in the lower μ value for the ligands in the complexes (Table S6). This is also evident comparing the spatial disposition of the dinuclear H_3_L^o‑tBu^ with the trinuclear H_3_L^H^/H_3_L^p‑OMe^ one, where the hypothetical position of the *t*-Bu group in a trinuclear complex would be incompatible with the presence of other ligands (Figures S23 and S24). Additionally, the [M_3_(μ_3_-OH)­(μ_3_-OR)­(μ_2_-O)_3_] subunit is not present in the complexes of H_3_L^o‑tBu^ but is replaced by either a [M_2_(μ_2_-O)_2_] subunit in the structures containing Dy (complexes **5**, **6**, and **8**), or a [M_3_(μ_3_-OH)­(μ_2_-O)_3_] subunit, similar to the secondary subunit described earlier, in the Nd complex (**7**). Dinuclear compounds **5** and **6** crystallize in the *P*-1 and *P*2_1_/*c* space groups, respectively. The complex entity of the two structures is the same, with the differences limited to the ancillary ligands completing the metal coordination (ethanol for compound **5** and THF for compound **6**; Figures S18 and S19) deriving from the crystallization in different solvents. Therefore, only the structure of compound **5** is described here. The dinuclear complex consists of two Dy^3+^ ions coordinated by two L^o‑tBu^ ligand and two coordinated ethanol molecules (Figures 6a and S18). The dysprosium atoms are eptacoordinated, with Dy1 exhibiting a capped trigonal prismatic geometry (N_2_O_5_ with N11 as capping position), while Dy2 displays a capped octahedral geometry (N_2_O_5_ with O2 as capping position). The structure presents the [Dy_2_(μ_2_-OR)_2_] subunit held together by the central alkoxides of the two ligands. The conformation of the ligand is semibent, with the two tridentate moieties chelating two different metals and sharing the alkoxide as bridging atom. One ethanol molecule is present in the second coordination sphere of the complex and it forms an hydrogen bond chain with the ethanol molecule coordinated to Dy2 (O4S–H···O7S) and with the phenolate of the phenolate oxygen of a ligand bound to Dy1 (O7S–H···O13) (O–O distances of 2.69(1)­and 2.84­(1) Å, respectively). On the other side of the complex, an intramolecular hydrogen bond is present between the ethanol molecule coordinated to Dy1 and the phenolic oxygen atom (O12) coordinated to Dy2 (2.89(1) Å). As anticipated in the introduction, the purpose of the work was to identify possible condition leading to the separation of Dy and Nd from their mixtures. Interestingly, we could isolate some crystals of a Nd/Dy heteronuclear complex in a mixed metal mixture with a H_3_L^o‑tBu^ ligand. The crystals were presumably not representative of the overall solution speciation of the complexes, but it was instructive to have experimental evidence for the formation of a mixed-metal species. Compound **7** crystallizes in a monoclinic crystal system and space group *P*2_1_/*c*. The complex entity is extremely similar to the one found in compound **5**, forming a dinuclear complex, but with a major difference coming from the synthetic procedure (Figure 6b). Compound **5** was obtained by reaction of H_3_L^o‑tBu^ and NEt_3_ with Dy­(NO_3_)_3_·5­(H_2_O), whereas dinuclear compound **7** was obtained by reaction of H_3_L^o‑tBu^ and NEt_3_ with a 1:1 mixture of Dy­(NO_3_)_3_·5­(H_2_O) and Nd­(NO_3_)_3_·6­(H_2_O). From ESEM-EDS analysis on the same crystal used for X-ray diffraction, the Nd:Dy ratio was confirmed to be 1:1 (Figure 6c,d). This meant that two main possibilities for the refinement of the metal positions inside the two metal sites in the asymmetric unit cell were present: (i) Nd always present in one site and Dy always present in the other site; (ii) Nd and Dy are disordered over the two metal sites with overall unitary occupancies for each. From the refinement parameters obtained by the two possible situations, the best solution was obtained by the refinement of Dy and Nd disordered over the same positions with free variables, leading to site occupancy factors of 0.69 and 0.31 for the first site and 0.31 and 0.69 for the second site for Dy and Nd respectively (Table S13 and Figure S21). The disorder in the metals also affects the ancillary ethanol molecules coordinated to them, thanks to the different preferences in the coordination number of the two lanthanide ions. In fact, in the first metal site (0.69 occupancy of Dy), only one ethanol molecule is coordinated to the metal (Figure S20a). However, in the second position (0.69 occupancy of Nd) the ethanol residues were found disordered over two positions with an occupancy of 0.69 (matching that of Nd) and in one position with an occupancy of 0.31 (matching that of Dy) (Figure S20b). The isolation of this heteronuclear structure is evidence of the possibility of Nd and Dy to substitute for each other in the polynuclear complexes giving rise to multiple heteronuclear structures with the same overall chemical structure.

**Figure 6:**
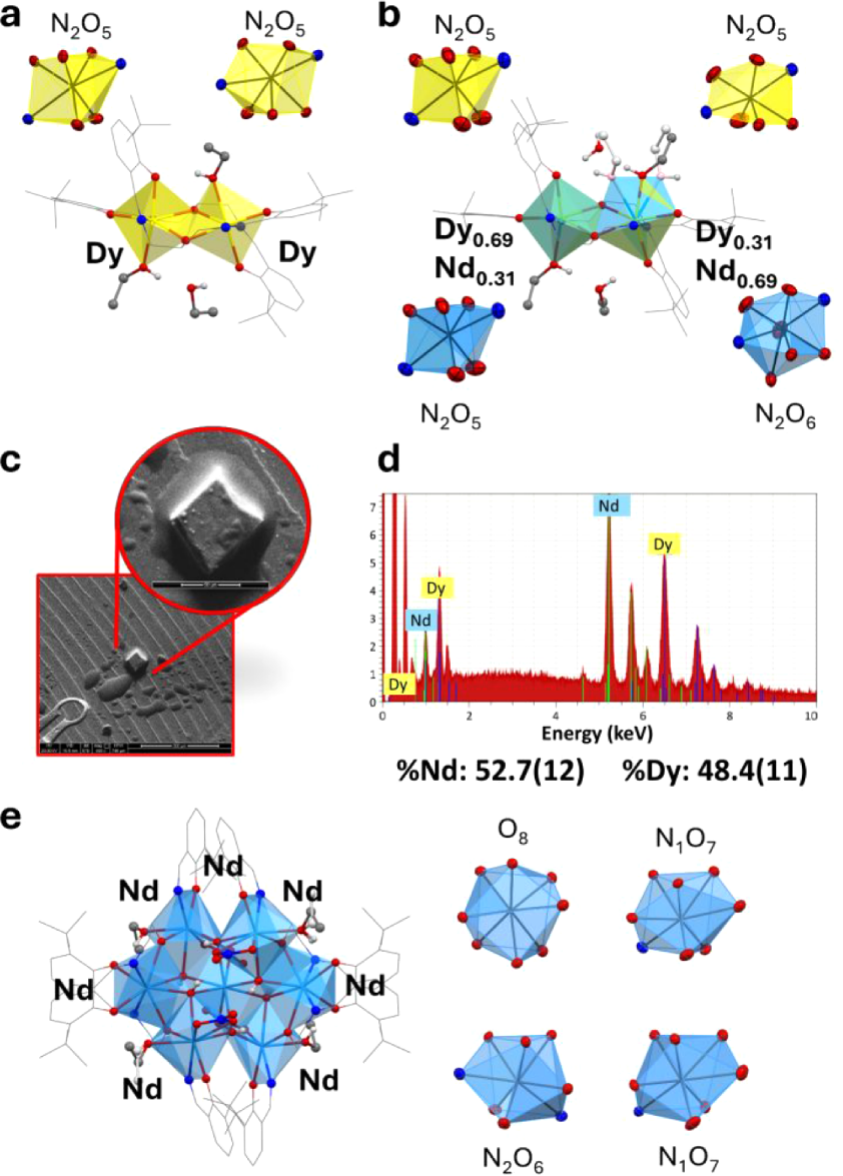
a) Molecular structure of complex **5** highlighting the coordination polyhedra of the metals. b) Molecular structure of the heteronuclear complex **7** highlighting the coordination polyhedra of the metals. c) ESEM images of the crystals of compound **7** after X-ray diffraction analysis at 400× and 1600× magnification. d) EDS spectra of the crystal of compound **7** highlighting the emissions of Nd and Dy. e) Molecular structure of complex **8** highlighting the coordination polyhedra of the metals. Hydrogen atoms and noncoordinated solvent molecules were omitted for clarity.

Heptanuclear Nd complex **8** crystallizes in the monoclinic *C*2/*c* space group. The complex consists of a [Nd_7_] metal cluster held together by six μ_3_-OH hydroxide ions and four triply deprotonated L^o‑tBu^ ligands (Figures 6e and S22). Four metals are present in the asymmetric unit with Nd1 lying on a 2-fold symmetry axis. The spatial disposition of the ligands is different from that of the hexanuclear complexes as they are located only on the outside of the metal cluster and do not interact with the central Nd1 ion. In fact, the coordination of this ion is saturated by the six hydroxides and two bridging nitrates (bidentate chelating Nd4 and Nd4′) with an overall square antiprismatic O_8_ coordination. All metal ions are octacoordinated, and apart from Nd1, the other ions present a bicapped trigonal prismatic coordination (N_1_O_7_, N_2_O_6_, and N_1_O_7_ for Nd2, Nd3, and Nd4, respectively) with the capping positions represented by the O1 and O3 for Nd2, N14, and O1 for Nd3, and O2 and O25 for Nd4. All four ligands are in a linear conformation with the two tridentate moieties chelating two different metal sites and with the central alkoxides bridging between the two. The first ligand residue binds Nd2 and Nd3, while the second one binds Nd3 and Nd4 with no ligands bridging between the two symmetry-related halves of the complex. The coordination environment of Nd2 and Nd4 is completed by the presence of an ethanol molecule in the first coordination sphere of each. The charge balance is provided by the presence of a total of four chelating nitrates and an Et_3_NH^+^ ion (coming from the base used during complex synthesis) that was heavily disordered inside a large solvent-accessible cavity in the structure. Therefore, the electronic density was modeled with the MASK routine implemented in Olex2 and the electron count matched with the presence of an Et_3_NH^+^ ion and 8 ethanol molecules for each complex entity. In the structure, three trinuclear [Nd_3_(μ_3_-OH)­(μ_2_-O)_2–3_] subunits can be observed. The primary and secondary subunits are similar to the secondary subunits observed for the other polynuclear compounds, with a [Nd_3_(μ_3_-OH)­(μ_2_-O)_3_] substructure, a close-to-isosceles triangular geometry, and average Nd–Nd distances of 3.9(1) and 3.93(3) Å, respectively. In the tertiary subunit, the structure is different from the others, with a [Nd_3_(μ_3_-OH)­(μ_2_-O)_2_] substructure, a longer mean Nd–Nd distance (4.1(1) Å), and a heavily distorted structure, making it almost a rectangular triangle shape, with two Nd ions and the central μ_3_-OH as the base of the triangle (Nd–O–Nd angle of 149.6°). The central μ_3_-OH and two μ_2_-O residues are situated on the underside of the plane formed by the three metal ions of the subunit, as opposed to the other subunits, where they reside on opposite sides of the [Nd_3_] mean plane. Overall, three subunits are repeated two times each in a circular manner, with the primary and secondary [Nd_3_] subunits more or less planar (angle of 11.3°), while the tertiary subunit is heavily tilted with respect to the secondary subunit (42.1°) and the symmetry generated primary unit (61.0°).

### 2.2 Dy and Nd Separation Experiments

The ligands H_3_L^H^, H_3_L^p‑OMe^, and H_3_L^o‑tBu^ used in this study exhibit good solubility in most solvents. Despite the presence of the imine functional group, their stability was assessed under various conditions to ensure their suitability as separation agents. Stability tests were performed on H_3_L^p‑OMe^ by dissolving the ligand in distilled water, 50 mM HEPES, and 50 mM oxalic acid, respectively. The results indicated gradual degradation in water and HEPES, whereas degradation in oxalic acid was almost immediate (Figure S26). From a screening of various organic solvents, ethanol was selected as the most appropriate for the separation experiments because all ligands showed high solubility, and the stock solutions remained stable for at least a week under nondry conditions (Figure S25). Notably, the presence of adventitious water in ethanol did not induce ligand degradation over time. Ethanol is classified as a green solvent due to its low environmental impact, affordability, low volatility, and especially its potential replacement with bioethanol, which is primarily derived from renewable sources such as biomass.^73^ Hence, a systematic series of experiments was conducted to separate Nd^3+^ and Dy^3+^, with each experiment performed in duplicate. This study was based on the preliminary observation of the solubility differences exhibited by the Nd^3+^ and Dy^3+^ complexes during the preparation experiments (Figure 7). Specifically, in the presence of H_3_L^H^ and H_3_L^p‑OMe^, neodymium complexes exhibited a lower solubility with respect to dysprosium complexes. In the presence of H_3_L^o‑tBu^, the dysprosium complex was the most insoluble. Prompted by these observations, preliminary experiments in ethanol were conducted to evaluate the ability of the ligands to react with nitrate salts of Nd^3+^ and Dy^3+^ thus forming complexes with pronounced solubility differences. The metal nitrate salts (*C*_Nd_ = *C*_Dy_ = 10 mM) were separately reacted with the ligands (*C*_L_ = 10 mM) in the presence of 3 equiv of triethylamine as a base, relative to the ligand, for 2 h. In order to maximize the solubility difference between Nd^3+^ and Dy^3+^ complexes, the temperature for the reactions and for the solubility experiments was set at 55 °C. Indeed, at room temperature, the complexes started to precipitate out, thus limiting the potential separation between the two metals. The percentages of Nd^3+^ and Dy^3+^ in the solid phase and the supernatant were measured through Inductively Coupled Plasma-Optical Emission Spectroscopy (ICP-OES) on an appropriately digested fraction of the precipitate. The solid samples were collected via filtration at the reaction temperature. Under the investigated conditions in ethanol, with the H_3_L^H^ ligand, Nd^3+^ could be completely recovered in the precipitate, whereas all of Dy^3+^ remained in the solution phase. On the other hand, with H_3_L^p‑OMe^, the Nd^3+^ was found in both solid and solution phases, whereas Dy^3+^ remained completely in solution. Interestingly, in the presence of the bulkier H_3_L^o‑tBu^ an inversion of the solubility was observed, Nd^3+^ was almost quantitatively found in the solution phase, while Dy^3+^ was found in the precipitate. Hence, the three ligands H_3_L^H^, H_3_L^p‑OMe^, and H_3_L^o‑tBu^ demonstrated potential for the selective separation of these metals from a multi-REE mixture under the tested or similar conditions.

**Figure 7:**
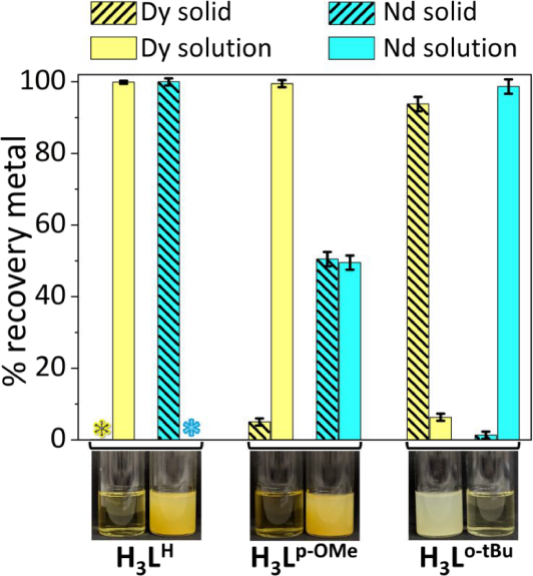
Distribution of Nd^3+^ and Dy^3+^ in the solid and solution phases in the presence of H_3_L^H^, H_3_L^p‑OMe^, and H_3_L^o‑tBu^ in ethanol, as measured by ICP-OES. *C*_M_ = 10 mM, *C*_L_ = 10 mM, reaction time 2 h, 55 °C. The asterisks denote the absence of Dy in the solid phase and Nd in solution, respectively, as their concentrations were below the instrument’s Limit of Detection (LOD).

In the separation study, a mixture of neodymium and dysprosium nitrates in ethanol was reacted with one of the three ligands in two distinct molar ratios in ethanol, H_3_L^R^:Nd:Dy 1.05:0.5:0.5 (*C*_Nd_ = *C*_Dy_ = 10 mM) and 1.05:0.8:0.2 (*C*_Nd_ = 24 mM, *C*_Dy_ = 6 mM for H_3_L^p‑OMe^; *C*_Nd_ = 14.5 mM, *C*_Dy_ = 3.5 mM for H_3_L^H^ and H_3_L^o‑tBu^). 3 equiv of TEA relative to the ligand were used for all of the experiments. The H_3_L^R^:Nd:Dy ratio of 1.05:0.8:0.2 (Nd:Dy of 4:1) was used to provide insight into the applicability of the separation system for real PMs samples, in which the Nd:Dy ratio can be greater than 9:1. All the separation experiments led to the formation of a solid precipitate, which was separated from the solution by filtration, and both phases were analyzed using ICP-OES to assess the metals’ distribution. In Table 2 all Separation (*S*) and Enrichment Factors (EF) are reported for the tested conditions. For ligand H_3_L^H^, an almost complete precipitation of the Nd complex was observed after 2 h of reaction time, at which point dysprosium-containing species were also present in the solid. In the solution phase, at 2 h, there was an enrichment of dysprosium-containing species as most of the neodymium precipitated, Figure 8a. The separation factors (*S*_Nd/Dy_) were 7.5 (±0.4) and 12.0 (±2.0) after 30 min and 2 h, respectively. When considering the Nd:Dy 4:1 stoichiometric ratio, in the presence of H_3_L^H^, *S*_Nd/Dy_ was 16 (±4) after 2 h (Figures 8b and S27). As far as H_3_L^p‑OMe^ is concerned, when the ratio between the metals was 1:1, no separation ability was observed, since Nd^3+^ and Dy^3+^ tended to distribute equally between both phases, and *S*_Nd/Dy_ values were less than 2 (Figure 8c). However, with the Nd:Dy 4:1 ratio, a presumably greater distribution of Nd^3+^ in the solid phase could be observed, leading to a separation factor (*S*_Dy/Nd_) of 16 (±7), Figure 8d. Consistent with the preliminary solubility experiments, H_3_L^o‑tBu^ promoted an enrichment of Nd^3+^ in the solution phase and an enrichment of Dy^3+^ in the solid phase, which was the opposite scenario of what was observed for H_3_L^H^ and to a lesser extent for H_3_L^p‑OMe^. Another notable difference with the two other ligands is that H_3_L^o‑tBu^ induces a significantly faster precipitation kinetic since most of the Dy^3+^ species precipitate from the reaction environment in the first minutes after the reagent is added to the mixture. In fact, with increasing reaction time and up to 30 min, a progressive enrichment of Nd^3+^ in the solid phase was observed, together with an additional minor fraction of Dy^3+^ species. *S*_Dy/Nd_ values were 20 (±4) and 9 (±2) after 10 and 30 min of reaction time, respectively (Figures 8e and S28).

**Table 2:** Separation Coefficients and Enrichment Factors for the Intra-Series Separations of Neodymium and Dysprosium (Nd:Dy, 1:1; *C*_M_ = 10 mm) (Nd:Dy 4:1; *C*_Nd_ = 24 mm; *C*_Dy_ = 6 mm for ^–OMe^; *C*_Nd_ = 14.5 mm; *C*_Dy_ = 3.5 mm for H_3_L^H‑tBu^) under the Indicated Conditions^a^,^b^

Ligand	Time	Nd:Dy	EF_M1,solid_	EF_M2,supernatant_	*S*_M1/M2_
H_3_L^H^	30 min	1:1	1.88 (±0.04)	4.0 (±0.2)	7.5 (±0.4)
	2 h	1:1	1.40 (±0.02)	9 (±1)	12 (±2)
	2 h	4:1	11 (±2)^c^	1.5 (±0.3)^c^	16 (±4)
H_3_L^p‑OMe^	30 min	1:1	1.17 (±0.05)	1.07 (±0.01)	1.25 (±0.05)
	2 h	1:1	1.26 (±0.01)	1.11 (±0.02)	1.40 (±0.03)
	2 h	4:1	6 (±2)^c^	2.4 (±0.5)^c^	16 (±7)
H_3_L^o‑tBu^	10 min	1:1	6 (±1)	3.1 (±0.1)	20 (±4)
	30 min	1:1	2.5 (±0.2)	3.7 (±0.5)	9 (±2)
	2 h	4:1	0.5 (±0.1)^c^	29 (±9)^c^	13 (±5)

a The results are reported as the mean ± standard deviation , rounded at one significant digit (replicate).

b EF_M1_ = *n*_M1_/*n*_M2_, where *n* is the number of moles; *S*_M1/M2_ = EF_M1,solid_ × EF_M2,supernatant_. M1 = Nd and M2 = Dy for H_3_L^H^, H_3_L^p‑OMe^. M1 = Dy and M2 = Nd for H_3_L^o‑tBu^.

c For a Nd:Dy ratio of 4:1 and in the case of no separation, the values of EF_Nd,phase1_ and EF_Dy,phase2_ would be 4 and 0.25, respectively.

**Figure 8:**
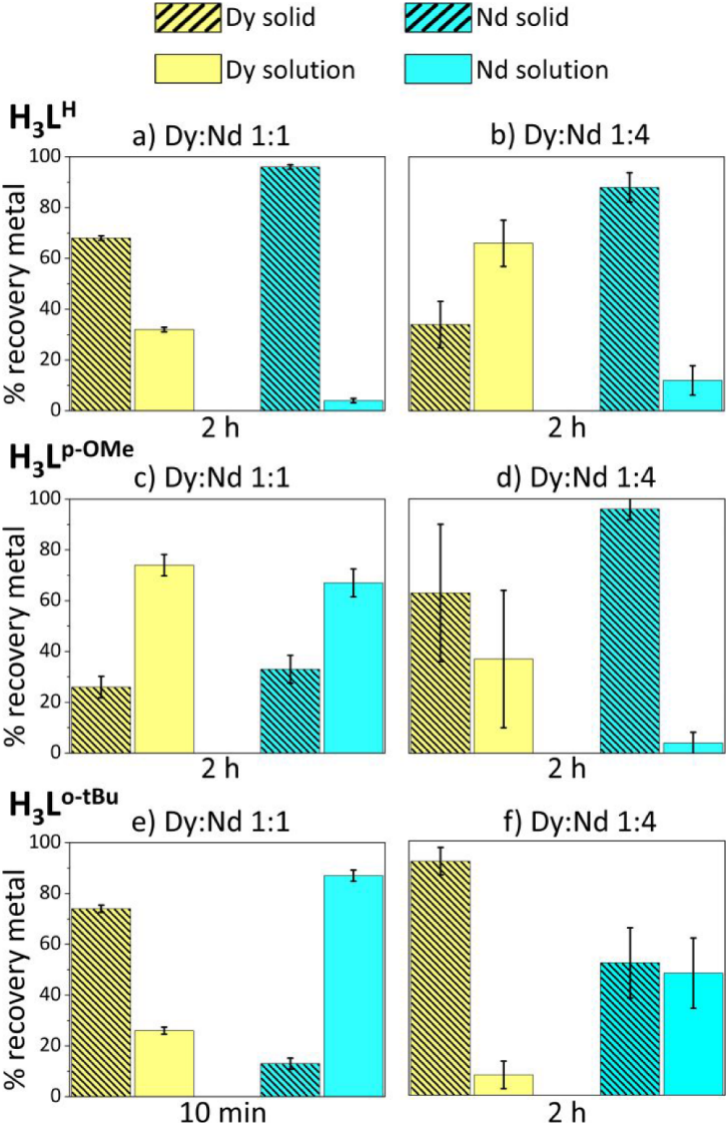
ICP-OES results. Weight percent of Nd and Dy recovered from the solution and solid phases for two different stoichiometric ratios in absolute ethanol after mixing Nd^3+^, Dy^3+^, H_3_L^H^, H_3_L^p‑OMe^, or H_3_L^o‑tBu^ with triethylamine in 0.5:0.5:1.05:3.15 and 0.8:0.2:1.05:3.15 stoichiometric ratios, respectively. H_3_L^H^ and H_3_L^p‑OMe^ reacted for 2 h, while H_3_L^o‑tBu^ reacted for 10 min and 2 h. The metal cations were added as nitrate salts.

As a general observation, it is important to highlight a significant difference with the precipitation studies performed on the isolated systems and, in particular, with H_3_L^H^ and H_3_L^o‑tBu^, which were characterized by a markedly different solubility behavior for Dy^3+^ and Nd^3+^, see Figure 7. When both metals were simultaneously present in the feed solution, it was not possible to identify, in the indicated experimental setup, a condition in which one of the two phases exclusively contained one metal. The reason for this behavior may depend on a coprecipitation phenomenon, where the precipitation of one species comprising one of the two metals triggers the precipitation of the second homonuclear metal species. In this case we could assume that both systems are at the supersaturation regime and the solid compounds precipitate following different precipitation kinetics. In this respect, it was shown that kinetic aspects are important in the crystallization of oxalate salts of some lanthanide cations, with early (light) lanthanides having a slower precipitation with respect to late (heavy) lanthanides.^74^ Similarly, it was shown that hydrate lanthanide chlorides in the presence of molten boric acid gave different structural types, characterized by a distinct solubility and precipitation kinetics.^75^ The formation of different structural types for the lanthanide series was also shown in the presence of 1,10-phenanthroline-2,9-dicarboxylic acid and *N*,*N*′-dimethylformamide. A separation factor of approximately 26 was found for the La/Lu 1/1 mixture after 3 days.^76^ An additional reason for the occurrence of the two metals in the various precipitates may be due to the polynuclear nature of the complexes. In fact, a likely scenario is the formation of mixed metal polynuclear species (like the one observed for compound **7**) during the process of complex formation in solution, on the account of the small difference in ionic radii between Nd^3+^ (1.11 Å) and Dy^3+^ (1.03 Å).^77,48^ To further investigate this aspect, electrospray ionization mass spectrometry (ESI-MS) was employed to assess the presence of heteronuclear species during the separation of neodymium and dysprosium with ligands H_3_L^H^ and H_3_L^p‑OMe^. Spectra of single metal complexes were recorded and compared with those obtained from the redissolved solids and supernatant phases of the corresponding separation experiments, prepared with a 1:1 Nd:Dy molar ratio. The samples were analyzed after reaction for 2 h (upon dissolution of the precipitate or by direct analysis of the supernatant after dilution), under the same conditions used for the separation experiments. For the H_3_L^H^ ligand, the analysis on the crude single-metal reaction mixtures (Figure 9a,b) revealed that neodymium predominantly formed species with nuclearities equal or greater than [Nd_4_HL^H^L^H^_3_]^+^ (1757 *m*/*z*), whereas dysprosium mainly formed three species, [Dy_2_HL^H^L^H^]^+^ (917 *m*/*z*), [Dy_3_HL^H^L^H^_2_]^+^ (1375 *m*/*z*) and [Dy_4_HL^H^L^H^_3_H_2_O]^+^ (1850 *m*/*z*). Comparison between the spectra from the pure single-metal systems and those from the supernatant and solid phases of the mixed-metals experiments (Figure 9c,d, respectively) showed that all peaks corresponding to the pure Dy species were retained in the supernatant, in accordance with the solubility reported in Figure 7. Moreover, additional peaks in the separation experiment samples were observed and attributed to the formation of mixed [Dy_*x*_Nd_*y*_L^H^_*x*+_ *_y_*]^+^ complexes. The attribution was supported by the characteristic isotopic distributions of the two metals, shown in Figures S29 and S30. A similar trend was observed with the H_3_L^p‑OMe^ ligand, reported in Figures S31 and S32. In the single-metal systems (Figure S31a,b), neodymium tended to form species with nuclearities higher than [Nd_5_L^p‑OMe^_4_(NO_3_)_2_]^+^ (2266 *m*/*z*), while dysprosium predominantly formed 3:3 and 4:4 metal-to-ligand species (1555 and 2092 *m*/*z*, respectively). In the presence of both metals (Figure S31c,d), additional peaks were observed and attributed to the formation of mixed-metal species. Thus, the presence of species containing both Nd^3+^ and Dy^3+^ could provide a possible explanation to the ICP-OES results in Figure 8a,c, highlighting the separation criticalities. In particular, the detection of dysprosium in the solid phase and of neodymium in the supernatant could be attributed to the presence of insoluble mixed-metal species, typically enriched in neodymium and dysprosium, respectively. However, the attribution of the peaks relative to mixed-metal species was complicated by the small mass difference between Nd and Dy (approximately 18 u), which is equivalent to that of a water molecule. For this reason, additional experiments were conducted using yttrium instead of dysprosium. Yttrium was selected due to its similar ionic radius to that of dysprosium (Y^3+^= 1.02 Å and Dy^3+^= 1.03 Å for a coordination number of eight), thus assuming a similar chemical behavior of the two metals, as well as its difference in atomic mass (55 u) and isotopic distribution from Nd, providing a way to distinguish between homonuclear and heteronuclear species. As reported in Figures 9 and S38 in the single-metal experiments (Figures 9e,f and S31a,b), yttrium tended to behave in a similar way as dysprosium, forming species such as [Y_3_HL^H^L^H^_2_]^+^ (1153 *m*/*z*), [Y_4_HL^H^L^H^_3_H_2_O]^+^ (1555 *m*/*z*), [Y_4_HL^p‑OMe^L^p‑OMe^_3_H_2_O]^+^ (1333 *m*/*z*), [Y_3_HL^p‑OMe^L^p‑OMe^_2_(OH)­(NO_3_)­Na]^+^ (1437 *m*/*z*) and [Y_4_HL^p‑OMe^L^p‑OMe^_3_H_2_O]^+^ (1795 *m*/*z*). In the separation experiments (Figures 9g,h and S31c,d), the peaks corresponding to the pure Y species were retained in the supernatant and solid spectra as well as additional peaks. However, in this case, the attribution of heteronuclear species was simplified by the pronounced change in isotopic distribution between the pure Y and the mixed Y:Nd species (Figures S36, S37, S39, and S40). In the case of the H_3_L^o‑tBu^ ligand, analysis of the crude single-metal reaction mixtures revealed that dysprosium formed the [DyL^o‑tBu^_2_(H_2_O)_4_Na_2_]^−^ (2:1) and [Dy_2_L^o‑tBu^_2_(NO_3_)]^−^ (2:2) species, observed at 1096 and 1201 *m*/*z*, respectively (Figure S33). For neodymium, a single species [NdL^o‑tBu^_2_(H_2_O)_4_Na_2_]^−^ (2:1) was detected at 1076 *m*/*z* (Figure S34). Unlike the behavior observed with H_3_L^H^ and H_3_L^p‑OMe^, no heteronuclear species were detected in the mixed-metal experiments with H_3_L^o‑tBu^. The absence of mixed-metal complexes was confirmed by the isotopic distribution patterns (Figure S35), which were consistent with the exclusive presence of the homonuclear species. This conclusion was further supported by experiments performed with yttrium. In the presence of neodymium, a [Y_2_HL^o‑tBu^L^o‑tBu^Na]^+^ (2:2) species was detected at 1015 *m*/*z* with no evidence of heteronuclear Y–Nd complexes. The isotopic pattern observed matched that expected for a single-metal species (Figure S41), providing additional evidence for the lack of a mixed-metal complex formation with this ligand system. This observation is in apparent contrast with the single-crystal X-ray structure of mixed complex **7**. It has, however, to be borne in mind that the crystallographic analysis was performed on a sample that was obtained after several days of crystallization for the parent mixed-metal solution and would not necessarily reflect the ESI-MS speciation in solution. Another important issue to consider when interpreting the ESI-MS data is related to the insolubility of the solid obtained after the precipitation in the solvents used for the ESI-MS experiments (MeOH or ACN), preventing its detection.

**Figure 9:**
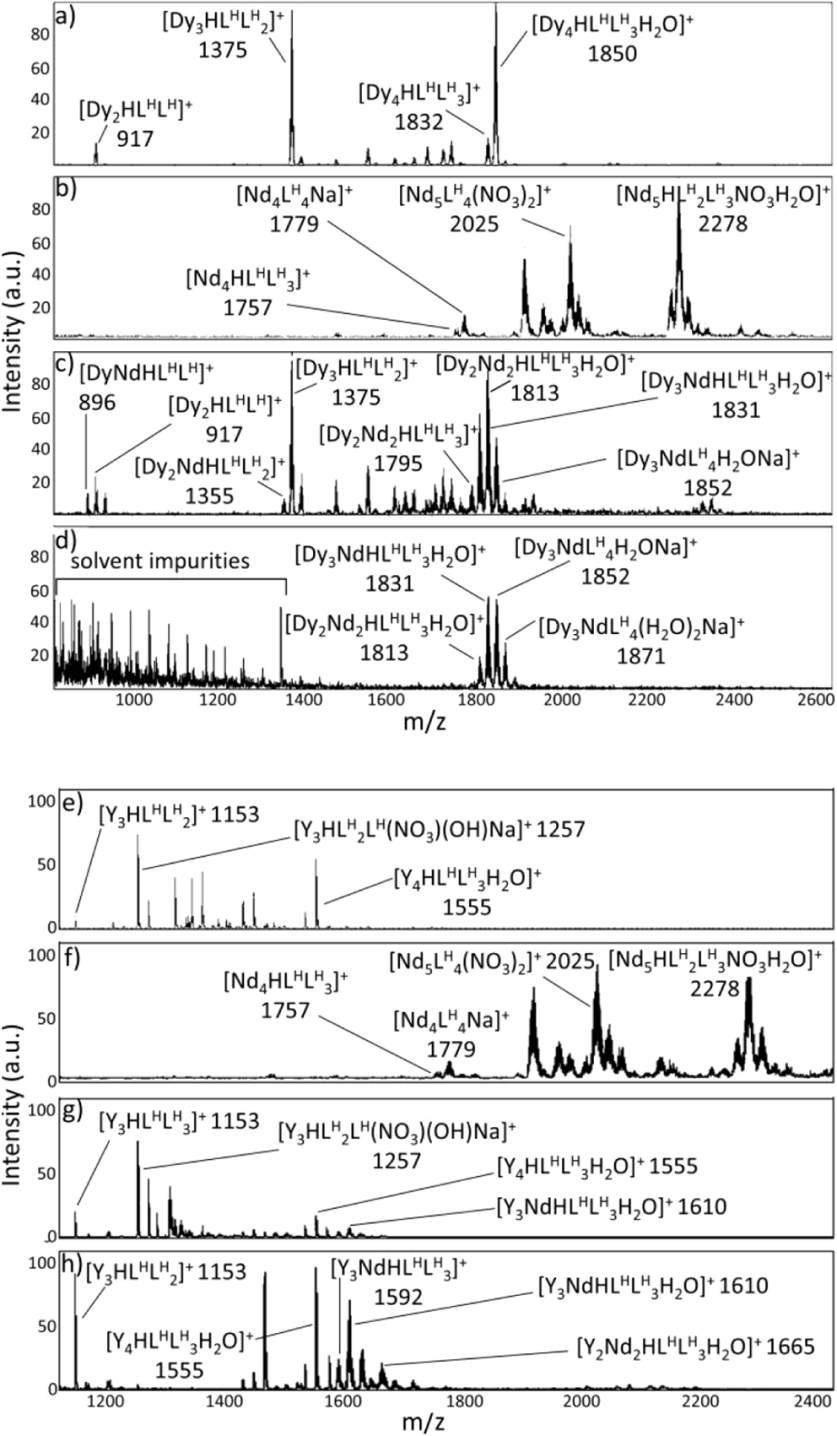
Above panel, ESI-MS (+) spectra of the crude reaction mixture of H_3_L^H^ with dysprosium (a), neodymium (b), together with the liquid (c) and solid (d) phases obtained from the separation of neodymium and dysprosium (H_3_L^H^:Nd:Dy 1.05:0.5:0.5, *C*_Dy_ = *C*_Nd_ = 10 mM). Below panel, ESI-MS (+) spectra of the crude reaction mixture of H_3_L^H^ with yttrium (e), neodymium (f), together with the liquid (g) and solid (h) phases obtained from the separation of yttrium and neodymium (H_3_L^H^:Nd:Y 1.05:0.5:0.5, *C*_Y_ = *C*_Nd_ = 10 mM).

## 3 Conclusions

Recovering and recycling rare earth elements from end-of-life products reduces reliance on environmentally damaging mining processes, helps support a more sustainable and secure supply chain, and ultimately contributes to the circular economy. In this work we investigated the possible separation of neodymium and dysprosium on the account of the different solubility exhibited by complexes formed with variously functionalized N_2_O_3_ donor ligands (the simplest of which is *N*,*N*-bis­(salicylidene)-1,3-diamino-2-propanol, H_3_L^H^). According to the coordination preferences of lanthanide cations, the potential pentadentate nature of the ligands is not sufficient to satisfy the metal requirements, as the Ln^3+^ cations usually have coordination numbers that are ≥7.^65,78^ Hence, the formation of complexes having a stoichiometry different from that of 1:1 M:H_3_L^R^ was a plausible scenario. According to the structural characterization performed on the Ln^3+^ complexes with H_3_L^H^, H_3_L^p‑OMe^, and H_3_L^o‑tBu^ ligands the following stoichiometric ratios between metal and ligands were observed: 2:2 (H_3_L^o‑tBu^), 3:3 (H_3_L^p‑OMe^), 4:3 (H_3_L^H^), 6:4 (H_3_L^H^ and H_3_L^p‑OMe^), and 7:4 (H_3_L^o‑tBu^). Interestingly, H_3_L^o‑tBu^ gave a complex with a 2:2 stoichiometry in the presence of Dy^3+^ and a complex with a 7:4 metals-to-ligand with Nd^3+^. Solubility studies combined with the analysis of crystalline structures provided valuable insights for selecting optimal conditions to attempt separation experiments. In the presence of both metals, the experiments yielded less distinct results compared to the precipitation tests performed on single-metal systems. Notably, appreciable quantities of the metal anticipated to be absent, based on preliminary single-metal assessments, were detected in both the solid and liquid phases.

Electrospray ionization mass spectrometry (ESI-MS) analyses performed in methanol on both the precipitates and supernatants from the separation experiments revealed the formation of both homonuclear and heteronuclear complexes with variable compositions in both the solid and the liquid phases. With the H_3_L^H^ and H_3_L^p‑OMe^ ligands, the tendency of neodymium to form higher-nuclearity species seems to promote its precipitation; however, the concurrent formation of mixed-metal complexes can hinder the efficiency of the separation process. This behavior was suggested by mass spectrometry analysis, specifically through the observed isotopic distributions of [Dy_*x*_Nd_*y*_L^H^_*x*+_ *_y_*]^+^ species and further supported by the isolation of the crystal structure of compound **7** ([NdDy­(L^o‑tBu^)_2_(EtOH)_2.69_]­EtOH·(H_2_O)_0.2_).

The most favorable separation factors (*S*) were achieved using the H_3_L^H^ ligand (*S* = 12 (±2) for the Nd:Dy:L 1:1:2 system and *S* = 16 (±4) for the Nd:Dy:L 1.6:0.4:2 system after 2 h) and with the H_3_L^o‑tBu^ ligand yielding *S* of 20 (±4) for the Nd:Dy:L 1:1:2 system after 10 min and *S* of 13 (±5) for the Nd:Dy:L 1.6:0.4:2 system after 2 h. These findings indicate that the selective precipitation of metals upon complexation is facilitated by ligands that are less prone to generating heterometallic polynuclear species. Consequently, the rational design of polydentate ligands capable of selectively meeting the coordination requirements of one metal over the others targeted for separation appears to be of critical importance.

## 4 Experimental Section

### 4.1 Materials and Methods

No uncommon hazards were noted. All chemicals were purchased from Merck and Alfa Aesar and were used without further purification. NMR experiments were performed on a Bruker Avance 400 MHz instrument at 298 K and chemical shifts are reported in parts per million relative to tetramethylsilane. Infrared spectra (IR) were recorded by a PerkinElmer Spectrum Two spectrometer in the 4000–400 cm^–1^ interval. ESI-MS analyses of ligands were carried out by using a Waters Acquity Ultra Performance LC with a Waters Acquity SQ Detector and with an ESI interface. The solutions were analyzed in negative ionization mode by direct perfusion in ESI-MS interface; injection flow rate = 0.200 mL min^–1^. ESI-MS analyses of complexes were carried out by using an Agilent HPLC 1260 Infinity II, Agilent InfinityLab LC/MSD XT detector with an Agilent Jet Stream source. The solutions were analyzed in both positive and negative ionization modes by direct perfusion in ESI-MS interface; injection flow rate= 0.350 mL/min. Elemental analyses (CHN) were performed on Thermo Fischer Scientific FlashSmart CHNS analyzer (sample mass: 2–3 mg). The UV–vis spectroscopic stability studies of the ligand were performed using a Photodiode Array Lambda 465 spectrophotometer equipped with a Peltier thermostat and 1 cm path length quartz cuvettes.

### 4.2 Single-Crystal X-ray Data Collection

Single crystal data of **2**−**8** were collected with a Bruker D8 Venture diffractometer equipped with a Photon III detector (Mo Kα: λ = 0.71073 Å) or a Bruker D8 Venture diffractometer equipped with a Photon II area detector diffractometer (Cu Kα: λ = 1.54184 Å). Complete data sets were obtained by merging several series of exposure frames collected at 200 K. Data reductions were performed with APEX5. Absorption correction was applied with the program SADABS.^79^ Data for **1** were collected at 100 K at the XRD2 beamline of the Elettra Synchrotron­[CIT], Trieste (Italy) using a monochromatic wavelength of 0.62 Ang.^80^ The structures were solved by intrinsic phasing with ShelxT^81^ and refined on F^2^ with full-matrix least-squares (ShelxL)^82^ using the Olex2 software package (version 1.5).^83^ All non-hydrogen atoms were refined with anisotropic thermal parameters except for compound **1** (two DMF molecules and the unbound nitrate were refined with isotropic displacement parameters), compound **2** (four DMF molecules, the unbound nitrate and one water molecule were refined with isotropic displacement parameters) and compound **3** (four DMF molecules, the unbound nitrate and one water molecule were refined with isotropic displacement parameters). Hydrogen atoms were placed in calculated positions and refined with a riding model. In the structure of compound **1** two DMF molecules were refined with site occupancies of 0.5, and the unbound nitrate ion that was found disordered over two positions was refined with site occupancy factors of 0.82 and 0.18, respectively. In the structure of compound **2** one nitrate ion was found disordered over two sites, which were refined with site occupancy factors of 0.7 and 0.3, respectively. The central OH of one of the ligands was found disordered over two positions that were refined with site occupancy factors of 0.56 and 0.44, respectively. One DMF molecule was found disordered over two sites that were refined with site occupancy factors of 0.54 and 0.46, respectively. Three DMF molecules were refined with site occupancy factors of 0.5, and one DMF molecule was refined with site occupancy factors of 0.25. In the structure of compound **3** one acetone molecule was refined with a site occupancy factor of 0.66 and the other was found disordered over two sites, which were refined with site occupancy factors of 0.73 and 0.26, respectively. In the structure of compound **4** the central aliphatic chain of one of the ligands was found disordered over two positions that were refined with site occupancy factors of 0.68 and 0.32, respectively. Four acetone molecules were found in the structure; three of them were found disordered over two positions each: the first one was refined with site occupancy factors of 0.72 and 0.28, respectively; the second one was refined with site occupancy factors of 0.35 and 0.15, respectively; the third one was refined with site occupancy factors of 0.22 and 0.18, respectively; and the fourth one was refined with overall site occupancy of 0.5. Compound **5** was refined as a 2-component twin and refined using reflections form the hkl5 file. In the structure of compound **6** the coordinated THF molecule was found to be disordered over two positions that were refined with site occupancy factors of 0.5 each. The uncoordinated THF molecule was found disordered over two positions that were refined with site occupancy factors of 0.75 and 0.25, respectively. In the structure of compound **7** two crystallographically distinct metal sites were present, each containing two different disordered metal atoms (Nd and Dy). At both sites, the metal atoms were refined with constrained occupancies summing to 1. The metal atoms were refined anisotropically with the same atomic coordinates by using the EXYZ constraint to maintain identical positions, and EADP was applied to constrain their atomic displacement parameters. Additionally, the ethanol molecules coordinated to the second metal site were found in one position (in accordance with the occupancy of the Dy atom in that site, 0.31 s.o.f.) and disordered over two positions (in accordance with the occupancy of the Nd atom in that site, 0.69 s.o.f.). The occupancy and position of the two disordered metals in the structure were assigned according to the better refinement parameters and residual electron density map compared to the refinements without disorder and with Dy in site 1 and Nd in site 2 (Figure S41 and Table S13). In the structure of compound **8**, one of the coordinated ethanol molecules was found disordered over two positions that were refined with site occupancy factors of 0.56 and 0.44, respectively. The complex has an overall negative charge, meaning that the charge balance must be provided by one cation per complex. As a solvent mask was used due to the presence of a large solvent-accessible void, the electronic density was consistent with the presence of 1­[Et_3_NH^+^] (coming from the synthesis) and 8­[EtOH] per formula unit. This accounted for a total of 910 electrons per unit cell.

### 4.3 ESI-MS

Electrospray ionization mass spectrometry (ESI-MS) analyses of the complexes were performed by using an Agilent 1260 Infinity II HPLC system equipped with an Agilent InfinityLab LC/MSD XT detector and an Agilent Jet Stream ESI source. The samples were prepared by dissolving 1 mg of each compound in 1 mL of solvent (MeOH or ACN) to obtain stock solutions. Working solutions were then prepared at a final concentration of approximately 150 μg/mL. Samples with limited solubility were filtered through a 0.22 μm PTFE microfilter prior to analysis.

### 4.4 ICP-OES Analysis

The metal content of the sample was determined via Inductively Coupled Plasma Atomic Emission Spectroscopy (ICP-OES). From 5 to 15 mg of solid sample were suspended in 5 mL of HNO_3_ 65% and 1 mL of H_2_O_2_ 30%, then digested in a Milestone microwave MLS-1200 MEGA (digestion sequence: 1 min at 250 W, 1 min at 0 W, 5 min at 250 W, 5 min at 400 W, 5 min at 650 W, 5 min of cooling). The solutions were diluted to 50 mL with bidistilled water and analyzed using an emission spectrometer JY 2501 with coupled plasma induction in radial configuration HORIBA Jobin Yvon (Kyoto, Japan), ULTIMA2 model. Instrumental features: monochromator Model JY 2501; focal length 1 m; resolution 5 pm; nitrogen flow 2 L/min. ICP source: nebulizer Meinhard, cyclonic spraying chamber; argon flow 12 L/min; wavelength range 160–785 nm; optical bench temperature 32 °C. The wavelength used for quantitative analysis was chosen by examining the emission line with greater relative intensity, ensuring that there was no spectral interference with the Argon emission lines. Acquisition parameters: Nd (nm): 410.946 and Dy (nm) 394.469; Voltage (V): 580; gain: 100. The quantitative analysis was performed after the acquisition of a calibration line using standard solutions in HNO_3_ 10% to simulate the final acidity of the samples; the concentration range of the standards varied from 0.1 to 50 mg/L of Nd and Dy. Data acquisition and processing were performed using ICP JY v 5.2 software (Jobin Yvon). Measurements were performed in triplicate, and the syntheses were performed in duplicates.

### 4.5 ESEM-EDS

Environmental scanning electron microscope (ESEM) Quanta 250 FEG (FEI, Hillsboro, OR) equipped with an energy-dispersive spectrometer (EDS) for X-ray microanalysis (Bruker Nano GmbH, Berlin, Germany) was employed. The EDS had a QUANTAX XFlash 6–30 Detector with an energy resolution of ≤126 eV fwhm at Mn Kα. ESPRIT 1.9 microanalysis software (Bruker Nano GmbH) was used for X-ray spectra acquisition and for the digital element map. EDS analysis was carried out with an accelerating voltage of 30 kV; a spot size of 4.0; a final lens aperture of 40 μm, a WD of 10 mm, and a live time of 50 s.

### 4.6 General Procedure for Synthesis of H_3_L^R^ Ligands

The syntheses described below are based on procedures previously reported in the literature^84^ and no uncommon hazards were noted. Salicylaldehydes (2 mmol) were dissolved in absolute ethanol (60 mL) at 50 °C under magnetic stirring.1,3-Diaminopropan-2-ol (1 mmol) was then added to the mixture. The resulting yellow solution was stirred at reflux for 5 h. The crude product was concentrated under reduced pressure, resulting in a yellow oil, which was subsequently crystallized overnight at −4 °C in an ethanol/hexane mixture. The product was then isolated by filtration, washed with cold ethanol and hexane, and dried under vacuum, yielding a yellow crystalline solid. ^1^H spectra and ESI-MS of the synthesized molecules are reported in Figures S1–S6.

#### 4.6.1 H_3_L^H^

Yellow solid, 85% yield. ^1^H NMR (400 MHz, DMSO-*d*_6_): δ 13.55 (s, 2H, OH), 8.55 (s, 2H, CH–N_imine_), 7.46 (dd, *J* = 7.6, 1.7 Hz, 2H, Ar), 7.37–7.30 (dt, 2H, Ar), 6.94–6.86 (m, 4H, Ar), 5.22 (d, *J* = 5.3 Hz, 1H, OH), 4.03 (m, 1H, CH), 3.79 (ddd, *J* = 12.2, 4.4, 1.3 Hz, 2H, CH_2_–N), 3.62 (ddd, *J* = 12.1, 6.6, 1.1 Hz, 2H, CH_2_–N). ESI-MS (MeOH): *m*/*z* 297 [H_2_L^H^]^−^. Elemental Analysis [Calc. for C_17_H_18_N_2_O_3_(H_2_O)_0.2_, %]: C, 67.60; H, 6.14; N, 9.28. [Found %]: C, 67.64; H, 6.03; N, 9.22. FTIR (ATR, ν_max_/cm^–1^): 3375 (br), 2896 (w), 1631 (m), 1576 (m), 1495 (m), 1418 (m), 1274 (s), 1203 (w), 1147 (w), 1084 (w), 1048 (w), 1025 (w), 972 (w), 940 (w), 885 (m), 841 (m), 750 (s), 657 (m), 640 (w), 555 (w), 446 (m).

#### 4.6.2 H_3_L^p‑OMe^

Yellow solid, 87% yield. ^1^H NMR (400 MHz, DMSO-*d*_6_): δ 12.88 (s, 2H, OH), 8.51 (s, 2H, CH–N_imine_), 7.06 (d, *J* = 3.1 Hz, 2H, Ar), 6.96 (dd, *J* = 8.9, 3.1 Hz, 2H, Ar), 6.83 (d, *J* = 8.9 Hz, 2H, Ar), 5.19 (d, *J* = 5.3 Hz, 1H, OH), 4.02 (m, 1H, CH), 3.78 (ddd, *J* = 12.0, 4.4, 1.3 Hz, 2H, CH_2_–N), 3.72 (s, 6H, O–CH3), 3.60 (ddd, *J* = 12.1, 6.6, 1.1 Hz, 2H, CH_2_–N). ESI-MS (MeOH): *m*/*z* = 357 [H_2_L^p‑OMe^]^−^. Elemental Analysis [Calcd for C_19_H_22_N_2_O_5_, %]: C, 63.65; H, 6.19; N, 7.81. [Found %]: C, 63.81; H, 6.26; N, 7.85. FTIR (ATR, ν_max_/cm^–1^): 3352 (br), 2900 (w), 2833 (w), 1635 (m), 1588 (m), 1490 (s), 1456 (m), 1395 (w), 1326 (m), 1269 (m), 1221 (m), 1159 (s), 1084 (w), 1029 (s), 851 (m), 819 (s), 784 (s), 668 (w), 602 (w), 461 (m).

#### 4.6.3 H_3_L^o‑tBu^

Yellow solid, 84% yield. ^1^H NMR (400 MHz, CD_3_CN-*d*_3_): δ 14.23 (s, 2H, OH), 8.49 (s, 2H, CH–N_imine_), 7.38 (dd, *J* = 7.8, 1.7 Hz, 2H, Ar), 7.25 (dd, *J* = 7.6, 1.7 Hz, 2H, Ar), 6.87 (t, *J* = 7.7 Hz, 2H, Ar), 4.19 (m, 1H, CH), 3.86 (ddd, *J* = 12.4, 4.3, 1.3 Hz, 2H, CH_2_–N), 3.68 (ddd, *J* = 12.5, 6.9, 1.2 Hz, 2H, CH_2_–N), 3.43 (d, *J* = 5.4 Hz, 1H, OH), 1.45 (s, 18H, C­(CH_3_)_3_). ESI-MS (MeOH): *m*/*z* 409 [H_2_L^o‑tBu^]^−^. Elemental Analysis [Calc. for C_25_H_34_N_2_O_3_, %]: C, 73.13; H, 8.34; N, 6.82. [Found %]: C, 72.81; H, 8.37; N, 7.12. FTIR (ATR, ν_max_/cm^–1^): 2955 (w), 2902 (w), 1631 (m), 1483 (w), 1434 (s), 1390 (w), 1359 (w), 1306 (m), 1265 (m), 1199 (m), 1144 (m), 1088 (m), 1037 (m), 853 (m), 794 (m), 748 (s), 679 (w), 626 (w), 548 (w).

#### 4.6.4 Procedure A—Synthesis of REE Complexes (1:1 REE:H_3_L^R^ Ratio)

A yellow solution of H_3_L^R^ (1.05 equiv) and Ln­(NO_3_)_3_·XH_2_O (1 equiv) in 96% ethanol (4 mL) was stirred at 55 °C for a few minutes before adding triethylamine (3.15 equiv). Upon addition of the base, a precipitate formed, and the resulting mixture was stirred for 2 h. After the reaction, the solid was filtered, washed with a mixture of ethanol/hexane, and dried under a vacuum, yielding pale-yellow solids. Elemental analyses were performed on the solid obtained, often containing a mixture of oligomeric species.

#### 4.6.5 Compound A1 (Dy:H_3_L^H^)

Pale-yellow solid, yield 10%. Elemental Analysis [Calc. for ((Dy_3_(C_17_​H_15_​N_2_O_3_)_2_​(C_17_H_16_​N_2_O_3_)​​(OH)­(H_2_O)_2_)_0.6_​(Dy_6_(C_17_H_15_N_2_O_3_)_4_​(OH)_2_​(NO_3_)_4_​(H_2_O)_2_)_0.4_), (MW: 1846.22 g/mol) %] C, 37.6; H, 3.11; N, 6.37; [Found %] C, 37.67; H, 3.40; N, 6.65. FTIR (ATR, ν_max_/cm^–1^): 3420 (br, O–H stretching), 2988 (br), 2900 (br), 1632 (s, C=N stretching), 1598 (m), 1543 (w), 1473 (m), 1448 (m), 1399 (m), 1291 (br), 1198 (w), 1152 (w), 1127 (w), 1033 (w), 890 (w), 869 (w), 794 (w), 761 (m), 742 (w), 710 (w), 646 (w), 635 (w), 595 (w), 566 (w), 515 (w), 478 (w). Crystals of **3** were obtained by recrystallization through the slow evaporation of acetone after 3 weeks. Crystals of **2** were obtained by recrystallization through the slow evaporation of DMF at high temperature over a period of one month.

#### 4.6.6 Compound A2 (Nd:H_3_L^H^)

Pale-yellow solid, yield 16%. Elemental Analysis [Calc. for ((Nd_3_(C_17_H_15_​N_2_O_3_)_2_​(C_17_H_16_​N_2_O_3_)­(OH))_0.9_​(Nd_6_(C_17_H_15_​N_2_O_3_)_4_(OH)_4_​(NO_3_)_2_​(H_2_O)_2_)_0.1_), (MW: 1430.48 g/mol) %] C, 44.25; H, 3.46; N, 6.27; [Found %] C, 44.42; H, 3.50; N, 6.34. FTIR (ATR, ν_max_/cm^–1^): 2899 (br), 2827 (br), 1624 (s, C=N stretching), 1596 (w), 1544 (m), 1474 (m), 1443 (m), 1405 (m), 1349 (w), 1317 (m), 1295 (s), 1228 (w), 1195 (m), 1156 (w), 1113 (m), 1040 (m), 1016 (m), 964 (w), 904 (w), 882 (w), 854 (m), 789 (m), 759 (s), 732 (m), 642 (w), 597 (m), 590 (m), 510 (m), 479 (w).

#### 4.6.7 Compound A3 (Dy:H_3_L^p‑OMe^)

Orange solid, yield 17%. Elemental Analysis [Calc. for ((Dy_3_​(C_19_H_19_​N_2_O_5_)​(C_19_H_20_​N_2_O_5_)_2_​(OH)_2_)_0.7_​(Dy_6_(C_19_H_19_​N_2_O_5_)_4_(OH)_4_​(NO_3_)_2_​(H_2_O)_2_)_0,3_), (MW: 1900.10 g/mol) %] C, 39.63; H, 3.6; N, 5.31; [Found %] C, 39.71; H, 3.70; N, 5.44. FTIR (ATR, ν_max_/cm^–1^): 3367 (br, O–H stretching), 2886 (br), 2829 (br), 1629 (m), 1606 (w), 1539 (w), 1473 (s), 1439 (w), 1423 (w), 1386 (w), 1312 (w), 1261 (m), 1221 (m), 1153 (m), 1111 (w), 1034 (s), 952 (w), 909 (w), 810 (s), 770, 705 (w), 660 (w), 571 (w), 505 (w), 478 (w), 428 (w). Crystals of **1** were obtained by crystallization through slow evaporation of the reaction mixture after one month.

#### 4.6.8 Compound A4 (Nd:H_3_L^p‑OMe^)

Orange solid, yield 14%. Elemental Analysis [Calc. for (Nd_4_​(C_19_H_20_​N_2_O_5_)_3_​(C_19_H_19_​N_2_O_5_)​​(C_19_H_21_​N_2_O_5_)​(NO_3_)​(OH)​(H_2_O)​(C_2_​H_5_OH)), (MW: 2501.92) %] C, 46.57; H, 4.39; N, 6.16; [Found %] C, 46.71; H, 4.10; N, 5.97. FTIR (ATR, ν_max_/cm^–1^): 3356 (br, O–H stretching), 3021 (w), 2992 (w), 2900 (w), 2831 (br), 1633 (m, C=N stretching), 1608 (w), 1547 (w), 1480 (s), 1390 (m), 1348 (w), 1315 (m), 1288 (s), 1272 (m), 1224 (m), 1159 (m), 1112 (m), 1039 (m), 1015 (w), 955 (w), 851 (w), 808 (s), 773 (w), 747 (w), 660 (w), 584 (w), 547 (w), 502 (w), 482 (w), 426 (w).

#### 4.6.9 Compound A5 (Dy:H_3_L^o‑tBu^)

Pale-yellow solid, yield 33%. Elemental Analysis [Calc. for (Dy_2_​(C_25_H_31_​N_2_O_3_)_2_​(H_2_O)_2_), (MW: 1176.08 g/mol) %] C, 51.06; H, 5.66; N, 4.76; [Found %] C, 51.31; H, 5.83; N, 4.58. FTIR (ATR, ν_max_/cm^–1^): 3585 (br, O–H stretching), 2949 (w), 2904 (w), 2845 (w), 1627 (s, C=N stretching), 1590 (m), 1545 (m), 1448 (w), 1422 (s), 1383 (m), 1358 (w), 1314 (m), 1232 (w), 1186 (m), 1141 (m), 1118 (w), 1093 (w), 1051 (m), 1035 (m), 984 (w), 910 (w), 862 (m), 807 (w), 756 (s), 711 (m), 683 (w), 637 (w), 622 (w), 568 (w), 543 (w), 529 (w), 503 (m), 456 (w). Crystals of **5** and **6** were obtained respectively by slow evaporation of the crude reaction mixture in ethanol and by recrystallization of the purified solid through slow evaporation from THF after 2 weeks.

#### 4.6.10 Compound A6 (Nd:H_3_L^o‑tBu^)

Pale-yellow solid, yield 22%. Elemental Analysis [Calc. for (Nd_4_(C_25_​H_31_N_2_​O_3_)_2_​(NO_3_)_3_(OH)_3_​(H_2_O)_2.8_​(C_2_H_5_​OH)_1.3_), (MW: 1739.38 g/mol) %] C, 36.32; H, 4.54; N, 5.64; [Found %] C, 36.39; H, 4.70; N, 5.50. FTIR (ATR, ν_max_/cm^–1^): 3523 (br, O–H stretching), 2950 (w), 2902 (br), 1626 (s, C=N stretching), 1590 (w), 1548 (w), 1481 (br), 1424 (s), 1398 (s), 1311 (s), 1294 (m), 1232 (w), 1188 (w), 1143 (m), 1117 (w), 1093 (w), 1042 (m), 868 (m), 807 (w), 749 (s), 680 (br), 640 (s), 553 (m), 530 (m), 470 (m). Crystals of **8** were obtained by crystallization through slow evaporation of the reaction mixture after 1 week.

#### 4.6.11 Procedure B—Synthesis of REE Complexes (6:4 REE:H_3_L^R^ Ratio)

A yellow solution of H_3_L^R^ (4 equiv) and Ln­(NO_3_)_3_·XH_2_O (6 equiv) in 96% ethanol (4 mL) was stirred at 55 °C for a few minutes before adding triethylamine (12 equiv). Upon the addition of the base, a precipitate formed, and the resulting mixture was stirred for 2 h. After the reaction, the solid was filtered, washed with a mixture of ethanol/hexane, and dried under vacuum, yielding pale-yellow solids. Elemental analysis was performed on the solid obtained, often containing a mixture of oligomeric species.

#### 4.6.12 Compound B1 (Dy:H_3_L^H^)

Pale-yellow solid, yield 18%. Elemental Analysis [Calc. for ((Dy_6_​(C_17_H_15_​N_2_O_3_)_4_​(OH)_4_(NO_3_)_2_​(H_2_O)_4_)_0.6_​(Dy_9_(C_17_H_15_​N_2_O_3_)_3_​(C_17_H_16_​N_2_O_3_)_2_​(NO_3_)_10_​(OH)_4_(H_2_O)_4_)_0,4_) (MW: 2932.70 g/mol) %] C, 30.63; H, 2.71; N, 6.69; [Found %] C, 30.74; H, 3.14; N, 6.99. FTIR (ATR, ν_max_/cm^–1^): 3594 (br), 2889 (br), 2842 (w), 1629 (s), 1598 (w), 1541 (w), 1469 (w), 1447 (s), 1401 (m), 1346 (s), 1327 (m), 1298 (m), 1243 (w), 1194 (w), 1146 (w), 1127 (w), 1058 (w), 1028 (w), 894 (w), 864 (w), 794 (w), 754 (m), 739 (m), 704 (w), 649 (w), 630 (w), 597 (w), 564 (w), 519 (w), 478 (m).

#### 4.6.13 Compound B2 (Nd:H_3_L^H^)

Pale-yellow solid, yield 19%. Elemental Analysis [Calc. for ((Nd_6_(C_17_​H_15_N_2_O_3_)_4_​(OH)_4_(NO_3_)_2_)​_0,15_(Nd_3_(C_17_​H_15_N_2_O_3_)_2_​(C_17_H_16_​N_2_O_3_)​​(NO_3_))_0,85_) (MW: 1467.38 g/mol) %] C, 42.59; H, 3.25; N, 6.91; [Found %] C, 42.59; H, 3.51; N, 7.3. FTIR (ATR, ν_max_/cm^–1^): 2899 (w), 2826 (w), 1627 (s), 1596 (w), 1544 (m), 1475 (s), 1444 (m), 1405 (m), 1350 (w), 1336 (m), 1317 (m), 1297 (s), 1228 (w), 1195 (m), 1157 (w), 1113 (m), 1040 (w), 1016 (w), 964 (w), 904 (w), 882 (w), 854 (m), 788 (m), 761 (s), 732 (w), 642 (w), 598 (m), 510 (s), 480 (m).

#### 4.6.14 Compound B3 (Dy:H_3_L^p‑OMe^)

Pale-yellow solid, yield 11%. Elemental Analysis [Calc. for (Dy_6_(C_19_​H_19_N_2_O_5_)_4_​(OH)_4_(NO_3_)_3_​(H_2_O)​(C_6_​H_16_N))_0,7_​(Dy_9_(C_19_​H_19_N_2_O_5_)_3_​(C_19_H_20_​N_2_O_5_)_2_​(NO_3_)_10_​(OH)_4_)_0,3_) (MW: 3118.33 g/mol) %] C, 33.09; H, 3.2; N, 6.47; [Found %] C, 33.09; H, 3.35; N, 6.66. FTIR (ATR, ν_max_/cm^–1^): 3461 (br), 2897 (w), 2831 (w), 1637 (s), 1609 (w), 1545 (m), 1509 (w), 1478 (s), 1424 (w), 1388 (m), 1365 (w), 1284 (s), 1269 (s), 1224 (m), 1157 (s), 1119 (w), 1033 (s), 953 (w), 901 (w), 812 (s), 773 (m), 739 (w), 707 (w), 638 (m), 569 (w), 513 (m), 482 (m), 429 (w).

#### 4.6.15 Compound B4 (Nd:H_3_L^p‑OMe^)

Pale-yellow solid, yield 10%. Elemental Analysis [Calc. for (Nd_4_(C_19_​H_19_N_2_O_5_)​​(C_19_H_20_​N_2_O_5_)_2_​(NO_3_)_5_​(H_2_O)_3_​(C_2_H_5_OH))% (MW: 2055.21 g/mol)] C, 34.48; H, 3.48; N, 7.50; [Found %] C, 34.31; H, 3.74; N, 7.69. FTIR (ATR, ν_max_/cm^–1^): 3393 (br), 2898 (w), 2832 (w), 1636 (m), 1610 (w), 1538 (w), 1478 (s), 1442 (m), 1390 (m), 1272 (s), 1223, 1159 (s), 1113 (w), 1033 (s), 954 (w), 810 (s), 772 (w), 735 (w), 629 (w), 563 (w), 503 (w), 478 (w), 425 (w).

## Supplementary Material



## Notes

NMR, ESI-MS, UV–vis experiments, single crystal X-ray structural characterization (PDF)

## Abbreviations

REEs: rare earth elements
PM: permanent magnets
NdFeB: neodymium iron boron
EoL: end-of-life
LREEs: light rare earth elements
HREEs: heavy rare earth elements
Ln: lanthanide
Nd: neodymium
Dy: dysprosium
c.n.: coordination number
DMF: *N*,*N*-dimethylformamide
CAN: acetonitrile
THF: tetrahydrofuran
TEA: triethylamine
