# Supplementary material for: Polynuclear Complexes of Nd and Dy with N2O3 Donor Ligands: Solution Speciation and Selective Precipitation Studies
Source: Inorg Chem. 2025 Oct 24;64(44):21932–47. doi: 10.1021/acs.inorgchem.5c03477 (PMC12606718; doi:10.1021/acs.inorgchem.5c03477)
Supplement: Supplementary file 1 [file ic5c03477_si_001.pdf]

SUPPORTING INFORMATION

Polynuclear complexes of Nd and Dy with N_2O_3 donor ligands. Solution speciation and selective precipitation studies.

Alex Falco,[a] Alessia Panizzi,[a] Matteo Melegari,[a] Fabio Fornari,[a] Monica Maffini,[a] Matteo Tegoni,[a] Angela Serpe,[b,c]* Nicola Demitri,[d] Luciano Marchiò [a]*

[a] *Department of Chemistry, Life Sciences and Environmental Sustainability, University of Parma, Parco Area delle Scienze 17/A, 43124, Parma, Italy.*

[b] *Department of Civil and Environmental Engineering and Architecture (DICAAR), and research unit of INSTM, University of Cagliari, Via Marengo 2, 09123 Cagliari, Italy.*

[c] *Environmental Geology and Geoengineering Institute of the National Research Council (IGAG-CNR), Piazza d'Armi, 09123 Cagliari, Italy.*

[d] *Elettra – Light Source, S.S. 14 Km 163.5 in Area Science Park, 34149 Basovizza, Trieste, Italy.*

Corresponding author: luciano.marchio@unipr.it, serpe@unica.it

Table of contents

NMR spectra of the H_3L^R ligands..........S2
ESI-MS of H_3L^R ligands..........S4
Crystallographic Data..........S6
UV-Visible measurements and speciation..........S36
Separation experiments..........S37
ESI-MS of Dy, Nd, and Y complexes with H_3L^R..........S38

NMR spectra of the H_3L^R ligands

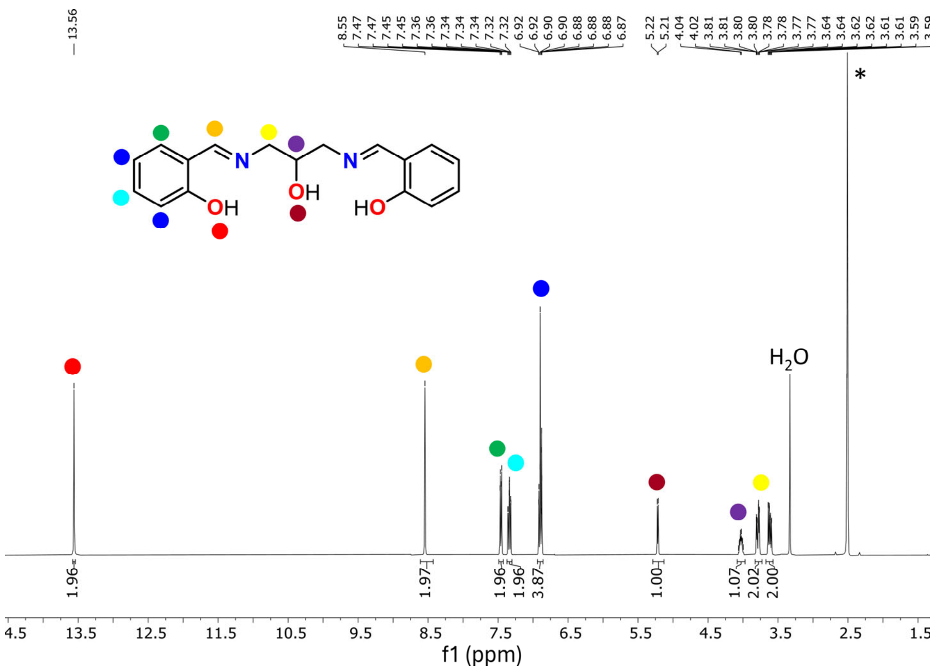

Figure S1. ^{1}H NMR spectrum of H_3L^H in DMSO-d$_6$, 400 MHz, 298 K. * residual solvent signal.

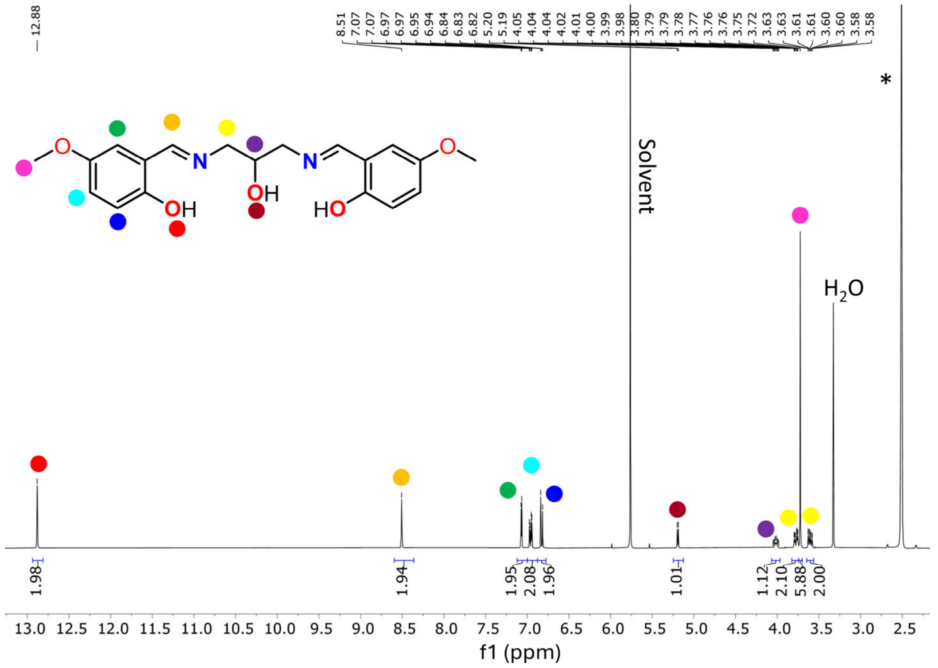

Figure S2. ^{1}H NMR of $H_3L^{p\text{-}OMe}$ in DMSO-d$_6$, 400 MHz, 298 K. * residual solvent signal.

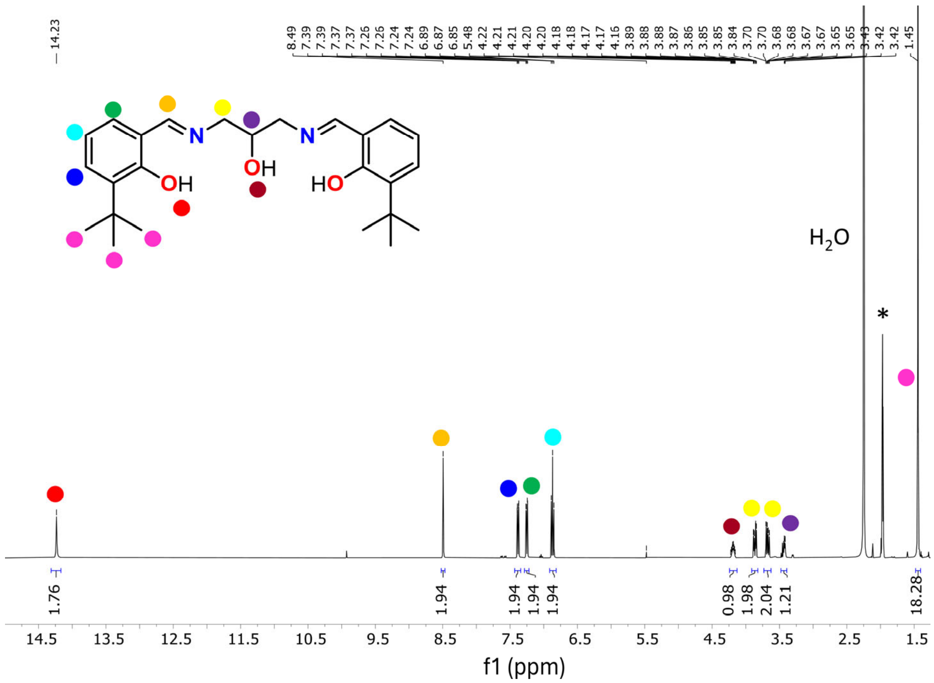

Figure S3. ^{1}H NMR of $H_3L^{o\text{-}tBu}$ in CD_3CN-d_3, 400 MHz, 298 K. * residual solvent signal.

ESI-MS of H_3L^R ligands

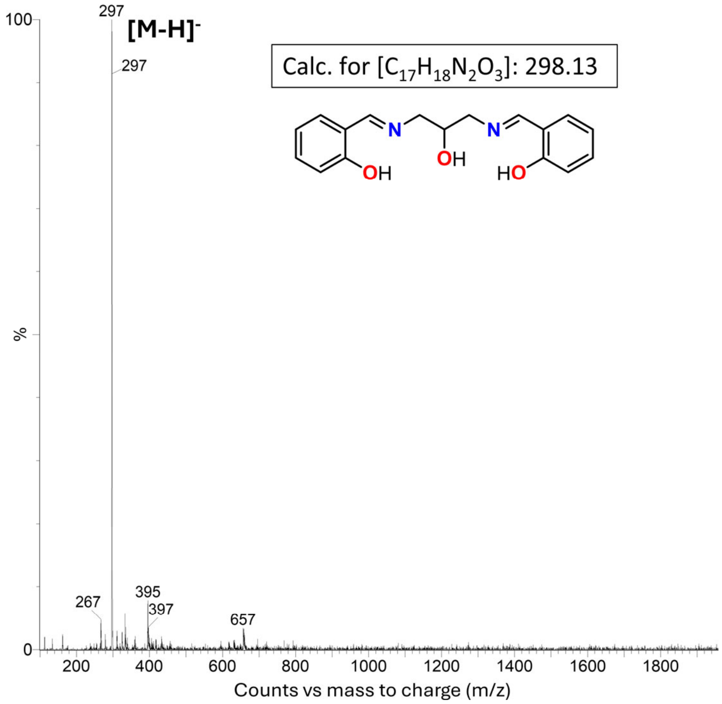

Figure S4. ESI-MS of H_3L^H in negative mode, C = 100 µg/mL in ACN.

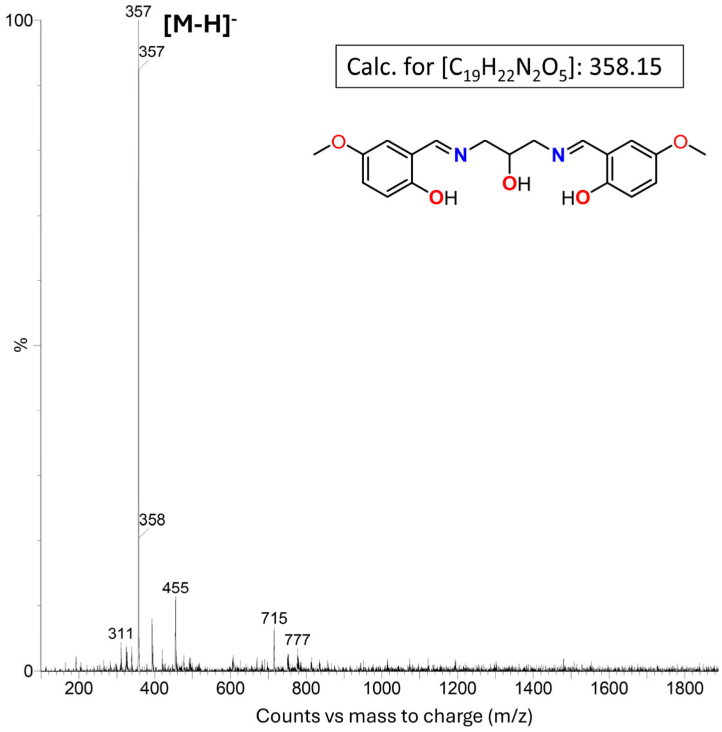

Figure S5. ESI-MS of $H_3L^{p\text{-}OMe}$ in negative mode, C = 100 µg/mL in ACN.

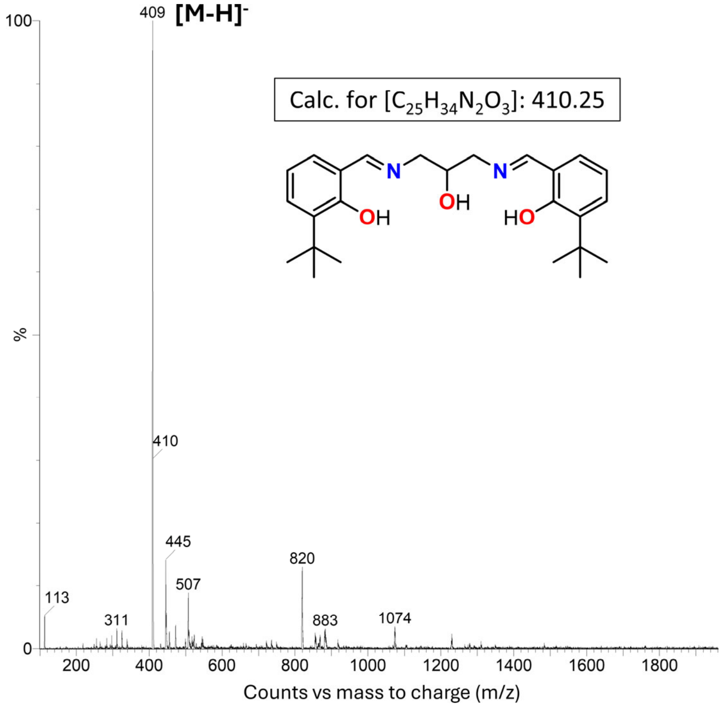

Figure S6. ESI-MS of $H_3L^{o\text{-}tBu}$ in negative mode, C = 100 μg/mL in ACN.

Crystallographic Data

Table S1. Summary of X-ray crystallographic data for compounds **1** and **2**.

Identification code	$[Dy_3(HL^{p-OMe})_2(L^{p-OMe})(OH)(DMF)_2]NO_3 \cdot (DMF)_2$ (**1**)	$[Dy_4(HL^H)(L^H)_2(OH)_2(HCOO)(H_2O)_2(DMF)]$ $[Dy_4(HL^H)(L^H)_2(OH)_2(HCOO)(H_2O)(DMF)_2](NO_3)_2 \cdot (H_2O)_{0.2} \cdot (DMF)_{2.75}$ (**2**)
Empirical formula	$C_{69}H_{88}Dy_3N_{11}O_{23}$	$C_{64.62}H_{84.33}Dy_4N_{9.88}O_{22.48}$
Formula weight	1927.00	2009.09
Temperature/K	100.00	200.00
Crystal system	monoclinic	triclinic
Space group	P21/c	P-1
a/Å	20.565(4)	17.0290(5)
b/Å	17.805(4)	19.0630(6)
c/Å	23.359(5)	27.0387(7)
α/°	90	107.216(2)
β/°	105.29(3)	97.737(2)
γ/°	90	108.659(2)
Volume/Å^3	8250(3)	7684.2(4)
Z	4	4
ρcalcg/cm^3	1.551	1.737
μ/mm^{-1}	1.938	21.093
F(000)	3844.0	3940.0
Crystal size/mm^3	0.1 × 0.05 × 0.02	0.13 × 0.09 × 0.07
Radiation	Synchrotron (λ = 0.620)	CuKα (λ = 1.54178)
2Θ range for data collection/°	1.79 to 43.312	3.536 to 145.196
Index ranges	-24 ≤ h ≤ 24, -21 ≤ k ≤ 21, -27 ≤ l ≤ 27	-21 ≤ h ≤ 20, -23 ≤ k ≤ 22, 0 ≤ l ≤ 33
Reflections collected	33103	30283
Independent reflections	14251 [R_{int} = 0.1671, R_{sigma} = 0.1932]	30283 [R_{int} = 0.1071, R_{sigma} = 0.0554]
Data/restraints/parameters	14251/481/938	30283/195/1692
Goodness-of-fit on F^2	1.025	0.999
Final R indexes [I>=2σ (I)]	R_1 = 0.0883, wR_2 = 0.2168	R_1 = 0.0655, wR_2 = 0.1839
Final R indexes [all data]	R_1 = 0.1937, wR_2 = 0.2688	R_1 = 0.0819, wR_2 = 0.1970
Largest diff. peak/hole / e Å^{-3}	1.90/-1.44	1.24/-1.52

Table S2. Summary of X-ray crystallographic data for compounds **3** and **4**.

Identification code	$[Dy_6(L^H)_4(OH)_4(NO_3)_2(H_2O)_2]\cdot(CH_3COCH_3)_{1.66}$ (**3**)	$[Dy_6(L^{p\text{-}OMe})_4(OH)_4(NO_3)_2(H_2O)_2]\cdot(H_2O)_{0.5}\cdot(CH_3COCH_3)_{2.33}$ (**4**)
Empirical formula	$C_{77.99}H_{87.98}Dy_6N_{10}O_{27.33}$	$C_{89.98}H_{113.96}Dy_6N_{10}O_{37.66}$
Formula weight	2577.72	2913.18
Temperature/K	200.00	200.00
Crystal system	orthorhombic	monoclinic
Space group	Pbca	$P2_1/n$
a/Å	21.466(4)	15.0026(7)
b/Å	19.837(4)	17.9709(7)
c/Å	21.147(4)	22.2907(10)
α/°	90	90
β/°	90	102.250(2)
γ/°	90	90
Volume/Å^3	9005(3)	5873.0(4)
Z	4	2
ρcalcg/cm^3	1.901	1.647
μ/mm^{-1}	4.994	3.846
F(000)	4962.0	2842.0
Crystal size/mm^3	0.1 × 0.07 × 0.04	0.05 × 0.03 × 0.02
Radiation	MoKα (λ = 0.71073)	MoKα (λ = 0.71073)
2Θ range for data collection/°	4.106 to 51.36	3.664 to 56.562
Index ranges	-26 ≤ h ≤ 25, -24 ≤ k ≤ 23, -25 ≤ l ≤ 25	-20 ≤ h ≤ 20, -23 ≤ k ≤ 23, -29 ≤ l ≤ 29
Reflections collected	91585	383845
Independent reflections	8509 [R_{int} = 0.0758, R_{sigma} = 0.0308]	14555 [R_{int} = 0.0981, R_{sigma} = 0.0323]
Data/restraints/parameters	8509/85/594	14555/444/870
Goodness-of-fit on F^2	1.060	1.119
Final R indexes [I>=2σ (I)]	R_1 = 0.0304, wR_2 = 0.0736	R_1 = 0.0357, wR_2 = 0.0901
Final R indexes [all data]	R_1 = 0.0410, wR_2 = 0.0803	R_1 = 0.0583, wR_2 = 0.1064
Largest diff. peak/hole / e Å^{-3}	1.69/-0.55	1.81/-0.68

Table S3. Summary of X-ray crystallographic data for compounds **5** and **6**.

Identification code	$[Dy_2(L^{o\text{-}tBu})_2(EtOH)_2]\cdot EtOH$ (**5**)	$[Dy_2(L^{o\text{-}tBu})_2(THF)_2]\cdot THF$ (**6**)
Empirical formula	$C_{56}H_{80}Dy_2N_4O_9$	$C_{66}H_{94}Dy_2N_4O_{10}$
Formula weight	1278.24	1428.45
Temperature/K	200.00	200.0
Crystal system	triclinic	monoclinic
Space group	P-1	$P2_1/c$
a/Å	11.5031(12)	12.074(5)
b/Å	16.6716(18)	15.733(4)
c/Å	16.7712(18)	17.452(5)
α/°	77.502(4)	90
β/°	74.697(4)	95.610(18)
γ/°	69.901(4)	90
Volume/Å^3	2885.1(5)	3299.4(18)
Z	2	2
ρcalcg/cm^3	1.471	1.438
μ/mm^{-1}	2.625	2.305
F(000)	1296.0	1460.0
Crystal size/mm^3	0.08 × 0.05 × 0.04	0.15 × 0.10 × 0.05
Radiation	MoKα (λ = 0.71073)	MoKα (λ = 0.71073)
2Θ range for data collection/°	3.854 to 51.36	4.266 to 52.856
Index ranges	-14 ≤ h ≤ 14, -20 ≤ k ≤ 20, -20 ≤ l ≤ 20	-15 ≤ h ≤ 15, -19 ≤ k ≤ 19, -21 ≤ l ≤ 21
Reflections collected	21567	65200
Independent reflections	21567 [R_{int} = 0.0510, R_{sigma} = 0.0571]	6753 [R_{int} = 0.0653, R_{sigma} = 0.0300]
Data/restraints/parameters	21567/0/659	6753/232/448
Goodness-of-fit on F^2	1.050	1.039
Final R indexes [I>=2σ (I)]	R_1 = 0.0510, wR_2 = 0.1283	R_1 = 0.0235, wR_2 = 0.0486
Final R indexes [all data]	R_1 = 0.0620, wR_2 = 0.1376	R_1 = 0.0325, wR_2 = 0.0526
Largest diff. peak/hole / e Å^{-3}	1.04/-0.76	0.37/-0.49

Table S4. Summary of X-ray crystallographic data for compounds **7** and **8**.

Identification code	$[NdDy(L^{o\text{-}tBu})_2(EtOH)_{2.69}]\cdot EtOH\cdot(H_2O)_{0.2}$ (**7**)	$Et_3NH[Nd_7(L^H)_4(OH)_6(NO_3)_4(EtOH)_4]\cdot(EtOH)_8$ (**8**)
Empirical formula	$C_{57.39}H_{84.56}DyN_4NdO_{9.89}$	$C_{130}H_{218}N_{13}Nd_7O_{42}$
Formula weight	1295.56	3644.84
Temperature/K	200.00	200.00
Crystal system	monoclinic	monoclinic
Space group	$P2_1/c$	C2/c
a/Å	17.5719(8)	30.769(3)
b/Å	16.0394(7)	15.7058(15)
c/Å	22.9646(10)	32.815(3)
α/°	90	90
β/°	111.9000(10)	105.736(3)
γ/°	90	90
Volume/Å^3	6005.3(5)	15263(2)
Z	4	4
ρcalcg/cm^3	1.433	1.586
μ/mm^{-1}	2.146	2.415
F(000)	2648.0	7380.0
Crystal size/mm^3	0.05 × 0.03 × 0.02	0.06 × 0.05 × 0.05
Radiation	MoKα (λ = 0.71073)	MoKα (λ = 0.71073)
2Θ range for data collection/°	3.574 to 51.362	4.146 to 51.362
Index ranges	-21 ≤ h ≤ 21, -18 ≤ k ≤ 19, -28 ≤ l ≤ 28	-37 ≤ h ≤ 37, -19 ≤ k ≤ 16, -40 ≤ l ≤ 37
Reflections collected	91162	190984
Independent reflections	11383 [R_{int} = 0.0977, R_{sigma} = 0.0771]	14384 [R_{int} = 0.1178, R_{sigma} = 0.0579]
Data/restraints/parameters	11383/248/766	14384/50/770
Goodness-of-fit on F^2	1.021	1.004
Final R indexes [I>=2σ (I)]	R_1 = 0.0530, wR_2 = 0.1003	R_1 = 0.0511, wR_2 = 0.1161
Final R indexes [all data]	R_1 = 0.1053, wR_2 = 0.1198	R_1 = 0.0738, wR_2 = 0.1301
Largest diff. peak/hole / e Å^{-3}	1.77/-0.48	2.68/-0.67

Table S5. Selected geometric parameters (Å) for compounds **1-8**.

[$Dy_3(HL^{p\text{-}OMe})_2(L^{p\text{-}OMe})(OH)(DMF)_2]NO_3\cdot(DMF)_2$ (**1**)					
Dy1-Dy2	3.5683(14)	Dy1-N13	2.523(18)	Dy2-N12	2.540(18)
Dy1-Dy3	3.5530(13)	Dy2-Dy3	3.5542(18)	Dy3-O1H	2.393(9)
Dy1-O14	2.336(12)	Dy2-O14	2.391(10)	Dy3-O1	2.376(11)
Dy1-O1H	2.449(9)	Dy2-O1H	2.318(14)	Dy3-O15	2.302(13)
Dy1-O1	2.345(11)	Dy2-O1	2.411(9)	Dy3-O13	2.324(11)
Dy1-O11	2.220(10)	Dy2-O1T	2.416(10)	Dy3-O3	2.433(14)
Dy1-O13	2.321(13)	Dy2-O1V	2.395(10)	Dy3-O16	2.204(11)
Dy1-N11	2.499(14)	Dy2-O15	2.412(10)	Dy3-N15	2.481(15)
Dy1-N14	2.540(14)	Dy2-O12	2.190(16)	Dy3-N16	2.483(16)
[$Dy_4(HL^{H})(L^{H})_2(OH)_2(HCOO)(H_2O)_2(DMF)][Dy_4(HL^{H})(L^{H})_2(OH)_2(HCOO)(H_2O)(DMF)_2](NO_3)_2\cdot(H_2O)_{0.2}\cdot(DMF)_{2.75}$ (**2**)					
Dy11-Dy31	3.5150(7)	Dy31-O141	2.363(5)	Dy22-O12	2.360(5)
Dy11-Dy41	3.7675(7)	Dy31-O31	2.433(7)	Dy22-O122	2.264(5)
Dy11-O11	2.415(5)	Dy31-N161	2.511(8)	Dy22-N122	2.500(7)
Dy11-O21H	2.359(6)	Dy31-N151	2.489(7)	Dy22-O152	2.394(5)
Dy11-O11H	2.377(5)	Dy41-O21H	2.382(6)	Dy22-O12S	2.359(7)
Dy11-O21	2.308(6)	Dy41-O11W	2.337(6)	Dy22-O12F	2.324(7)
Dy11-O141	2.329(6)	Dy41-O11F	2.540(7)	Dy32-O142	2.352(6)
Dy11-O111	2.268(6)	Dy41-O21	2.240(7)	Dy32-O12H	2.330(5)
Dy11-N111	2.486(7)	Dy41-O21W	2.382(6)	Dy32-O12	2.424(5)
Dy11-N141	2.494(8)	Dy41-O131	2.233(6)	Dy32-O162	2.194(6)
Dy21-Dy11	3.5294(6)	Dy41-O21F	2.434(6)	Dy32-O152	2.335(6)
Dy21-Dy31	3.5727(7)	Dy41-N131	2.486(9)	Dy32-O32	2.447(7)
Dy21-O11	2.374(5)	Dy12-Dy11	3.5294(6)	Dy32-N162	2.518(8)
Dy21-O21H	2.420(5)	Dy12-Dy31	3.5727(7)	Dy32-N152	2.485(8)
Dy21-O11H	2.360(5)	Dy12-O11	2.374(5)	Dy42-O22H	2.387(6)
Dy21-O121	2.255(6)	Dy12-O21H	2.420(5)	Dy42-O12W	2.338(6)
Dy21-O151	2.385(6)	Dy12-O11H	2.360(5)	Dy42-O22S	2.317(7)
Dy21-O11F	2.396(6)	Dy12-O121	2.255(6)	Dy42-O132	2.269(6)
Dy21-O11S	2.365(6)	Dy12-O151	2.385(6)	Dy42-O22	2.248(6)

Table S5 continued.

Dy21-N121	2.511(7)	Dy12-O11F	2.396(6)	Dy42-O22F	2.387(8)
Dy31-O11	2.388(5)	Dy12-O11S	2.365(6)	Dy42-N132	2.469(8)
Dy31-O11H	2.352(5)	Dy12-N121	2.511(7)	Dy42-O12F	2.812(11)
Dy31-O151	2.337(6)	Dy12-N142	2.523(7)	Dy22-Dy32	3.5658(6)
Dy31-O161	2.191(6)	Dy22-O12H	2.381(5)	Dy22-O22H	2.413(6)
$[Dy_6(L^H)_4(OH)_4(NO_3)_2(H_2O)_2]\cdot(CH_3COCH_3)_{1.66}$ **(3)**					
Dy1-O1H	2.416(4)	Dy2-Dy1[1]	3.7919(7)	Dy3-Dy1	3.5233(5)
Dy1-O13[1]	2.363(4)	Dy2-Dy1	3.5407(5)	Dy3-O1H	2.350(4)
Dy1-O2[1]	2.296(4)	Dy2-O1H	2.333(4)	Dy3-O13[1]	2.313(4)
Dy1-O1	2.384(4)	Dy2-O1W	2.364(4)	Dy3-O1	2.413(4)
Dy1-O12	2.254(4)	Dy2-O2	2.265(3)	Dy3-O11	2.194(4)
Dy1-O2H	2.368(4)	Dy2-O1	2.446(4)	Dy3-O14	2.314(4)
Dy1-N12	2.502(5)	Dy2-N14	2.518(5)	Dy3-O25	2.480(4)
Dy1-N13[1]	2.503(5)	Dy2-O14	2.410(4)	Dy3-O15	2.500(4)
Dy2-Dy2[1]	3.9800(7)	Dy2-O2H	2.443(4)	Dy3-N15	2.896(5)
Dy2-Dy3	3.5845(8)	Dy2-O2H[1]	2.389(4)	Dy3-N11	2.490(5)
$[Dy_6(L^{p\text{-}OMe})_4(OH)_4(NO_3)_2(H_2O)_2]\cdot(H_2O)_{0.5}\cdot(CH_3COCH_3)_{2.33}$ **(4)**					
Dy1-Dy2[1]	3.5318(3)	Dy2-Dy2[1]	3.9618(5)	Dy3-O14[1]	2.328(3)
Dy1-Dy2	3.7989(3)	Dy2-Dy3[1]	3.5944(3)	Dy3-O2H[1]	2.351(3)
Dy1-Dy3	3.5038(3)	Dy2-O14	2.414(3)	Dy3-O13	2.305(3)
Dy1-O2	2.300(3)	Dy2-O2	2.261(3)	Dy3-O1	2.415(3)
Dy1-O2H[1]	2.417(3)	Dy2-O2H	2.327(3)	Dy3-O11	2.188(3)
Dy1-O1H	2.365(3)	Dy2-O1H[1]	2.420(3)	Dy3-O15	2.468(4)
Dy1-O13	2.351(3)	Dy2-O1H	2.391(3)	Dy3-N11	2.494(4)
Dy1-O1	2.368(3)	Dy2-O1[1]	2.460(3)	Dy3-O25	2.543(4)
Dy1-N12	2.522(4)	Dy2-O1W	2.3730(2)	Dy3-N15	2.935(5)
Dy1-O12	2.233(4)	Dy2-N14	2.510(4)		
Dy1-N13	2.543(6)				
Dy1-N13A	2.486(12)				

[1] 1-X,1-Y,1-Z

Table S5 continued.

$[Dy_2(L^{o\text{-}tBu})_2(EtOH)_2]\cdot EtOH$ (**5**)					
Dy1-Dy2	3.6817(6)	Dy1-N11	2.447(6)	Dy2-O1	2.323(5)
Dy1-O1	2.265(5)	Dy1-O1S	2.458(6)	Dy2-O2	2.245(5)
Dy1-O2	2.282(5)	Dy1-N13	2.472(6)	Dy2-O4S	2.409(6)
Dy1-O13	2.233(6)	Dy2-O14	2.193(5)	Dy2-N14	2.490(6)
Dy1-O11	2.181(5)	Dy2-O12	2.216(5)	Dy2-N12	2.466(6)
$[Dy_2(L^{o\text{-}tBu})_2THF_2]\cdot THF$ (**6**)					
Dy-Dy[1]	3.750(1)	Dy-O1	2.263(2)	Dy-N12	2.474(2)
Dy-O12	2.191 (2)	Dy-O1[1]	2.321(2)	Dy-O1S	2.488(2)
Dy-O11[1]	2.197(2)	Dy-N11[1]	2.457(2)		
$[NdDy(L^{o\text{-}tBu})_2(EtOH)_{2.69}]\cdot EtOH\cdot(H_2O)_{0.2}$ (**7**)					
Dy1-O2	2.281(4)	O24-Nd2	2.280(4)	O12-Dy2	2.312(5)
Dy1-O13	2.224(4)	O24-Dy2	2.280(4)	O7S-Nd2	2.640(7)
Dy1-O11	2.232(4)	O13-Nd1	2.224(4)	O1S-Nd1	2.406(4)
Dy1-O1	2.349(4)	O11-Nd1	2.232(4)	N13-Nd1	2.500(5)
Dy1-O1S	2.406(4)	O1-Nd2	2.320(4)	N14-Nd2	2.547(5)
Dy1-N13	2.500(5)	O1-Nd1	2.349(4)	N14-Dy2	2.547(5)
Dy1-N11	2.491(5)	O1-Dy2	2.320(4)	N11-Nd1	2.491(5)
O2-Nd2	2.390(4)	N12-Nd2	2.525(5)	O4S-Nd2	2.681(6)
O2-Nd1	2.281(4)	N12-Dy2	2.525(5)	O4SA-Dy2	2.265(14)
O2-Dy2	2.390(4)	O12-Nd2	2.312(5)		

[1]1-X,1-Y,1-Z

Table S5 continued.

$Et_3NH[Nd_7(L^{o-tBu})_4(OH)_6(NO_3)_4(EtOH)_4]\cdot(EtOH)_8$ (**7**)					
Nd1-Nd4^{1}	3.8407(5)	Nd2-O1H	2.489(4)	Nd3-O1	2.440(4)
Nd1-Nd4	3.8407(5)	Nd2-O1	2.324(5)	Nd3-O3H	2.501(5)
Nd1-Nd2	3.7056(5)	Nd2-O3H	2.458(4)	Nd3-O12	2.374(5)
Nd1-Nd2^{1}	3.7057(5)	Nd2-O4	2.590(5)	Nd3-O14	2.355(5)
Nd1-O1H^{1}	2.414(4)	Nd2-O11	2.235(5)	Nd3-N14	2.641(6)
Nd1-O1H	2.414(4)	Nd2-O1S	2.436(5)	Nd3-N12	2.629(6)
Nd1-O2H^{1}	2.472(5)	Nd2-O3	2.722(6)	Nd4-O1H^{1}	2.461(4)
Nd1-O2H	2.472(5)	Nd2-N11	2.528(6)	Nd4-O2H^{1}	2.463(4)
Nd1-O3H	2.417(4)	Nd3-Nd4	3.9741(6)	Nd4-O2	2.319(4)
Nd1-O3H^{1}	2.417(4)	Nd3-Nd2	4.0045(6)	Nd4-O13	2.263(5)
Nd1-O4^{1}	2.633(5)	Nd3-O2H^{1}	2.564(5)	Nd4-N13	2.532(5)
Nd1-O4	2.633(5)	Nd3-O2	2.423(4)	Nd4-O15	2.608(5)
				Nd4-O4S	2.458(5)
				Nd4-O25	2.627(6)

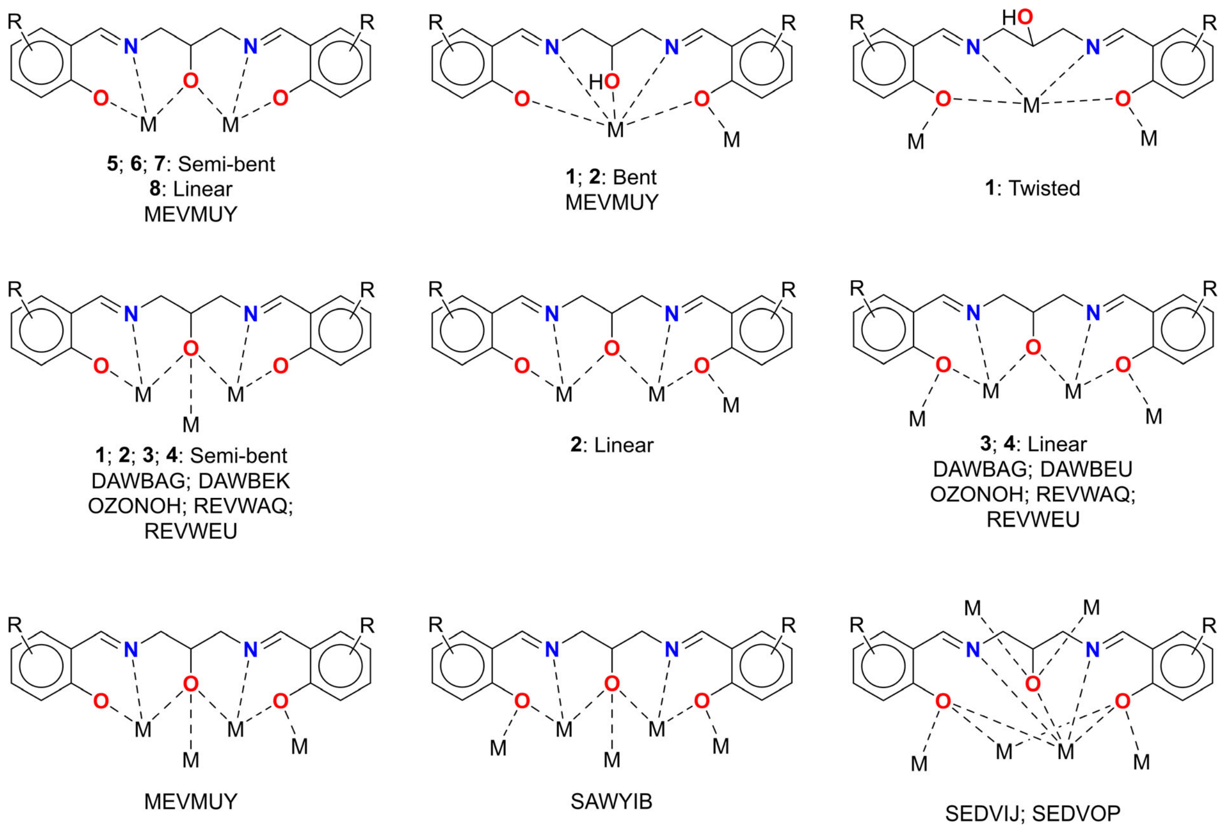

Figure S7. Schematic representation of the coordination modes of the H_3L^R (R=H; p-OMe; o-tBu) ligands observed in the lanthanide complexes **1-8** (see **Table 1**) and in related complexes from the CCDC (refcodes reported under each scheme, see **Table S15**). For the complexes characterized in this work, the ligand conformations are also described.

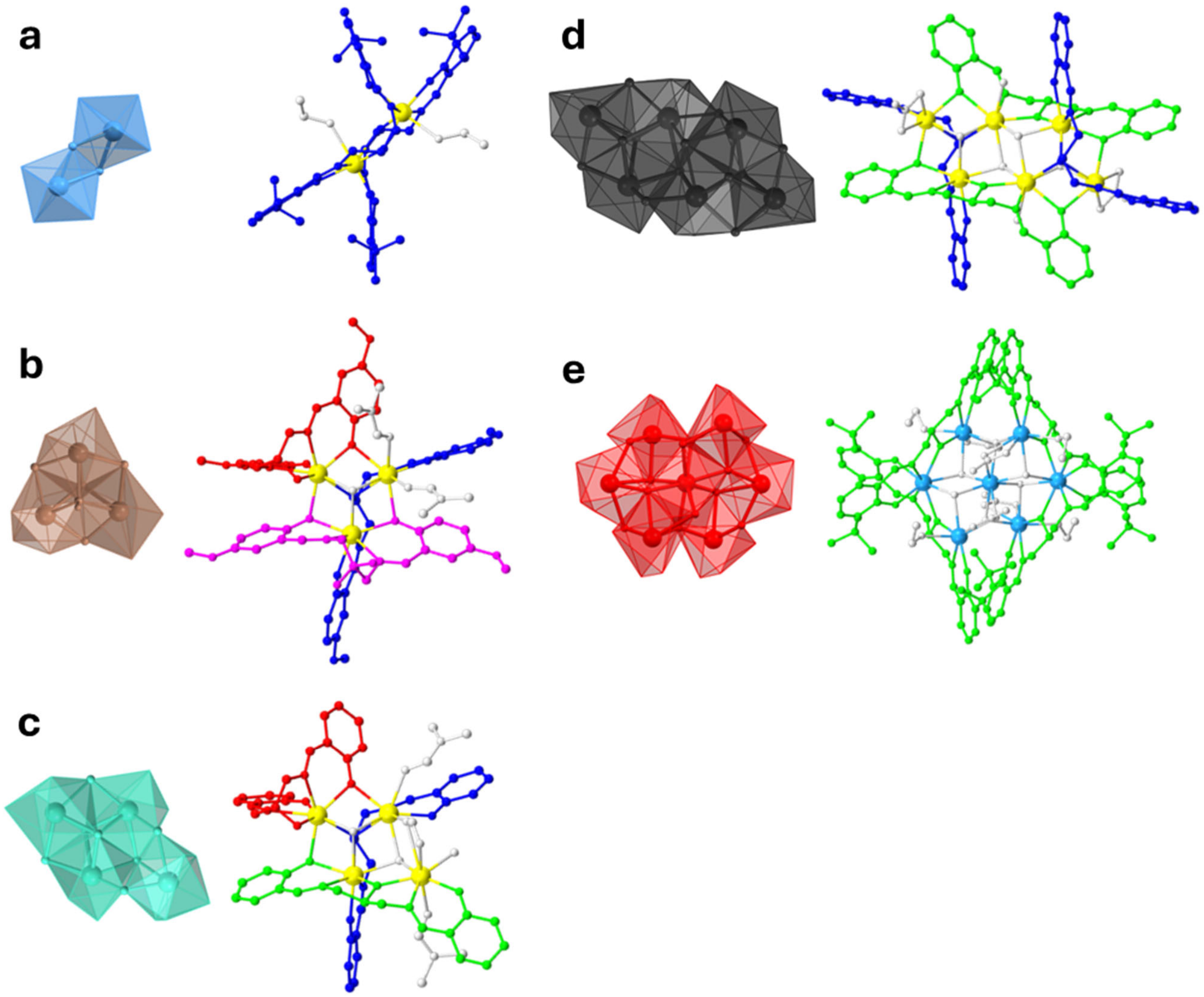

Figure S8. Crystal structure highlighting the metal coordination polyhedral of a) complexes **5** (dinuclear, in light blue), b) complex **1** (trinuclear, in brown), c) complexes **2** (tetranuclear, in emerald), d) complexes **3** (hexanuclear, in black), e) complexes **8** (heptanuclear, in red). Oxygen atoms bridging between multiple metal centers are reported as balls and sticks. The different conformations of the ligands are highlighted using the color scheme reported in **Figure 2**: bent in red, semi-bend in blue, linear in green and twisted in magenta. Ancillary ligands, solvent molecules and hydroxide ions are reported in white; hydrogen atoms and non-coordinated solvents were omitted for clarity.

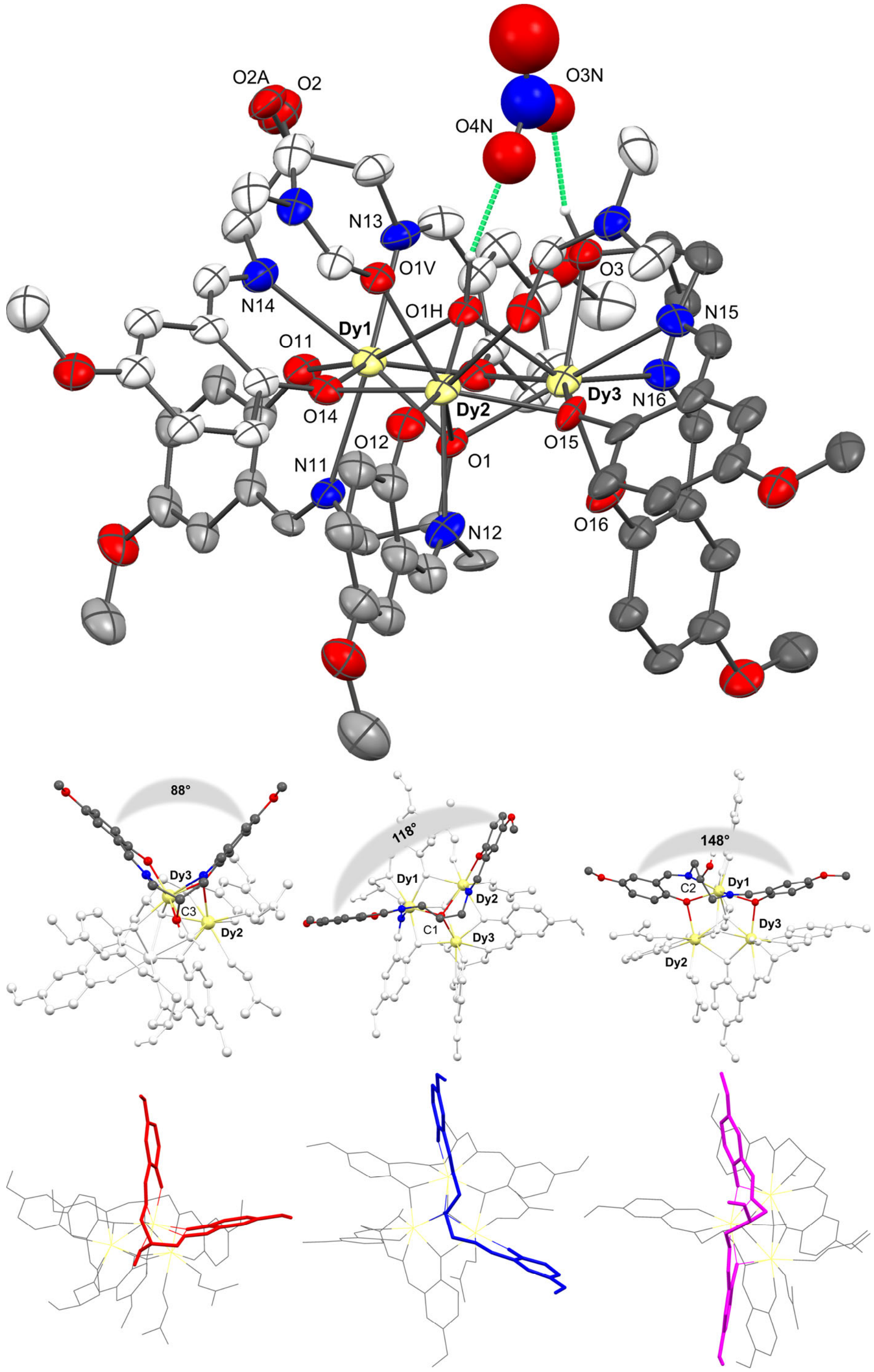

Figure S9. Above, representation of the structure of the trinuclear complex $[Dy_3(HL^{p\text{-}OMe})_2(L^{p\text{-}OMe})(OH)(DMF)_2]NO_3{\cdot}(DMF)_2$ (**1**). Solvent of crystallization and the hydrogen atoms (except for the hydroxylic groups O2/O2A, O3 and the hydroxide ion O1H) were removed for clarity. Hydrogen bonds are represented with green dashed lines. Thermal ellipsoids were depicted at the 30% probability level. Dy (yellow), N (blue), O (red), C (dark grey, grey and light grey), H (white). Below, depiction of the conformations adopted by the $L^{p\text{-}OMe}$ and $HL^{p\text{-}OMe}$ ligands.

The ligand containing C3 (μ_2-k^{6}O16,N16,O3,N15,O15:O15) adopts a bent conformation, the ligand containing C1 (μ_3-k^{7}O11,N11,O1:O1:O1,N12,O12) adopts a semi-bent conformation whereas the ligand containing C2 (μ_3-k^{6}O14:O14,N14,N13,O13:O13) adopts an twisted conformation. The angles were calculated from the mean planes passing through the two aromatic rings for each ligand.

The central hydroxylic oxygen atom (O2A) in the grey ligand does not interact with any metal centre and its position is disordered over two sites with 0.56 and 0.44 site occupancy factors. The Dy-$O_{phenolic}$ (O15, O14, O13) bond distances [2,331(3)-2,411(10) Å] are longer than the remaining Dy-$O_{phenolic}$ (O16, O11, O12) ones [2,200(6)-2,220(10) Å] due to the fact that the former are shared between 2 metal centres while the latter are coordinated to only one Dy atom. Moreover, the Dy-O3 bond distance 2,443(4) Å is longer than the Dy-O1 ones [2,355(1)-2,411(9) Å] due to O3 being protonated while O1 is being shared between all three metal centres. The Dy-N bond distances vary in the range [2.491(5)-2.550(8) Å].

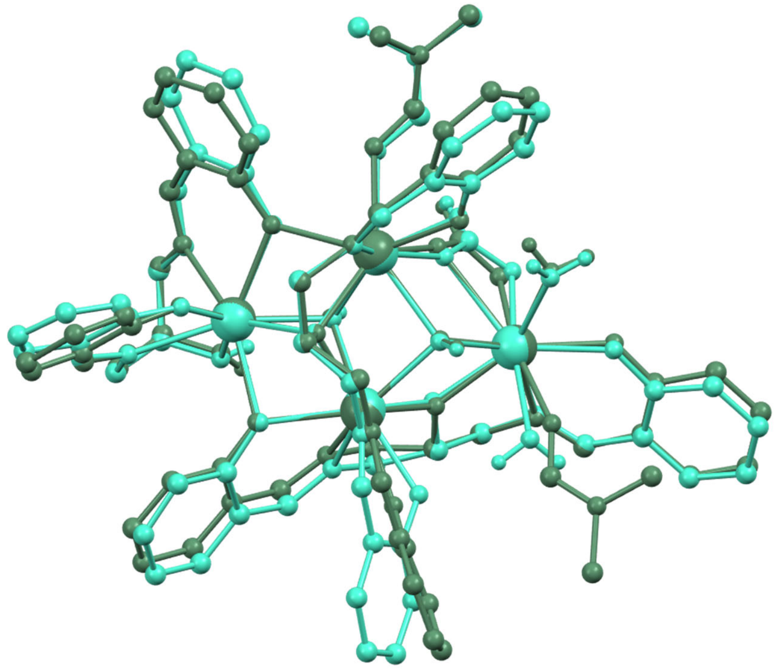

Figure S10. Overlay of the two complex entities present in the asymmetric unit of compound **2**, highlighting the equivalent coordination of the metals. The first complex is shown in emerald, while the second one is shown in olive.

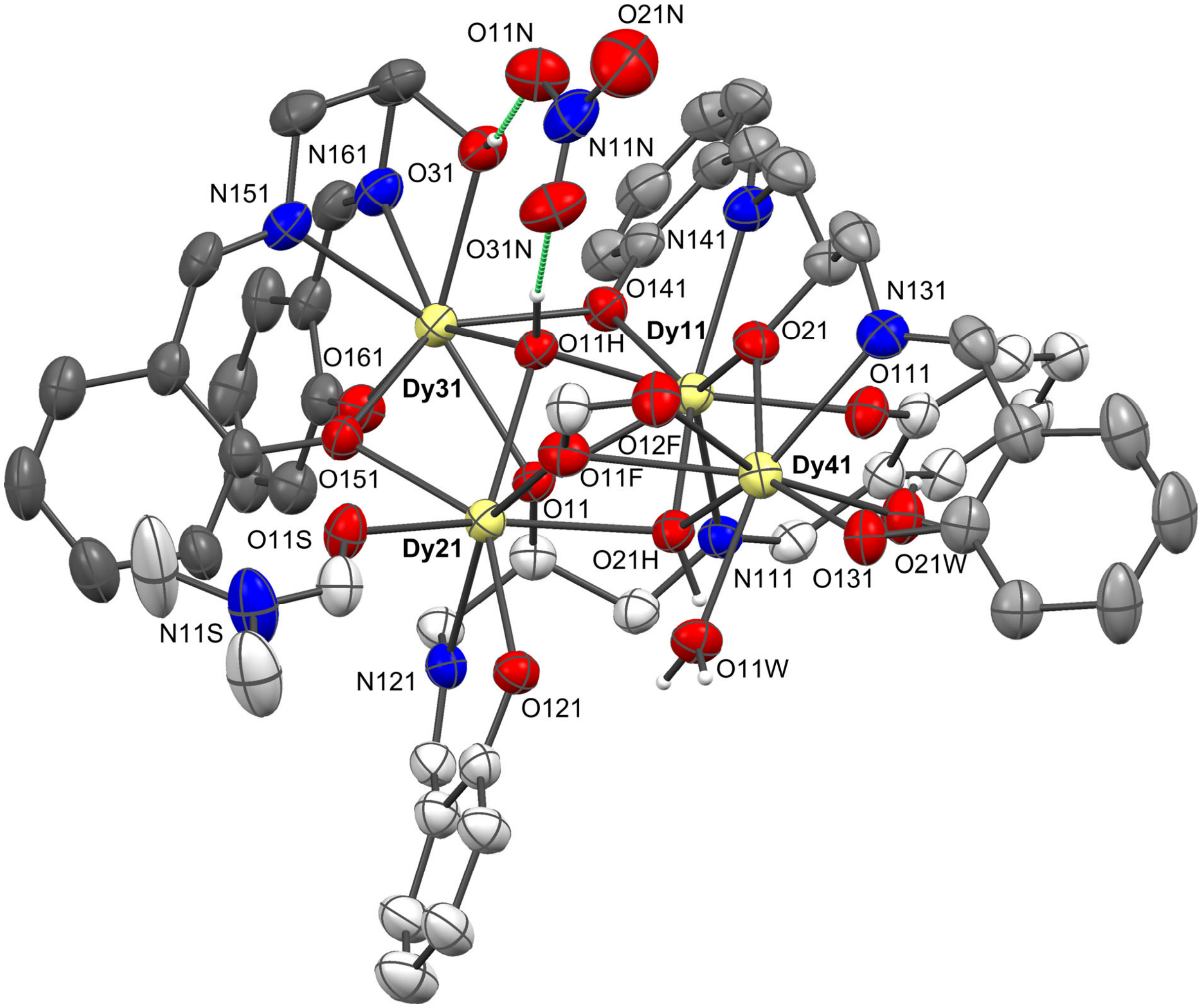

Figure S11. Representation of the structure of the first unit of the tetranuclear complex $[Dy_4(HL^H)(L^H)_2(OH)_2(HCOO)(H_2O)_2(DMF)][Dy_4(HL^H)(L^H)_2(OH)_2(HCOO)(H_2O)(DMF)_2](NO_3)_2{\cdot}(H_2O)_{0.2}{\cdot}(DMF)_{2.75}$ (**2**). Solvent of crystallization and the hydrogen atoms (except for the hydroxide ions O11H, O21H, the water molecules O11W, O21W and the hydroxyl group O31) were removed for clarity. Hydrogen bonds are represented with green dashed lines. Thermal ellipsoids were depicted at the 30% probability level. Dy (yellow), N (blue) and O (red) and C (dark grey, grey and light grey), H (white).

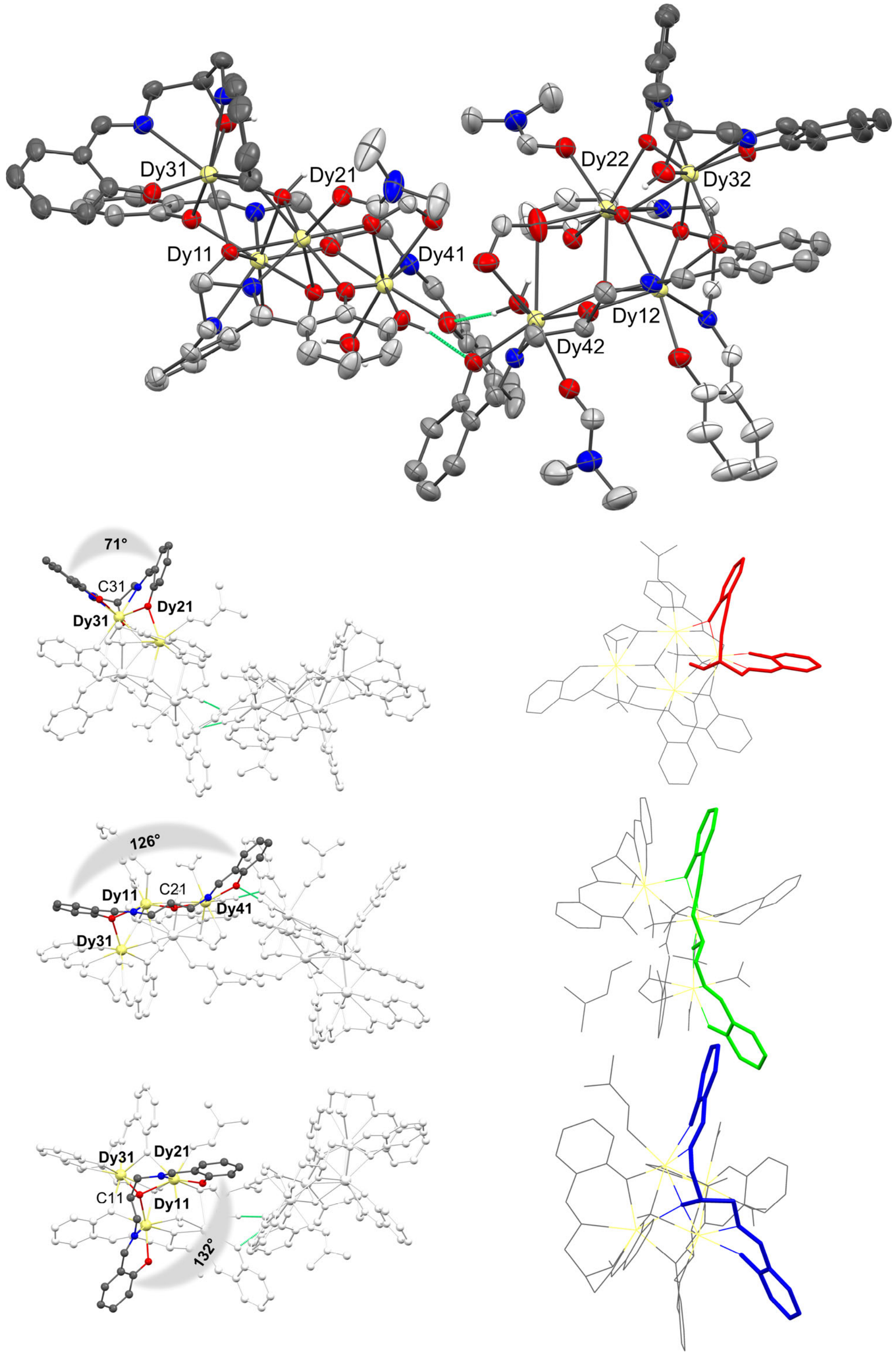

Figure S12. Above, representation of the structure of the tetranuclear complex $[Dy_4(HL^H)(L^H)_2(OH)_2(HCOO)(H_2O)_2(DMF)]$ $[Dy_4(HL^H)(L^H)_2(OH)_2(HCOO)(H_2O)(DMF)_2](NO_3)_2{\cdot}(H_2O)_{0.2}{\cdot}(DMF)_{2.75}$ (**2**). Solvent of crystallization and the hydrogen atoms (except for the hydroxide ions O11H, O21H, the water molecules O11W, O21W and the hydroxyl group O31) were removed for clarity. Hydrogen bonds are represented with green dashed lines. Thermal ellipsoids were depicted at the 30% probability level. Dy (yellow), N (blue) and O (red) and C (dark grey, grey and light grey), H (white). Below, depiction of the conformations adopted by the L and HL ligands in the first unit.

The ligands containing C31 (μ_2-k^{6}O161,N161,O31,N151,O151:O151) adopts a bent conformation, the ligands containing C11 (μ_3-k^{7}O111,N111,O11:O11:O11,N121,O121) adopts a semi-bent conformation, whereas the ligands containing C21 (μ_3-k^{7}O131,N131,O21:O21,N141,O141:O141) adopts a linear conformation. The angles were calculated from the mean planes passing through the two aromatic rings for each ligand.

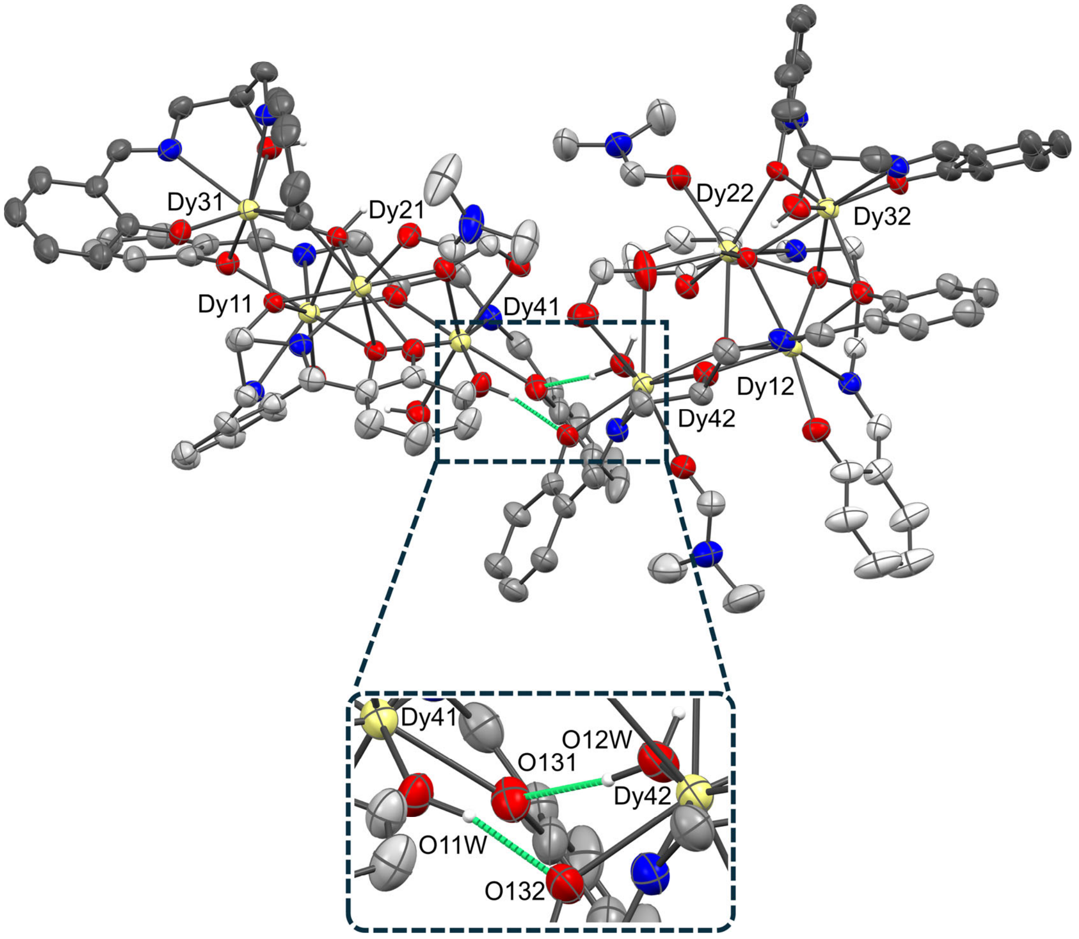

Figure S13. Representation of the complex (**2**) highlighting with green dashed lines the hydrogen bonding between the two distinct molecular entities. Solvent of crystallization and the hydrogen atoms were removed for clarity (except for the water molecules O11W, O12W). Thermal ellipsoids were depicted at the 30% probability level. Dy (yellow), N (blue) and O (red) and C (dark grey, grey and light grey), H (white).

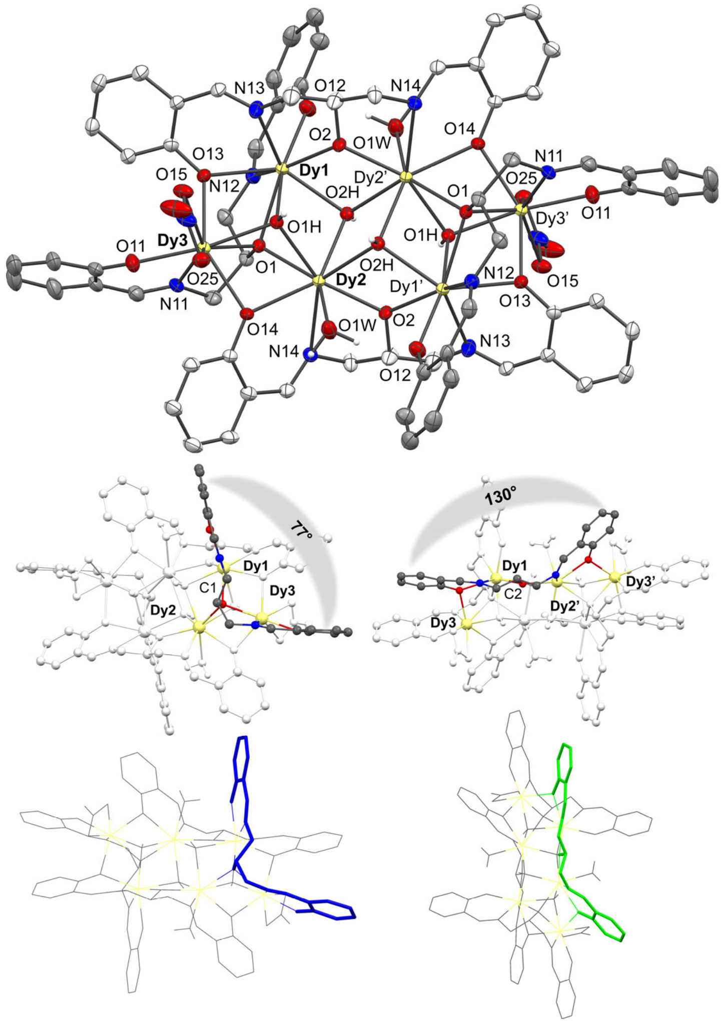

Figure S14. Above, representation of the structure of the hexanuclear complex $[Dy_6(L^H)_4(OH)_4(NO_3)_2(H_2O)_2]\cdot(CH_3COCH_3)_{1.66}$ (**3**). Solvent of crystallization and hydrogen atoms (except for the hydroxide ions O1H, O2H and the water molecules O1W) were removed for clarity. Symmetry operator (1-x, 1-y, 1-z) generates equivalent atoms marked with ‘. Dy (yellow), N (blue), O (red) and C (grey and light grey), H (white). Thermal ellipsoids were depicted at the 30% probability level. Below, depiction of the conformations adopted by the L ligands.

The ligand containing C1 (μ_3-k^7O11,N11,O1:O1:O1,N12,O12) adopts a semi-bent conformation whereas the ligand containing C2 (μ_3-k^8O14:O14,N14,O2:O2,N13,O13:O13) adopts a linear conformation. The angles were calculated from the mean planes passing through the two aromatic rings for each ligand.

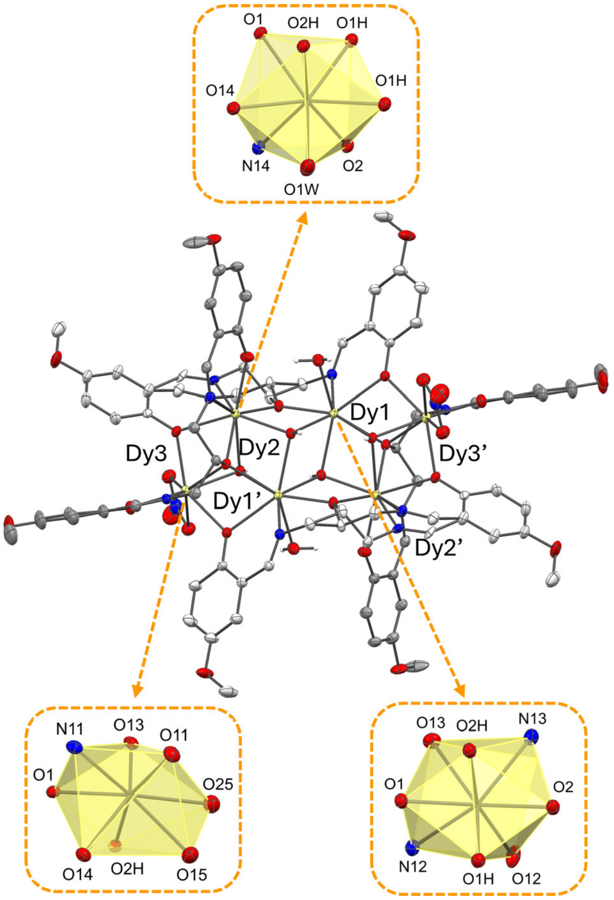

Figure S15. Molecular structure of the hexanuclear complex $[Dy_6(L^{p\text{-}OMe})_4(OH)_4(NO_3)_2(H_2O)_2]\cdot(H_2O)_{0.5}\cdot(CH_3COCH_3)_{2.33}$ (**4**). Solvent of crystallization and the hydrogen atoms (except for the hydroxide ions O1H, O2H and the water molecule O1W) were removed for clarity. Thermal ellipsoids were depicted at the 30% probability level. The bicapped trigonal prismatic geometries of Dy1, Dy2 and Dy3 are highlighted where the capping positions are represented by O1 and O2, O14 and O1H, O1 and O25, respectively. Symmetry operator (1-x, 1-y, 1-z) generates equivalent atoms marked with ‘. Dy (pale-yellow), N (blue), O (red), C (grey and light grey), H (white).

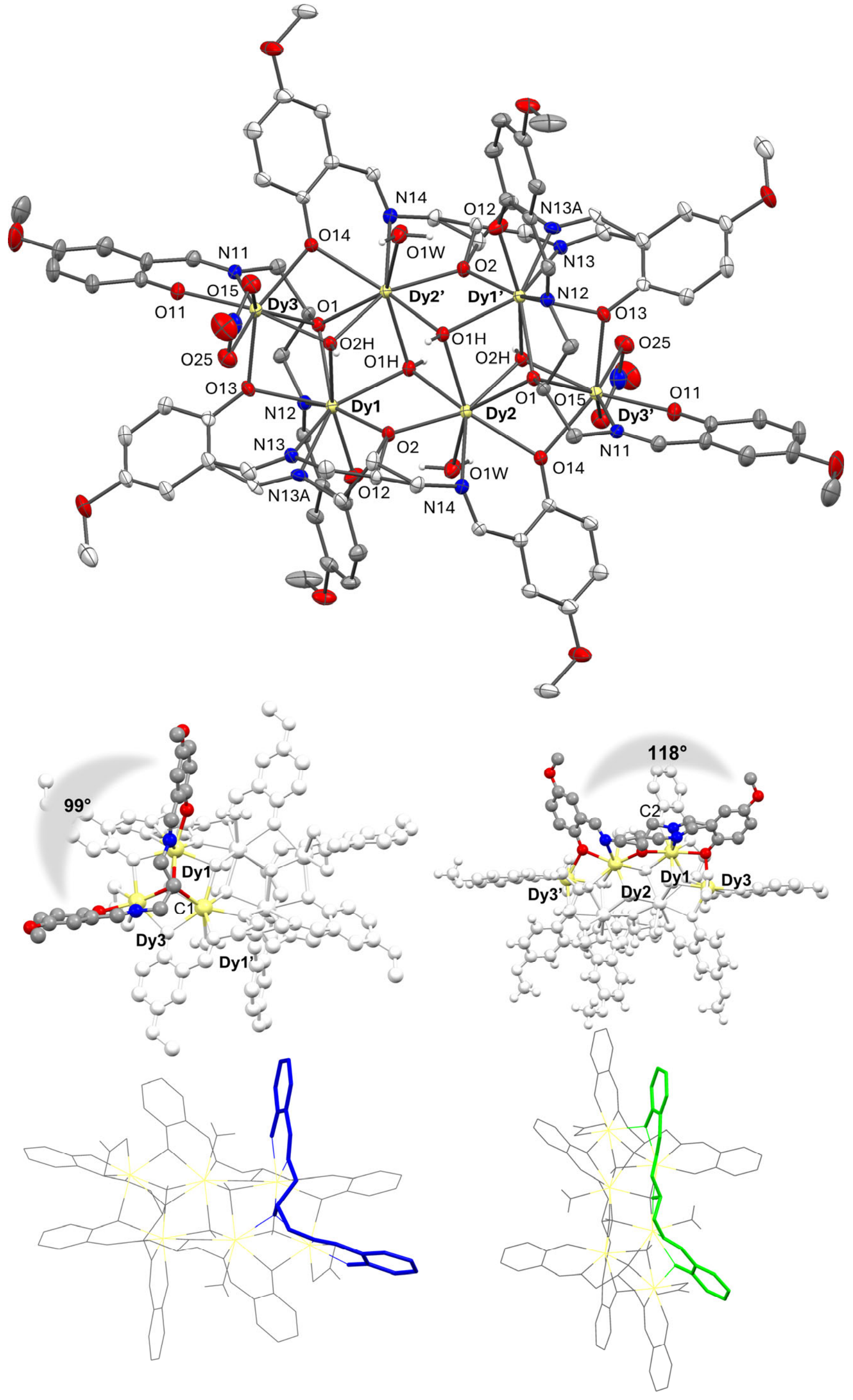

Figure S16. Above, representation of the structure of the hexanuclear complex [Dy$_6$(L$^{p\text{-}OMe}$)$_4$(OH)$_4$(NO$_3$)$_2$(H$_2$O)$_2$]·(H$_2$O)$_{0.5}$·(CH$_3$COCH$_3$)$_{2.33}$ (**4**). Solvent of crystallization and hydrogen atoms (except for the hydroxide ions O1H, O2H and the water molecules O1W) were removed for clarity. Thermal ellipsoids were depicted at the 30% probability level. Symmetry operator (1-x, 1-y, 1-z) generates equivalent atoms marked with ‘. Dy (yellow), N (blue), O (red) and C (grey and light grey), H (white). Below, depiction of the conformations adopted by the L$^{p\text{-}OMe}$ ligands.

The ligand containing C1 (μ$_3$-k^{7}O11,N11,O1:O1:O1,N12,O12) adopts a semi-bent conformation whereas the ligand containing C2 (μ$_4$-k^{8}O14:O14,N14,O2:O2,N13,O13:O13) adopts a linear conformation. The angles were calculated from the mean planes passing through the two aromatic rings for each ligand.

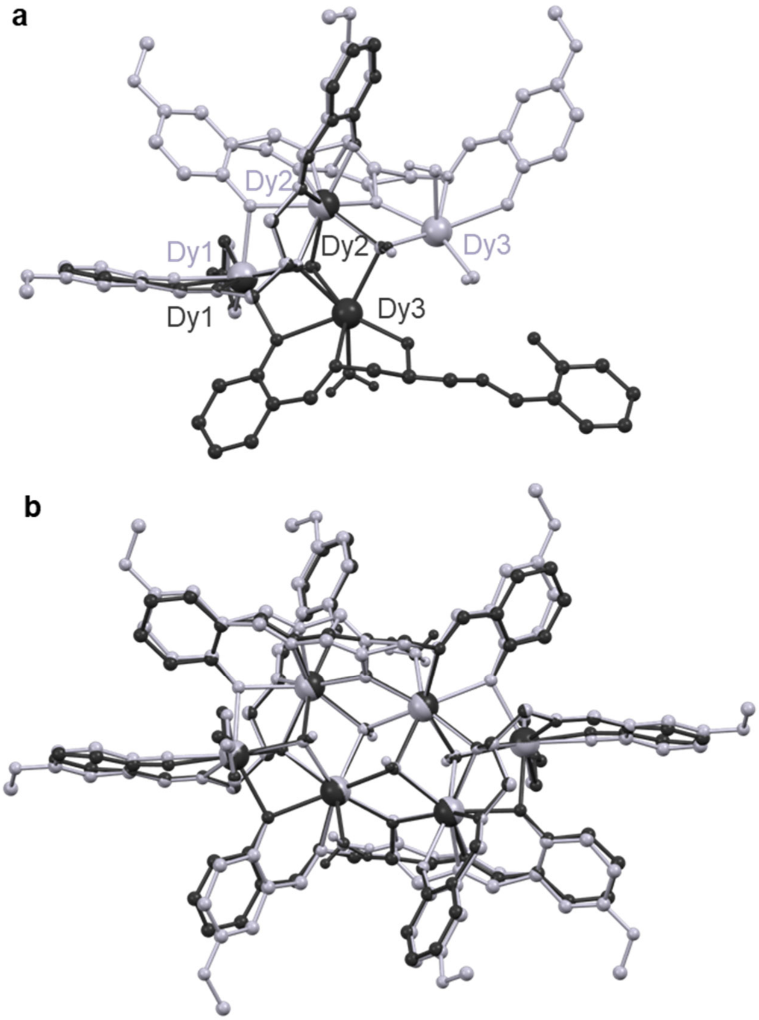

Figure S17. Overlay of the crystal structure of compound **3**, shown in black, and of compound **4**, shown in grey, highlighting the equivalent coordination of the metals.

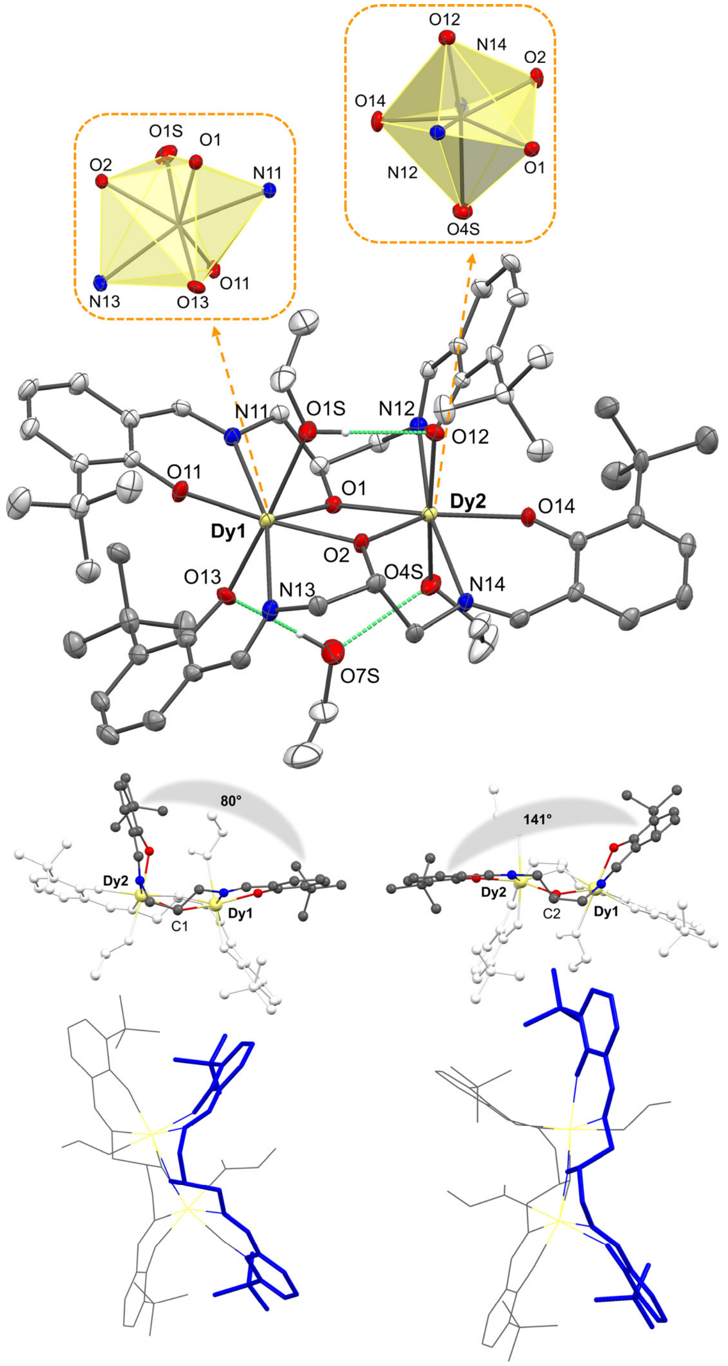

Figure S18. Molecular structure of the dinuclear complex [$Dy_2(L^{o\text{-}tBu})_2(EtOH)_2$]·EtOH (**5**). Solvent of crystallization and hydrogen atoms were removed for clarity. Hydrogen bonds are represented with green dashed lines. Thermal ellipsoids were depicted at the 30% probability level. The capped trigonal prismatic geometry of Dy1 and the capped octahedral geometry of Dy2 are highlighted and N11 and O2 respectively represent the capping position. Dy (pale-yellow), N (blue), O (red), C (grey and light grey).. Below, depiction of the semi-bent conformations adopted by the $L^{o\text{-}tBu}$ ligands (μ_2-k^{6}O12,N12,O1:O1,N11,O11 and μ_2-k^{6}O14,N14,O2:O2,N13,O13). The angles were calculated from the mean planes passing through the two aromatic rings for each ligand.

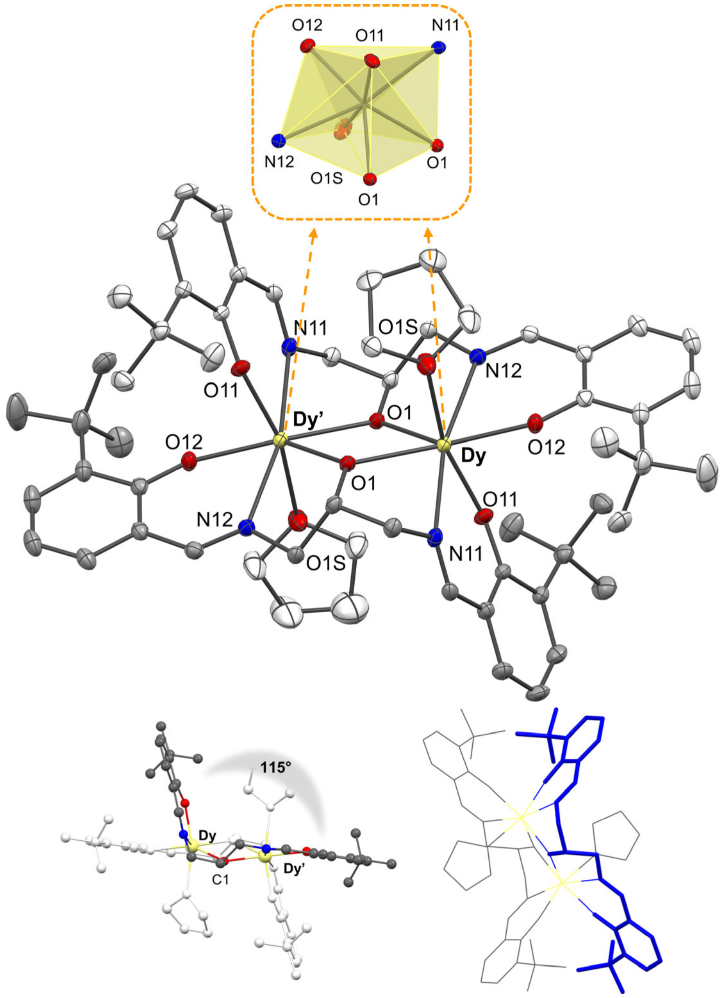

Figure S19. Molecular structure of the dinuclear complex $[Dy_2(L^{o\text{-}tBu})_2(THF)_2]$·THF (**6**). Solvent of crystallization and hydrogen atoms were removed for clarity. Thermal ellipsoids were depicted at the 30% probability level. The capped trigonal prismatic geometry of Dy is highlighted where N12 represents the capping position. Symmetry operator (-x, -y, -z) generates equivalent atoms marked with '. Dy (pale-yellow), N (blue), O (red), C (grey and light grey). Below, depiction of the semi-bent conformation adopted by the $L^{o\text{-}tBu}$ ligand molecule (μ_2-k^{6}O11,N11,O1:O1,N12,O12) in the complex. The angle of 115 deg was calculated from the mean planes passing through the two aromatic rings.

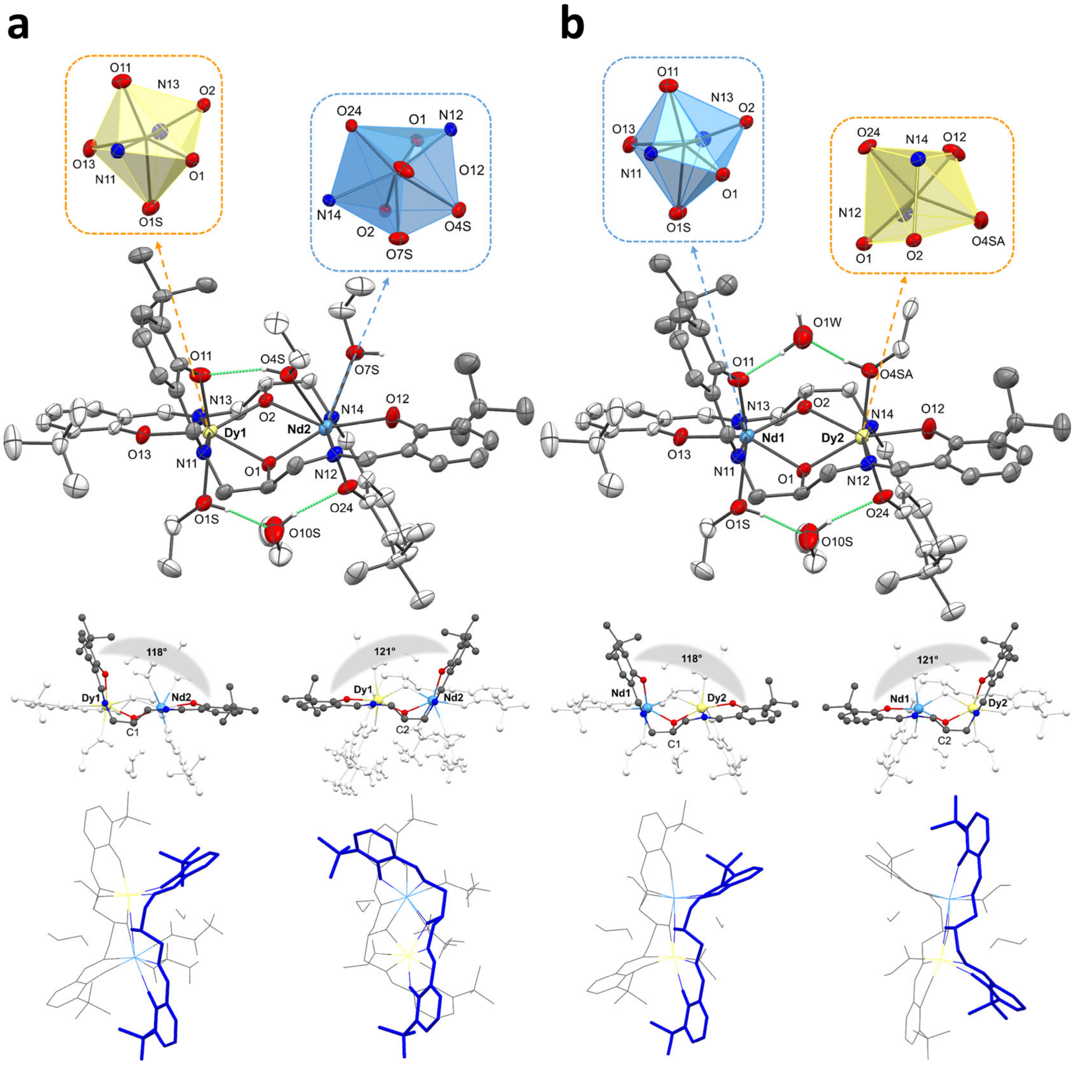

Figure S20. Above, representation of the structure of the heteronuclear complex [NdDy($L^{o\text{-}tBu}$)$_2$(EtOH)$_{2.69}$]·EtOH·(H_2O)$_{0.2}$ (**7**). a) Principal component disorder with Dy1, Nd2, O7S and O4S refined with site occupancy factors of 0.69 and b) Minor component disorder with Nd1, Dy2 and O4SA refined with site occupancy factors of 0.31. Hydrogen atoms were removed for clarity. Hydrogen bonds are represented with green dashed lines. Thermal ellipsoids were depicted at the 30% probability level. The capped octahedral geometry of Dy1 and Nd1 (O2 represents the capping position), the trigonal dodecahedral geometry of Nd2 and the capping trigonal prismatic geometry of Dy2 (where O4SA represents capping positions) are highlighted. Nd (light blue), Dy (yellow), N (blue), O (red) and C (grey and light grey), H (white). Below, depiction of the conformations adopted by the $L^{o\text{-}tBu}$ ligands. The ligand containing C1 (μ_2-k^6O11,N11,O1:O1,N12,O12) adopts a semi-bent conformation as well as the ligand containing C2 (μ_2-k^6O13,N13,O2:O2,N14,O24). The angles were calculated from the mean planes passing through the two aromatic rings for each ligand.

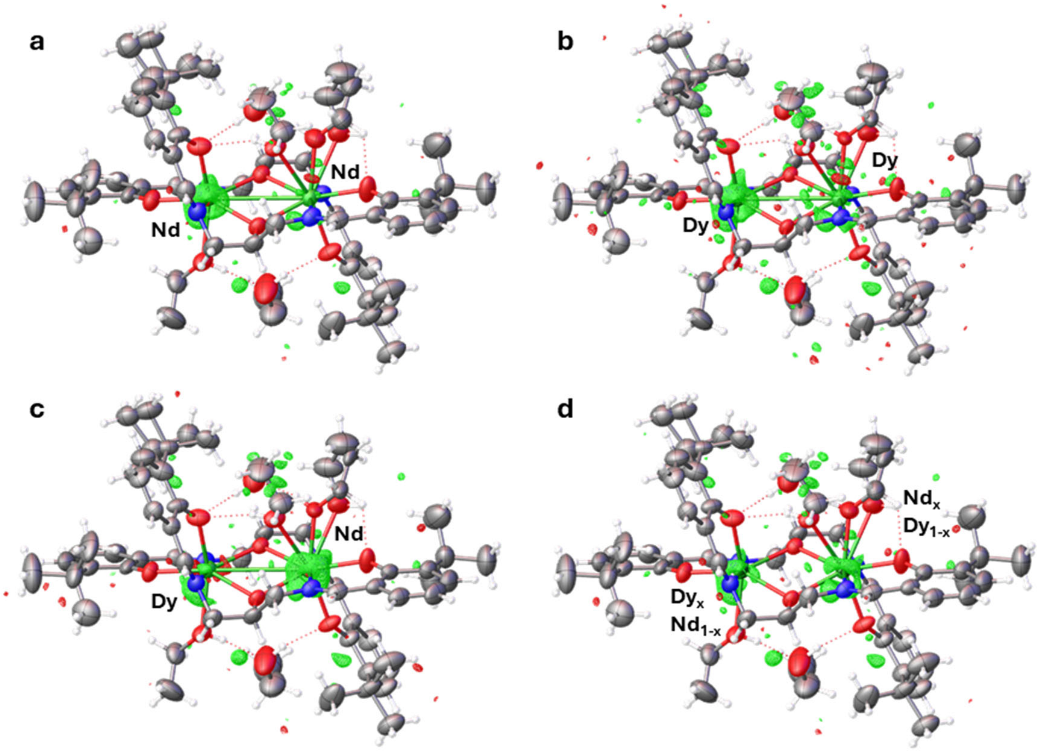

Figure S21. Electronic density difference map (cut at 0.9 e/Å^3) for different refinements of the structure of compound **7**: a) refinement with one Nd atom for each metal site; b) refinement with one Dy atom for each metal site; c) refinement with one Dy atom in the first metal site and one Nd atom in the second; d) refinement with Nd and Dy disordered over the two metal sites and refined with free variables. The first metal site was refined with site occupancies of 0.69 and 0.31 for Dy and Nd, respectively, and the second metal site was refined with site occupancies of 0.31 and 0.69 for Dy and Nd, respectively. Negative residual electronic density is reported in red while positive residual electronic density is indicated in green.

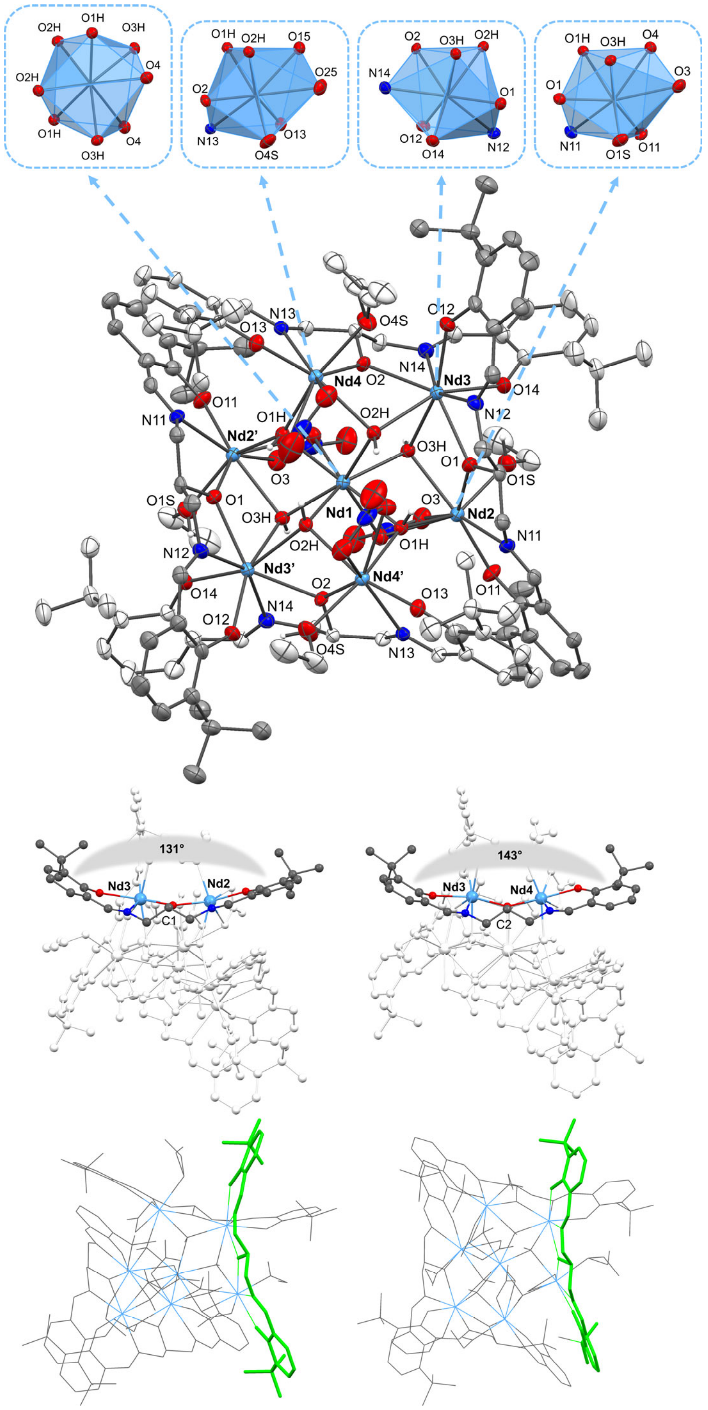

Figure S22. Above, representation of the structure of the heptanuclear complex $Et_3NH[Nd_7(L^{o\text{-}tBu})_4(OH)_6(NO_3)_4(EtOH)_4]\cdot(EtOH)_8$ (**8**). Solvent of crystallization and hydrogen atoms (except for the hydroxide ions O1H, O2H, O3H and the ethanol molecules O1S, O4S) were removed for clarity. Thermal ellipsoids were depicted at the 30% probability level. The square antiprismatic geometry of Nd1 and the bicapped trigonal prismatic geometries of Nd2, Nd3 and Nd4 are highlighted (O1 and O3, N14 and O1, O2 and O25 represent the capping positions). Symmetry operator (2-fold symmetry axis) generates equivalent atoms marked with ‘. Nd (light blue), N (blue), O (red) and C (grey and light grey), H (white). Below, depiction of the conformations adopted by the $L^{o\text{-}tBu}$ ligands. The ligand containing C1 (μ_2-k^6O11,N11,O1:O1,N12,O12) adopts a linear conformation as well as the ligand containing C2 (μ_2-k^6O14,N14,O2:O2,N13,O13). The angles were calculated from the mean planes passing through the two aromatic rings for each ligand.

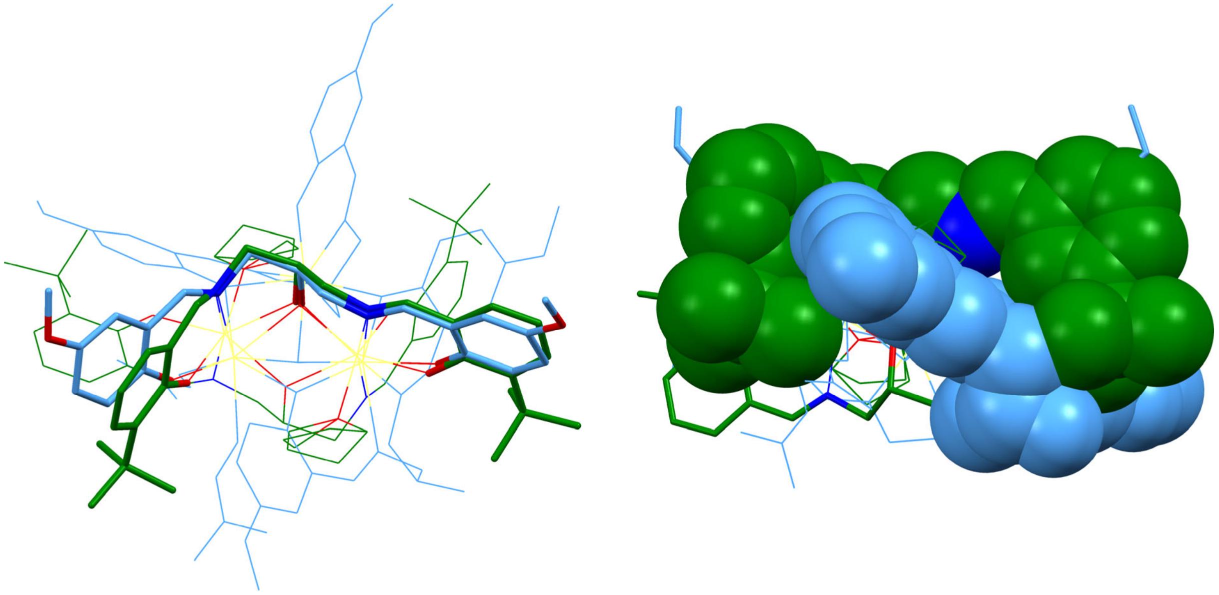

Figure S23. Left, overlay of the semibent ligands from the crystal structure of complex **1** (light blue) and complex **5** (green). Right, space filling representation showing the twisted ligand in the crystal structure of complex **1** (light blue) and the semi-bent ligand in complex **5** (green). The distances between the tert-butyl group of **5** and the backbone carbon atoms of **1** are 2.13 Å and 2.93 Å. Hydrogen atoms were omitted for clarity.

Table S6. Above, summary of the calculated angles for the H_3L^R ligands in compounds **1-8**. Below, schematic representation of the ϕ angle between the three carbon atoms highlighted in yellow.

Compound	Central carbon atom of the ligand	Angle between the phenolic rings	Angle ϕ (C-C-C)	Conformation	Bridging μ	Average μ
**1**	C3	88°	83°	Bent	2	2.7
	C1	118°	123°	Semi-bent	3	
	C2	148°	110°	Twisted	3	
**2**	C31	71°	80°	Bent	2	2.7
	C21	126°	162°	Linear	3	
	C11	132°	122°	Semi-bent	3	
	C32	69°	77°	Bent	2	
	C12	70°	118°	Semi-bent	3	
	C22	133°	164°	Linear	3	
**3**	C1	77°	114°	Semi-bent	3	3.5
	C2	130°	156°	Linear	4	
	$C1^1$	77°	114°	Semi-bent	3	
	$C2^1$	130°	156°	Linear	4	
**4**	C1	99°	114°	Semi-bent	3	3.5
	C2	118°	142°	Linear	4	
	$C1^1$	99°	114°	Semi-bent	3	
	$C2^1$	118°	142°	Linear	4	
**5**	C1	80°	108°	Semi-bent	2	2
	C2	141°	131°	Semi-Bent	2	
**6**	C1	115°	116°	Semi-bent	2	2
	$C1^1$	115°	116°	Semi-bent	2	
**7**	C1	118°	113°	Semi-bent	2	2
	C2	121°	120°	Semi-bent	2	
**8**	C1	131°	162°	Linear	2	2
	C2	143°	163°	Linear	2	
	$C1^1$	131°	162°	Linear	2	
	$C2^1$	143°	163°	Linear	2	

^{1}1-X,1-Y,1-Z

The angle between the phenolic rings was determined considering the planes defined by all the carbon atoms of the rings.

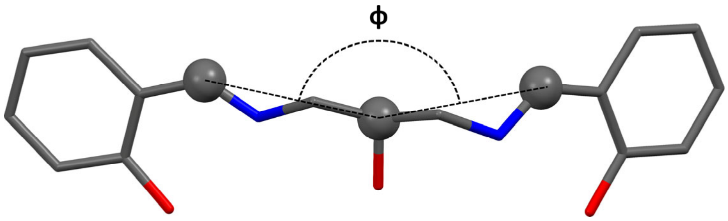

Figure S24. Molecular scheme on the calculation of the ϕ angle between the two carbons involved in the iminic double bonds and the central carbon atom. The ligand represented is taken from the structure of compound **5.**

Table S7. Lanthanide geometry analysis using the SHAPE software for complexes **5** and **6**.

Compound	ATOM LABEL	CTPR-7	COC-7	PBPY-7
**5**	Dy1	**2.292**	2.944	4.546
	Dy2	2.723	**2.538**	4.542
**6**	Dy	**2.719**	2.721	5.222

CTPR-7: Capped trigonal prism (C_{2v} symmetry); COC-7: Capped octahedron (C_{3v} symmetry); PBPY-7: Pentagonal bipyramid (D_{5h} symmetry).[1]

Table S8. Lanthanide geometry analysis using the SHAPE software for complexes **2**-**5** and **7**.

Compound	ATOM LABEL	BTPR-8	TDD-8	SAPR-8
**1**	Dy1	2.069	**1.544**	4.341
	Dy2	**1.239**	2.788	3.586
	Dy3	2.550	**1.364**	3.242
**2**	Dy11	**1.358**	2.604	4.152
	Dy21	2.141	**1.387**	2.206
	Dy31	2.478	**1.189**	3.246
	Dy41	3.317	**2.440**	3.608
	Dy12	**1.871**	2.981	5.366
	Dy22	1.539	1.817	**1.430**
	Dy32	2.456	**1.175**	3.118
	Dy42	4.028	**2.981**	5.366
**3**	Dy1	**1.168**	2.767	3.554
	Dy2	**1.705**	1.846	2.469
	Dy3	**2.421**	3.471	5.060
**4**	Dy1	**1.355**	3.160	3.809
	Dy2	**1.725**	2.015	2.520
	Dy3	**1.942**	3.831	4.629
**8**	Nd1	2.844	1.463	**1.226**
	Nd2	**3.373**	5.296	6.438
	Nd3	**2.649**	3.304	3.324
	Nd4	**3.536**	4.218	4.671

BTPR-8: Biaugmented trigonal prism (C_{2v} symmetry); TDD-8: Triangular dodecahedron (D_{2d} symmetry); SAPR-8: Square antiprism (D_{4d} symmetry).[2]

Table S9. Lanthanide geometry analysis using the SHAPE software for complexes **7**.

ATOM LABEL	CTPR-7	COC-7	PBPY-7	BTPR-8	TDD-8	SAPR-8
Dy1	2.545	**2.226**	5.533	-	-	-
Dy2	**2.846**	3.790	6.989	-	-	-
Nd1	2.545	**2.226**	5.533	-	-	-
Nd2	**-**	-	-	4.008	**2.690**	2.827

CTPR-7: Capped trigonal prism (C_{2v} symmetry); COC-7: Capped octahedron (C_{3v} symmetry); PBPY-7: Pentagonal bipyramid (D_{5h} symmetry); BTPR-8: Biaugmented trigonal prism (C_{2v} symmetry); TDD-8: Triangular dodecahedron (D_{2d} symmetry); SAPR-8: Square antiprism (D_{4d} symmetry).

Table S10. Selected geometric parameters for complex **5**.

distance	length (Å)
$M-(\mu_2-O^-)$	2.28 (±0.03)
Dy-Dy	3.6817 (±0.0006)

Table S11. Selected geometric parameters for complexes **1**-**3**.

	distance	**1** (trinuclear)	**2** (tetranuclear)	**3** (hexanuclear)
length (Å)	$M-(\mu_3-OH^-)_{prim}$	2.39 (±0.05)	2.37 (±0.03)	2.37 (±0.04)
	$M-(\mu_3-OR^-)_{prim}$	2.38 (±0.03)	2.39 (±0.02)	2.41 (±0.03)
	$M-(\mu_2-O^-)_{prim}$	2.35 (±0.04)	2.37 (±0.03)	2.37 (±0.05)
	$(Dy-Dy)_{prim}$	3.56 (±0.01)	3.55 (±0.03)	3.55 (±0.03)
	$M-(\mu_3-OH^-)_{sec}$	-	2.39 (±0.03)	2.40 (±0.03)
	$M-(\mu_2-O^-)_{sec}$	-	2.4 (±0.1)	2.33 (±0.07)
	$(Dy-Dy)_{sec}$	-	3.8 (±0.2)	3.8 (±0.2)
Metals MP length (Å)	$(\mu_3-OH^-)-MP_{prim}$	1.21	1.20 ±(0.01)	1.18
	$(\mu_3-OR^-)-MP_{prim}$	-1.20	-1.24 ±(0.01)	-1.28
	$(\mu_2-O^-)-MP_{prim}$	-0.29 (±0.04)	-0.2 ±(0.2)	-0.3 (±0.04)
	$(\mu_3-OH^-)-MP_{sec}$	-	-0.92 ±(0.02)	-0.996
	$(\mu_2-O^-)-MP_{sec}$	-	1.2 ±(0.3)	1.2 (±0.2)
	$Dy1-MP_{tot}$	-	-0.49 ±(0.01)	0.26
	$Dy2-MP_{tot}$	-	0.41 ±(0.01)	-0.46
	$Dy3-MP_{tot}$	-	-0.39 ±(0.01)	-0.26
	$Dy4-MP_{tot}$	-	0.48 ±(0.02)	-
Angles (°)	$(Dy-Dy-Dy)_{prim}$	59.9 – 60.3	59.1 – 61.0	59.3 – 61.0
	$(Dy-Dy-Dy)_{sec}$	-	53.5 – 67.3	54.1 – 65.6
	$[Dy_3]_{prim}-[Dy_3]_{sec}$	-	32.1	25.0
	$[Dy_3]_{sec}-[Dy_3]_{sec'}$	-	-	0.0

Table S12. Selected geometric parameters for complex **7**.

distance	length (Å)	distance	Metals MP length (Å)	angle	Angles (°)
M-(μ_3-OH-)$_{prim}$	2.46 (±0.03)	(μ_3-OH$^-$)_MP$_{prim}$	0.99	(Nd-Nd-Nd)$_{prim}$	55.3 – 62.0
M-(μ_2-O-)$_{prim}$	2.45 (±0.07)	(μ_2-O$^-$)_MP$_{prim}$	-1.3 (±0.2)	(Nd-Nd-Nd)$_{sec}$	57.7 – 61.2
(Nd-Nd)$_{prim}$	3.9 (±0.1)	(μ_3-OH$^-$)_MP$_{sec}$	-1.05	(Nd-Nd-Nd)$_{ter}$	49.5 – 78.5
M-(μ_3-OH-)$_{sec}$	2.5 (±0.05)	(μ_2-O$^-$)_MP$_{sec}$	0.8 (±0.3)	(Nd-O-Nd)$_{ter}$	149.6
M-(μ_2-O-)$_{sec}$	2.42 (±0.06)	(μ_3-OH$^-$)_MP$_{ter}$	-0.37	[Nd$_3$]$_{prim}$-[Nd$_3$]$_{sec}$	11.3
(Nd-Nd)$_{sec}$	3.93 (±0.06)	(μ_2-O$^-$)_MP$_{ter}$	-0.3 (±0.3)	[Nd$_3$]$_{sec}$-[Nd$_3$]$_{ter}$	42.1
M-(μ_3-OH-)$_{ter}$	2.45 (±0.03)	Nd1_MP$_{tot}$	0.46	[Nd$_3$]$_{ter}$-[Nd$_3$]$_{prim'}$	61.0
M-(μ_2-O-)$_{ter}$	2.45 (±0.02)	Nd2_MP$_{tot}$	1.57		
(Nd-Nd)$_{ter}$	4.1 (±0.5)	Nd3_MP$_{tot}$	0.08		
		Nd4_MP$_{tot}$	-1.73		

Table S13. Selected refinement parameters for different refinements of complex **7**.

Parameter	[Nd$_2$]	[Dy$_2$]	[DyNd]	[(Nd$_x$Dy$_{1-x}$)(Nd$_{1-x}$Dy$_x$)]
R$_1$ (%)	5.33	5.59	5.67	5.30
wR$_2$ (%)	12.08	13.42	13.96	11.98
Max Peak (e$^-$)	1.6	2.0	2.2	1.8
Min Peak (e$^-$)	-0.7	-0.9	-0.7	-0.5
GooF	1.028	1.033	1.021	1.021

Table S14. ESEM-EDS results for the analysis of the single crystal of complex **7**.

Metal	X_{Ln}	2σ
Nd	0.527	0.012
Dy	0.484	0.011

Table S15. Summary of the stoichiometries of the Ln^{3+} complexes obtained with the H_3L^{H}, $H_3L^{p\text{-}OMe}$ and $H_3L^{o\text{-}tBu}$ ligands, listed according to the refcodes reported in the CSD. In parenthesis are indicated the ligand and the metal present in a given deposited structure. Numbers **1-8** refer to the complexes present in this work (see Table 1).

M:L STOICHIOMETRY	COMPLEX/REFCODES (H_3L^R ligand, Metal)
2:2	**5** ($H_3L^{o\text{-}tBu}$, Dy); **6** ($H_3L^{o\text{-}tBu}$, Dy); **7** ($H_3L^{o\text{-}tBu}$, NdDy);
3:3	**1** ($H_3L^{p\text{-}OMe}$, Dy)
4:3	**2** (H_3L^{H}, Dy)
6:4	**3** (H_3L^{H}, Dy); **4** ($H_3L^{p\text{-}OMe}$,Dy); DAWBAG (H_3L^{H}, Eu); DAWBEK (H_3L^{H}, Er); OZONOH (H_3L^{H}, Dy); REVWAQ (H_3L^{H}, Nd_4Na_2); REVWEU (H_3L^{H}, Nd_4Na_2)
7:4	**8** ($H_3L^{o\text{-}tBu}$, Nd)
9:4	SAWYIB (H_3L^{H}, Nd_4Na_5)
9:5	MEVMUY (H_3L^{H}, Tb)
16:4	SEDVIJ (H_3L^{H}, Nd_4Na_{12}); SEDVOP (H_3L^{H}, Nd_4Na_{12})

UV-Visible measurements and speciation

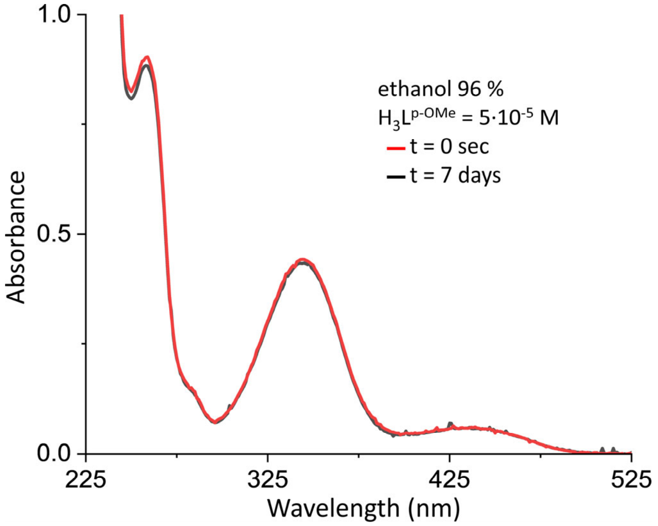

Figure S25. UV-visible spectra of $H_3L^{p\text{-}OMe}$. UV-Visible spectra were collected under stirring at 25 °C in ethanol 96 %. Spectra were collected immediately after dissolution and 7 days after dissolution using the same solution C_{Ligand} = 5·10^{-5} M, solution kept at 25 °C.

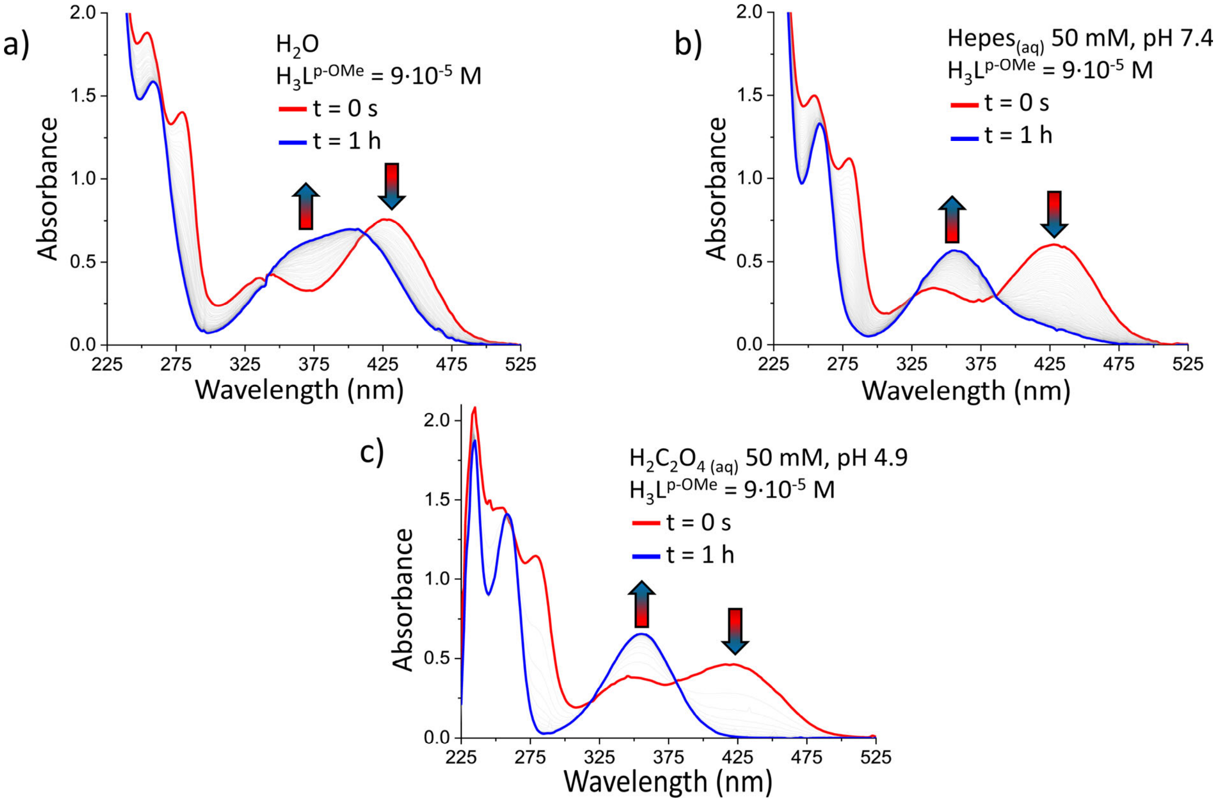

Figure S26. UV vis spectra collected over time of $H_3L^{p\text{-}OMe}$ dissolved in H_2O and aqueous buffer solutions at different pH. UV-Visible spectra were collected under stirring at 25 °C in H_2O (a), in 50 mM aqueous HEPES buffer at pH 7.4 (b) and in 50 mM aqueous oxalic acid buffer at pH 4.9 (c). UV-Visible spectra were collected every 30 s for 1h.

Separation experiments

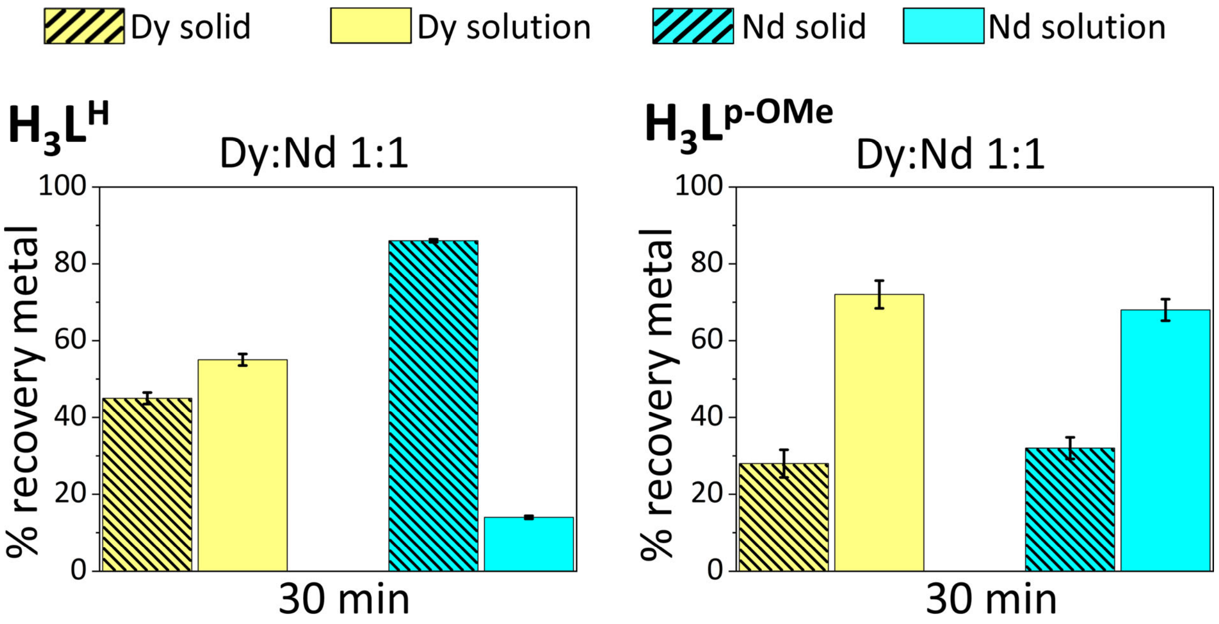

Figure S27. ICP-OES results. Weight percent of Nd and Dy recovered from the solution and solid phases for two different stoichiometric ratios in ethanol after mixing for 30 min H_3L^{H} (left)/ $H_3L^{p\text{-}OMe}$ (right), Nd^{3+}, Dy^{3+} and triethylamine in 1.05:0.5:0.5:3.15 stoichiometric ratios. The metal cations were added as nitrate salts.

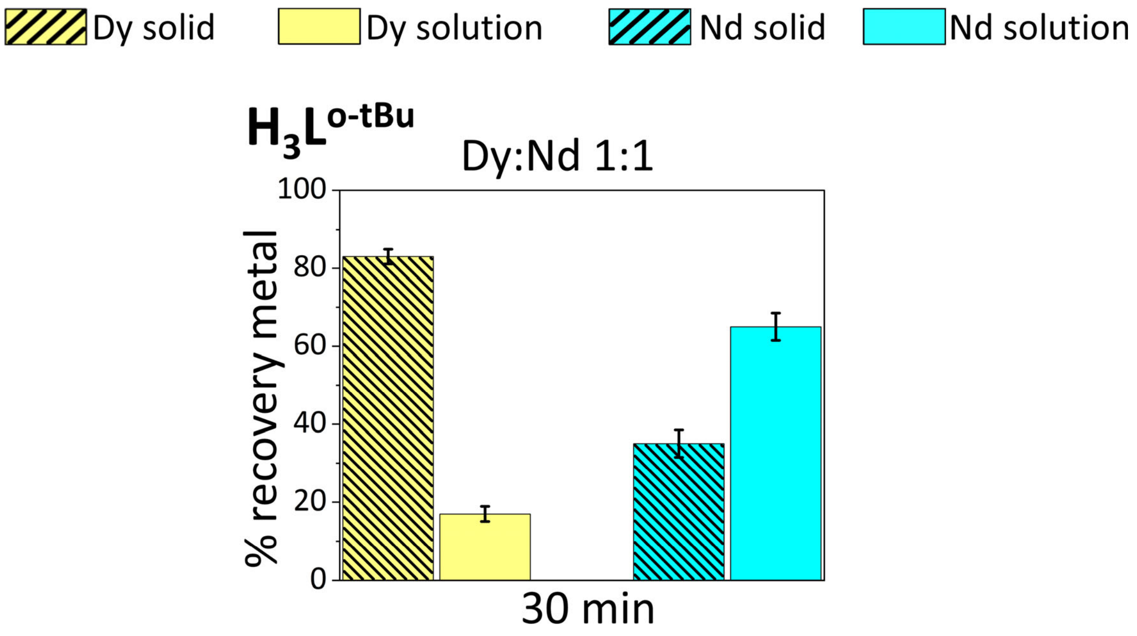

Figure S28. ICP-OES results. Weight percent of Nd and Dy recovered from the solution and solid phases for two different stoichiometric ratios in ethanol after mixing for 30 min $H_3L^{o\text{-}tBu}$, Nd^{3+}, Dy^{3+} and triethylamine in 1.05:0.5:0.5:3.15 stoichiometric ratios. The metal cations were added as nitrate salts.

ESI-MS of Dy, Nd, and Y complexes with H_3L^R

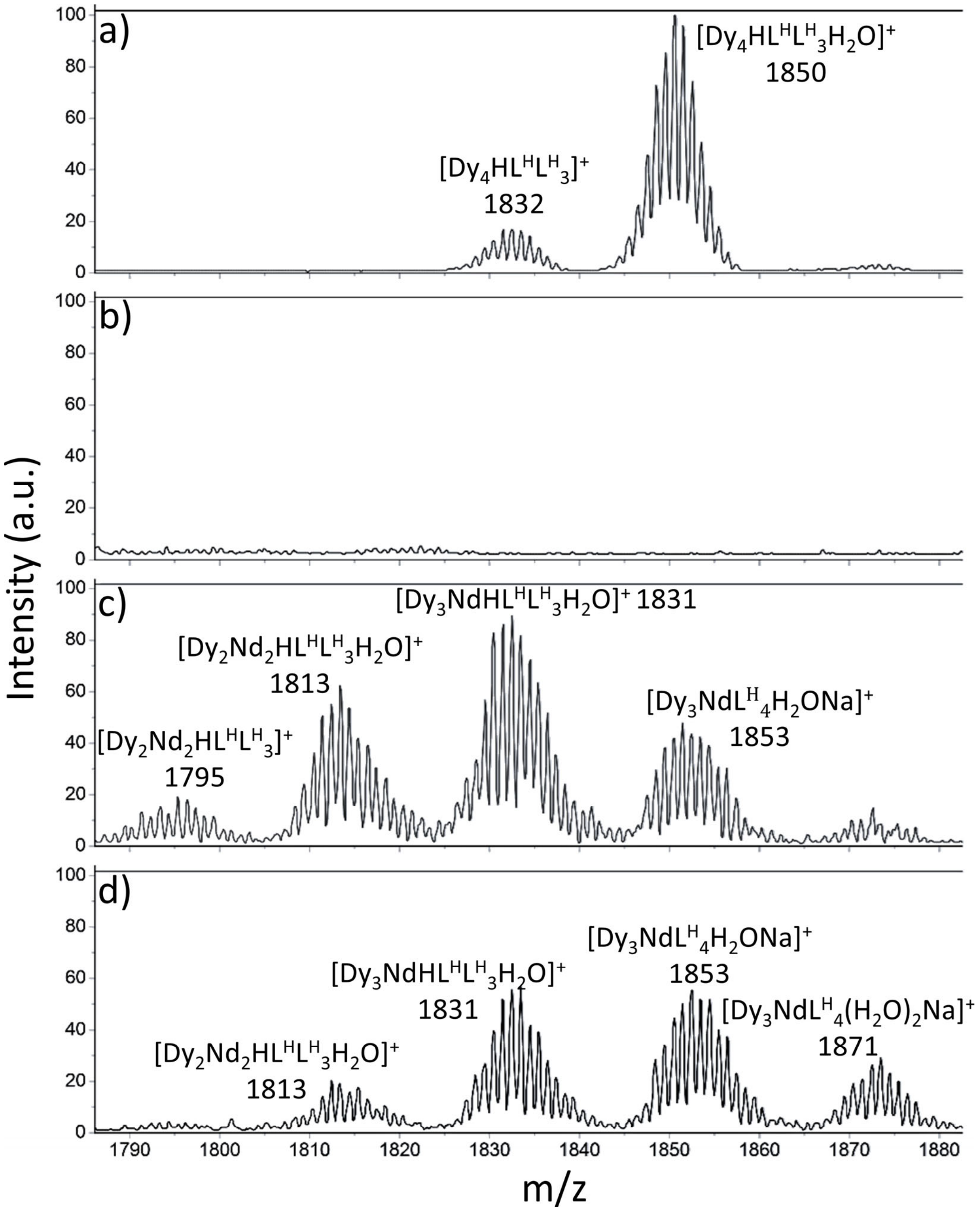

Figure S29. ESI-MS (+) spectra of the crude reaction mixture of H_3L^H with dysprosium (a) and neodymium (b), as well as the supernatant (c) and the solid (d) phases obtained from the separation of neodymium and dysprosium (H_3L^H:Nd:Dy 1.05:0.5:0.5, C_{Dy}=C_{Nd}=10 mM). Enlargement in the 1790-1880 m/z region of the spectra in Figure 9 (above panel) in the main text.

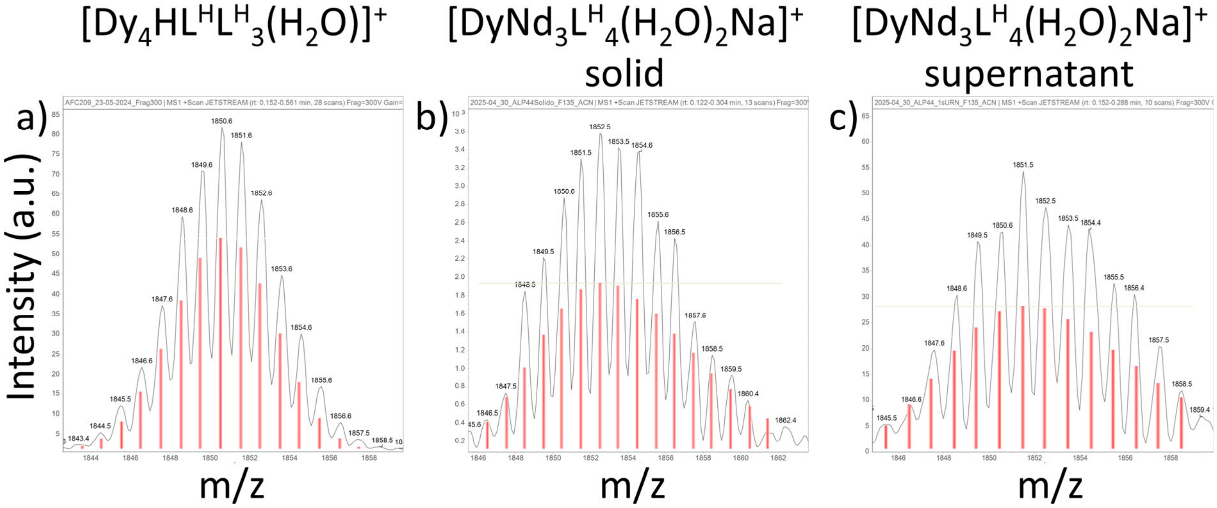

Figure S30. ESI-MS (+) spectra showing the experimental (black) and simulated (red) isotopic distributions of the main Dy-containing species. Spectrum of the Dy^{3+}-H_3L^H system, showing the expected isotopic pattern for a complex with a single lanthanide ion (a). Spectra of the supernatant and solid fractions, respectively, obtained after a separation experiment involving both Nd^{3+} and Dy^{3+} (H_3LH:Nd:Dy = 1.05:0.5:0.5; C_{Nd}=C_{Dy}=10 mM), (b) and (c).

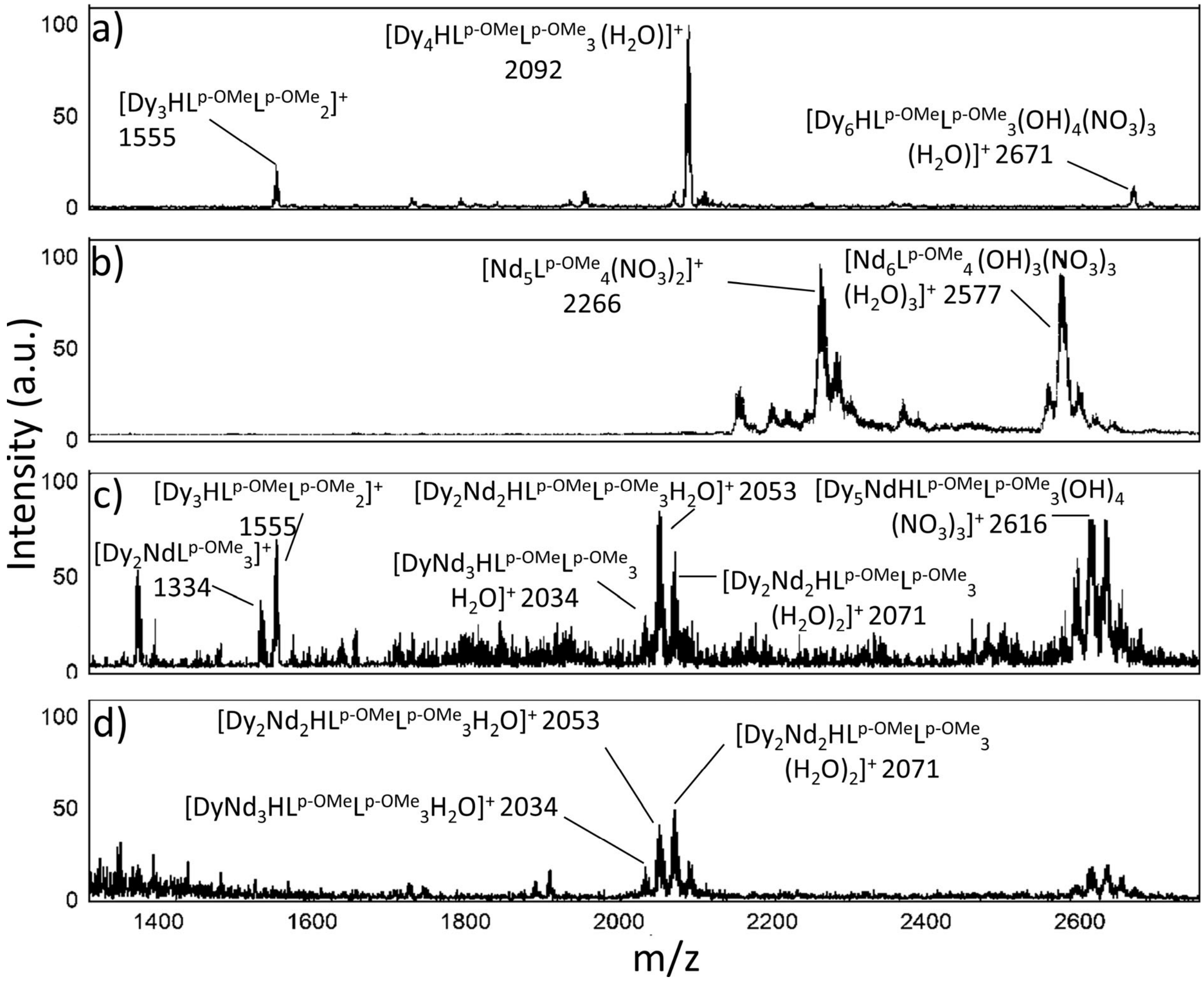

Figure S31. ESI-MS (+) spectra of the crude reaction mixture of $H_3L^{p\text{-}OMe}$ with dysprosium (a), neodymium (b), together with the liquid (c) and solid (d) phases obtained from the separation of neodymium and dysprosium ($H_3L^{p\text{-}OMe}$:Nd:Dy 1.05:0.5:0.5, C_{Dy}=C_{Nd}=10 mM).

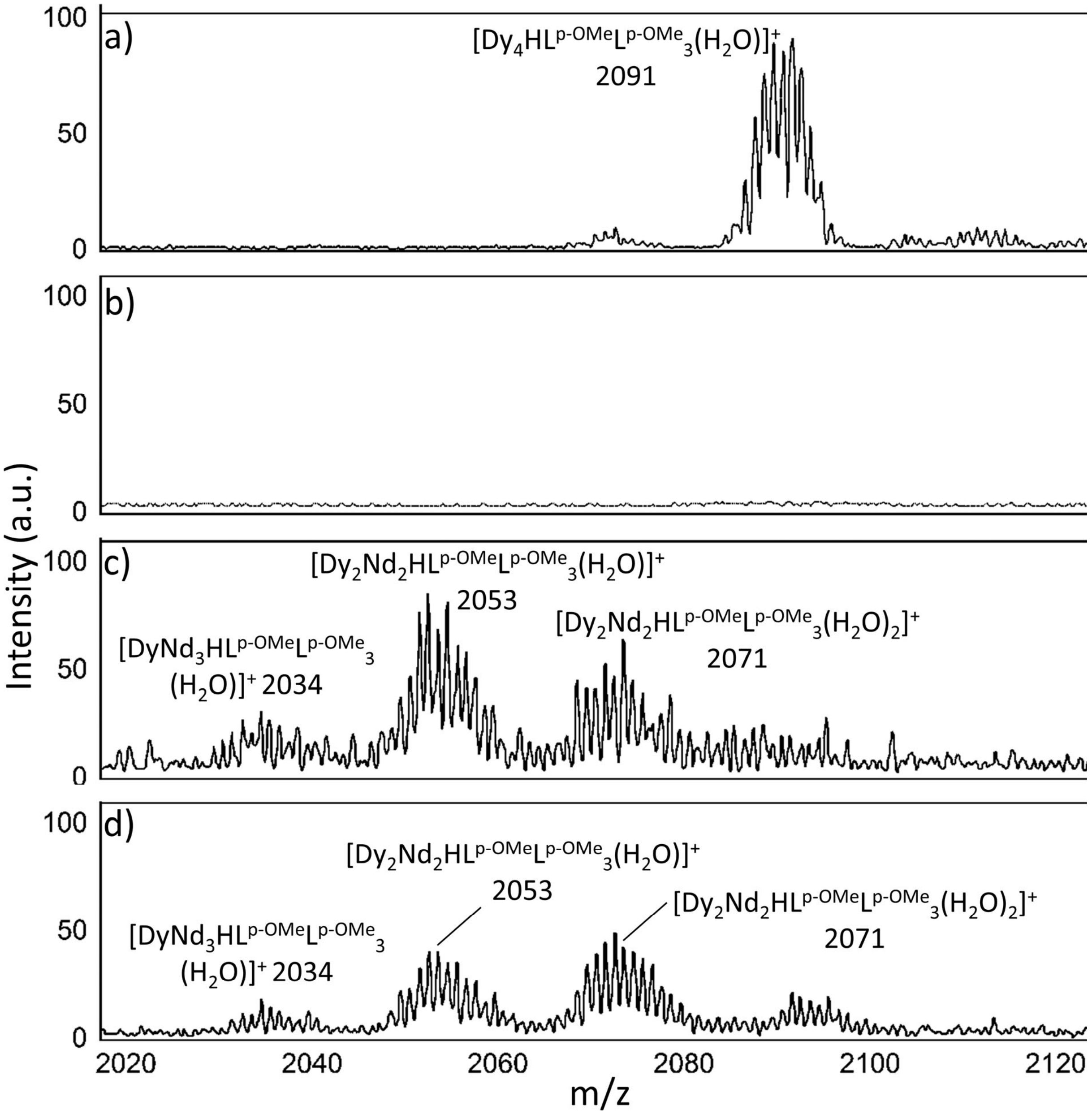

Figure S32. ESI-MS (+) spectra of the crude reaction mixture of $H_3L^{p\text{-}OMe}$ with dysprosium (a) and neodymium (b), as well as the supernatant (c) and the solid (d) phases obtained from the separation of neodymium and dysprosium ($H_3L^{p\text{-}OMe}$:Nd:Dy 1.05:0.5:0.5, C_{Dy}=C_{Nd}=10 mM). Enlargement in the 2020-2120 m/z region of the spectra in Figure S27.

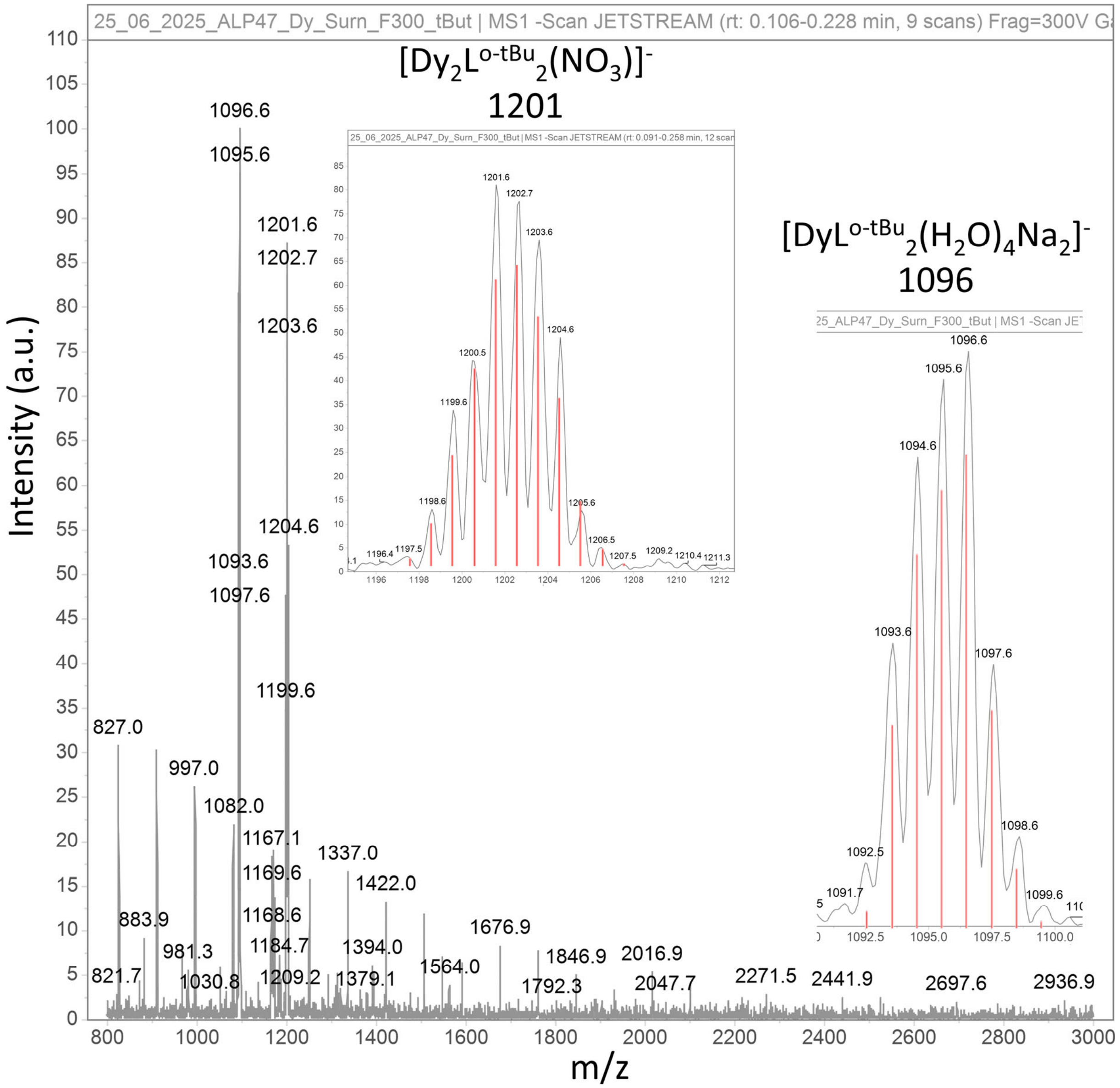

Figure S33. ESI-MS (-) spectrum of the supernatant from the Dy separation experiment using $H_3L^{o\text{-}tBu}$. The insets show two isotopic distributions: the experimental one (black) and the simulated one (red).

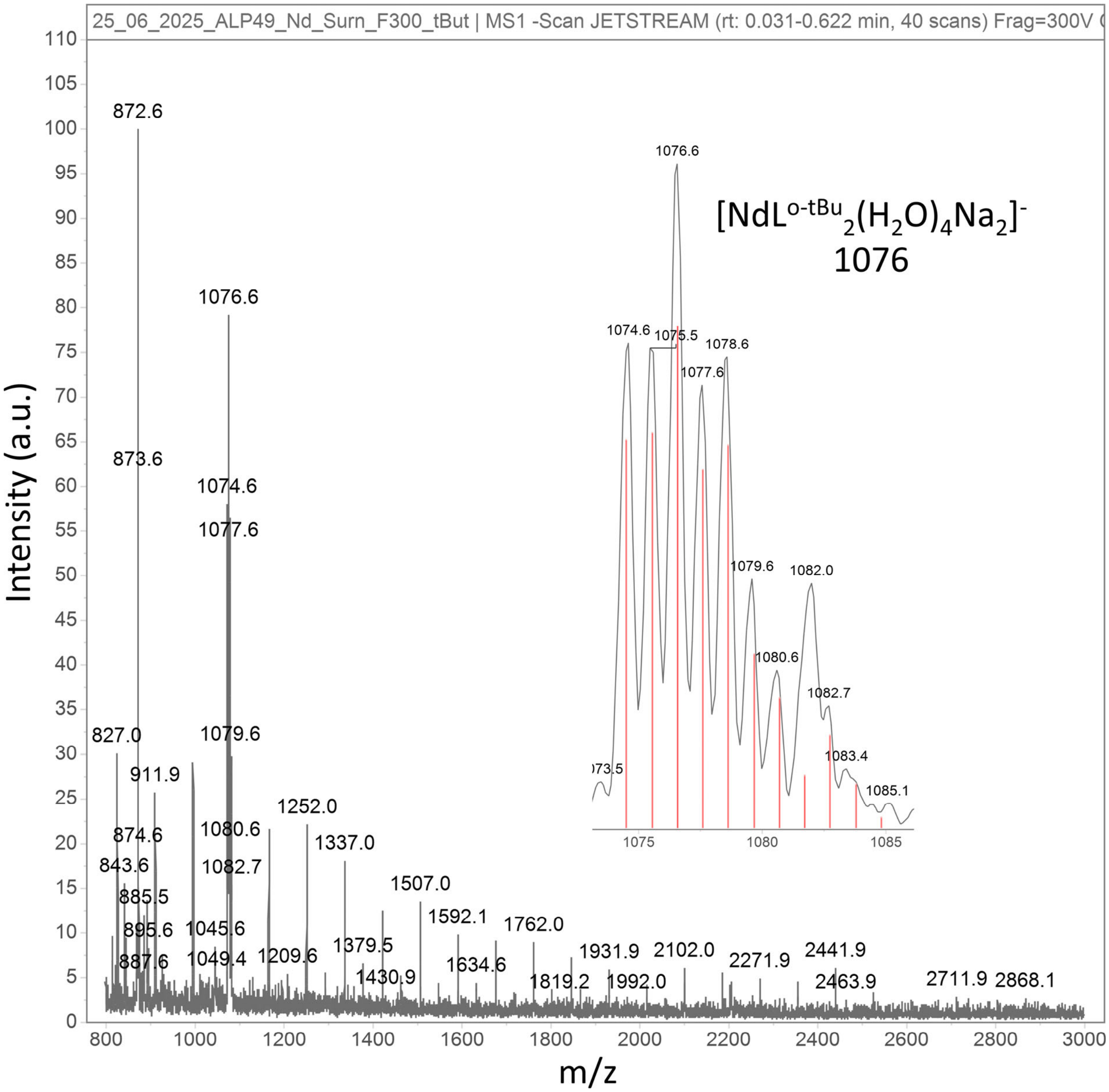

Figure S34. ESI-MS (-) spectrum of the supernatant from the Nd separation experiment using $H_3L^{o\text{-}tBu}$. The insets show two isotopic distributions: the experimental one (black) and the simulated one (red).

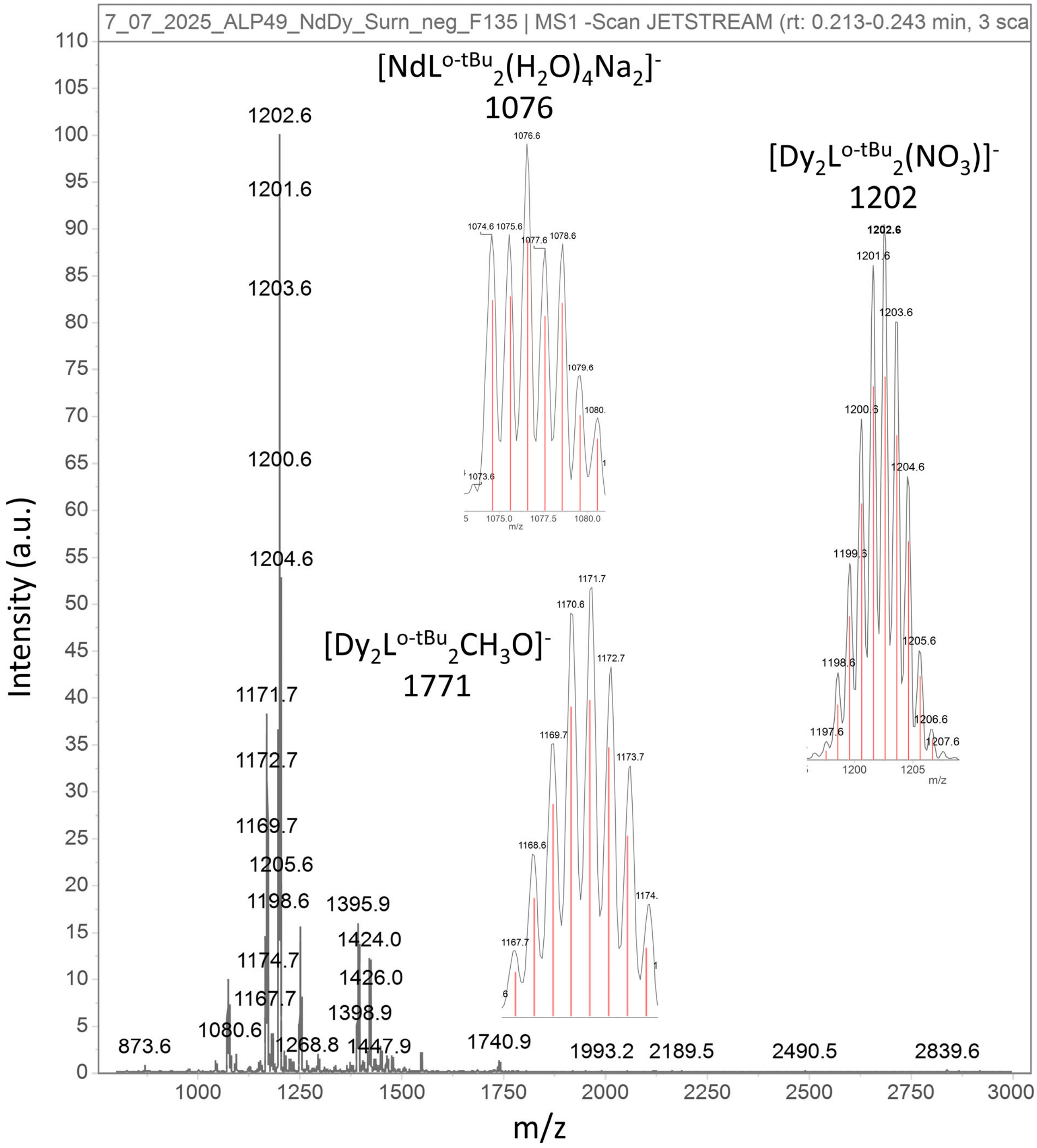

Figure S35. ESI-MS (-) spectrum of the solid from the Dy–Nd separation experiment using $H_3L^{o\text{-}tBu}$. The insets show two isotopic distributions: the experimental one (black) and the simulated one (red).

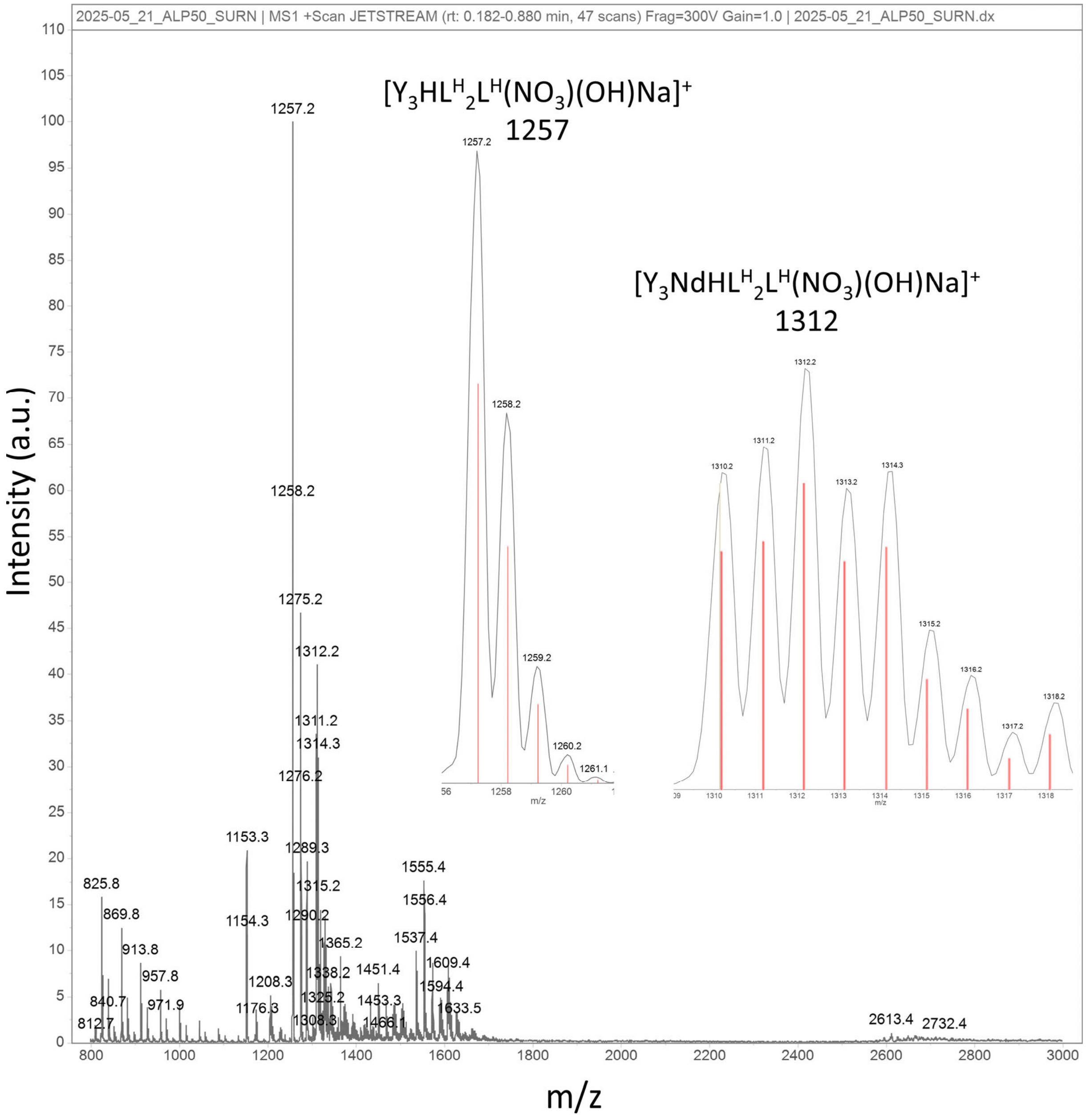

Figure S36. ESI-MS (+) spectrum of the supernatant from the Y–Nd separation experiment using H_3L^H. The insets show two isotopic distributions: the experimental one (black) and the simulated one (red). The shift in the isotopic pattern indicates that when both Y and Nd are present in the same complex (right), the distribution differs from that of a species containing only Y (left).

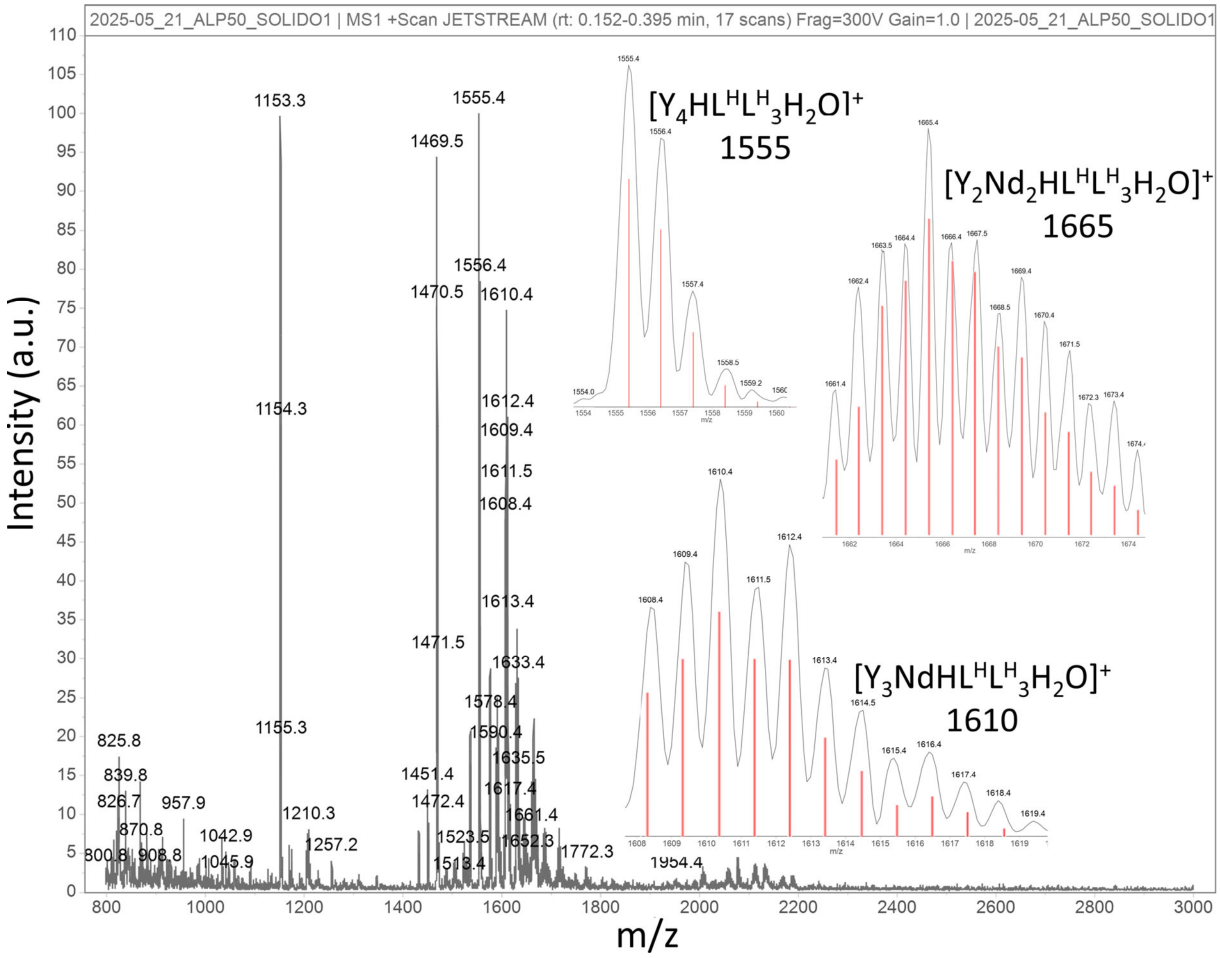

Figure S37. ESI-MS (+) spectrum of the solid from the Y–Nd separation experiment using H_3L^H. The insets show two isotopic distributions: the experimental one (black) and the simulated one (red). The shift in the isotopic pattern indicates that when both Y and Nd are present in the same complex (right and bottom), the distribution differs from that of a species containing only Y (top)

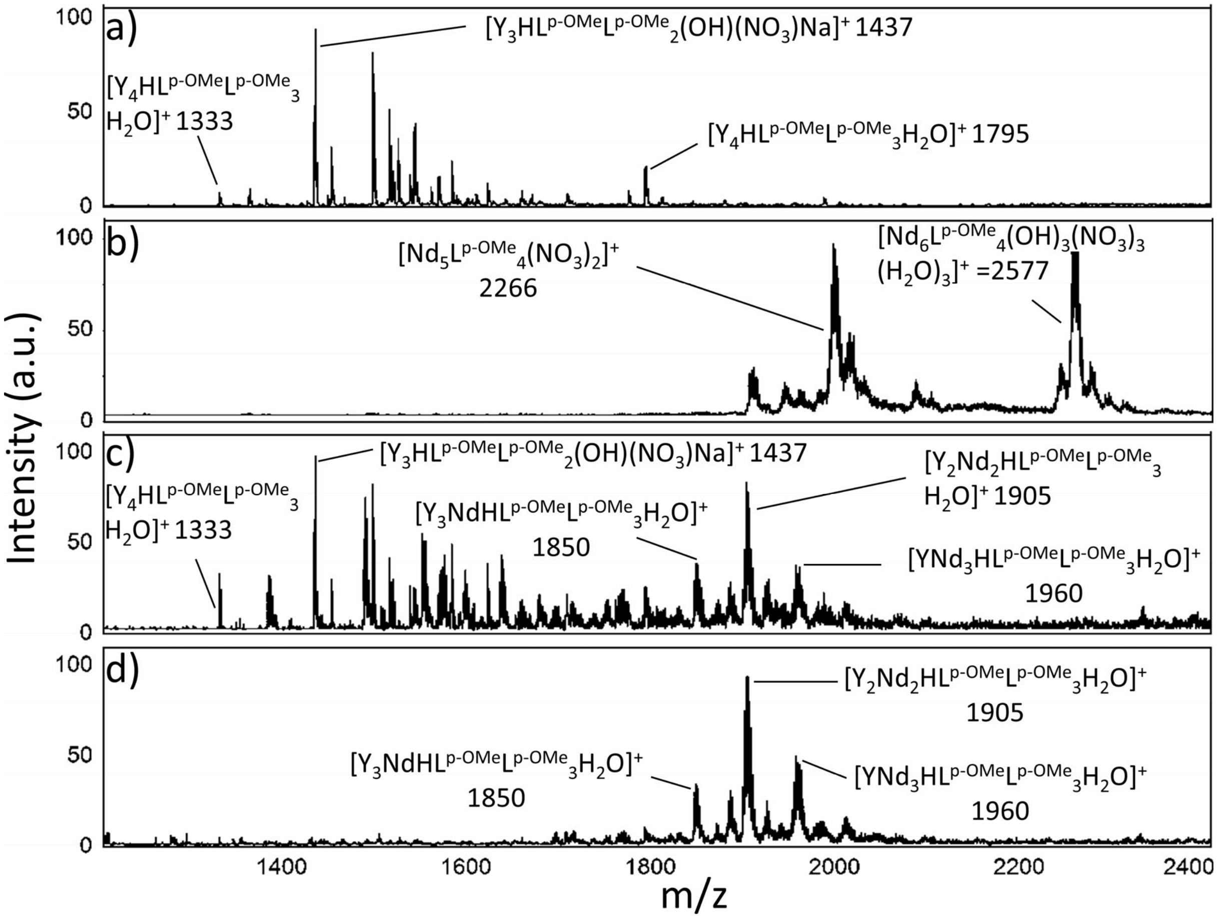

Figure S38. ESI-MS (+) spectra of the crude reaction mixture of $H_3L^{p\text{-}OMe}$ with yttrium (a) and neodymium (b), as well as the supernatant (c) and the solid (d) phases obtained from the separation of neodymium and yttrium ($H_3L^{p\text{-}OMe}$:Nd:Y 1.05:0.5:0.5, C_Y=C_{Nd}=10 mM).

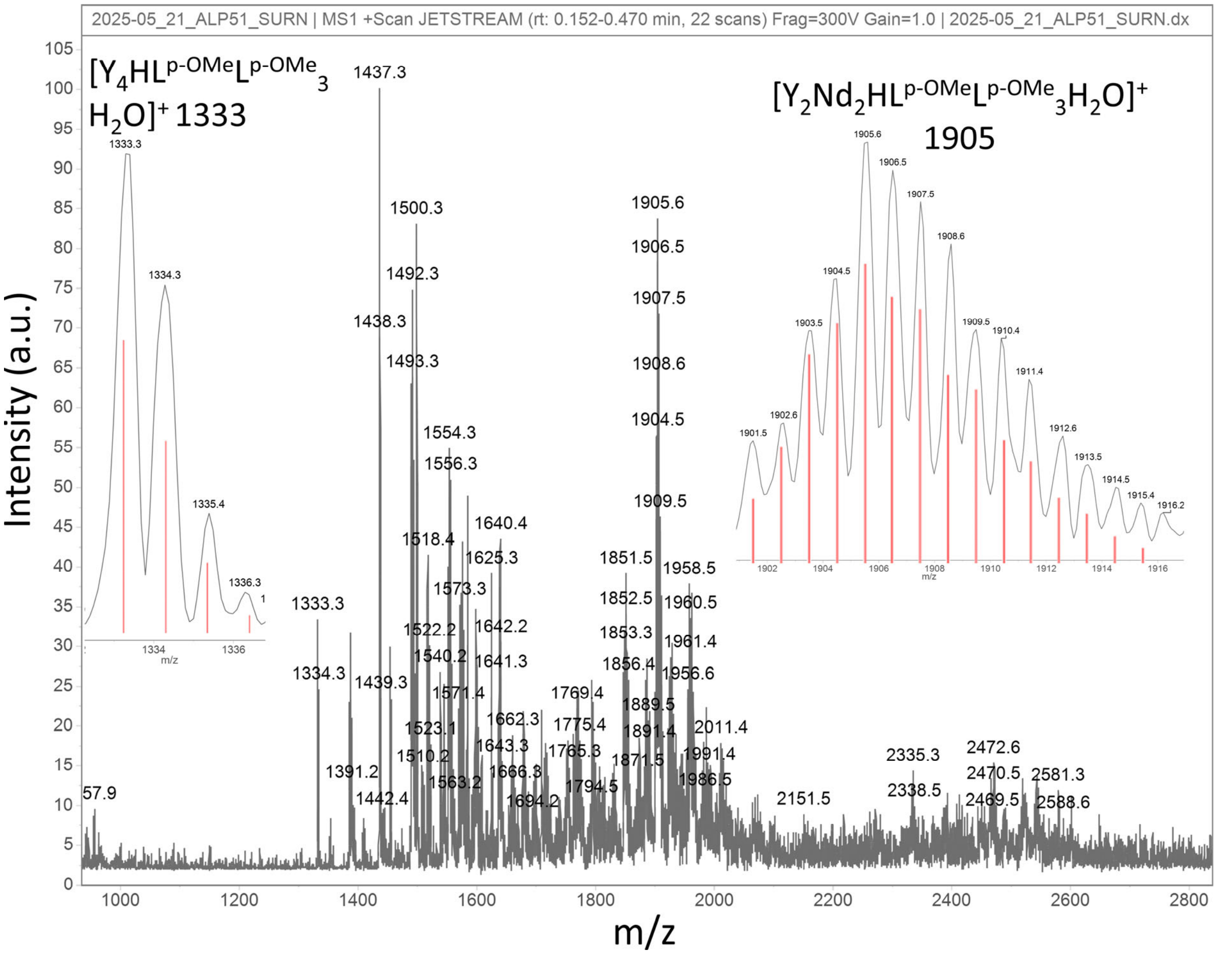

Figure S39. ESI-MS (+) spectrum of the solid from the Y–Nd separation experiment using $H_3L^{p\text{-}OMe}$. The insets show two isotopic distributions: the experimental one (black) and the simulated one (red). The shift in the isotopic pattern indicates that when both Y and Nd are present in the same complex (right), the distribution differs from that of a species containing only Y (left).

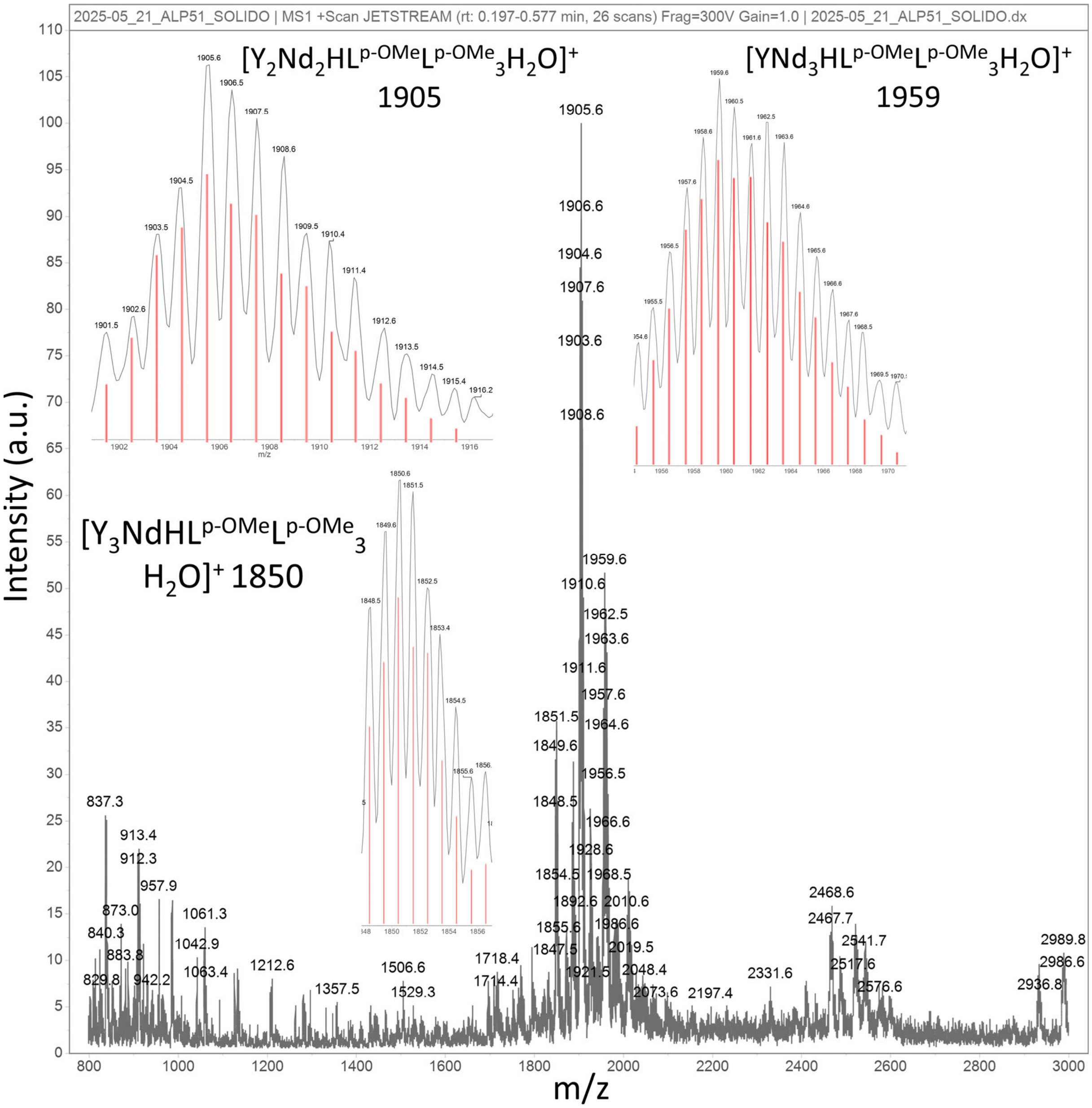

Figure S40. ESI-MS (+) spectrum of the solid from the Y–Nd separation experiment using $H_3L^{p\text{-}OMe}$. The insets show two isotopic distributions: the experimental one (black) and the simulated one (red).

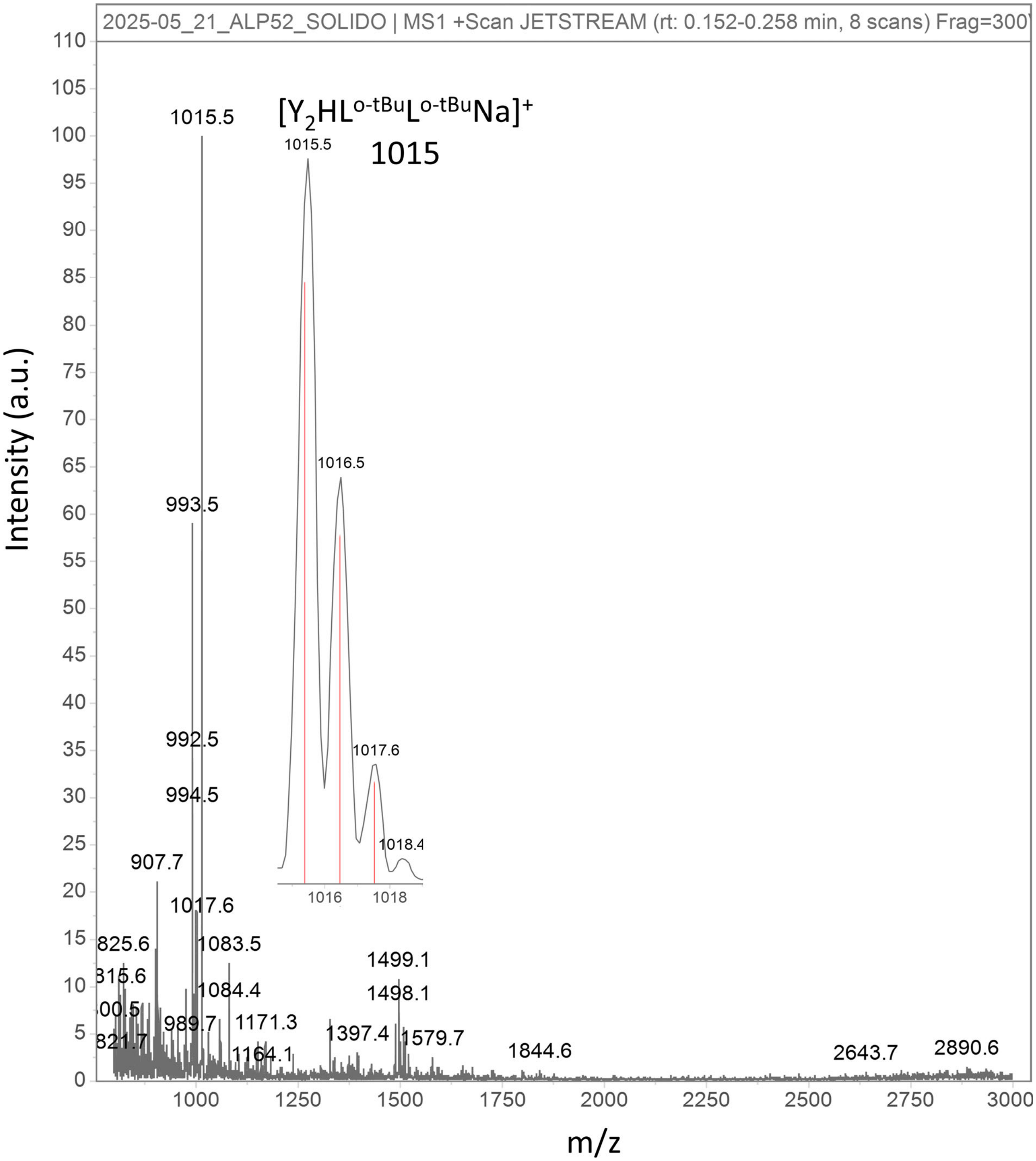

Figure S41. ESI-MS (+) spectrum of the supernatant from the Y separation experiment using $H_3L^{o\text{-}tBu}$. The insets show two isotopic distributions: the experimental one (black) and the simulated one (red).

References

(1) Casanova, D.; Alemany, P.; Bofill, J. M.; Alvarez, S. Shape and Symmetry of Heptacoordinate Transition-Metal Complexes: Structural Trends. *Chemistry – A European Journal* **2003**, *9* (6), 1281–1295. DOI: 10.1002/chem.200390145

(2) Casanova, D.; Llunell, M.; Alemany, P.; Alvarez, S. The Rich Stereochemistry of Eight-Vertex Polyhedra: A Continuous Shape Measures Study. *Chemistry – A European Journal* **2005**, *11* (5), 1479–1494. DOI: 10.1002/chem.200400799
